# The novel gene *BrMYB2*, located on chromosome A07, with a short intron 1 controls the purple-head trait of Chinese cabbage (*Brassica rapa* L.)

**DOI:** 10.1038/s41438-020-0319-z

**Published:** 2020-07-01

**Authors:** Qiong He, Junqing Wu, Yihua Xue, Wenbin Zhao, Ru Li, Lugang Zhang

**Affiliations:** 1 State Key Laboratory of Crop Stress Biology for Arid Areas, College of Horticulture, Northwest A&F University, 3 Taicheng Road, Yangling, 712100 Shaanxi People’s Republic of China; 2 College of Life Sciences, Northwest A&F University, 3 Taicheng Road, Yangling, 712100 Shaanxi People’s Republic of China; 3 State Key Laboratory of Vegetable Germplasm Innovation, Tianjin, People’s Republic of China

**Keywords:** DNA recombination, Agricultural genetics, Agricultural genetics, Plant molecular biology

## Abstract

Anthocyanins are important secondary metabolites in plants, but information on anthocyanin biosynthesis mechanisms in Chinese cabbage is limited. The new purple head Chinese cabbage cultivar 11S91 was analyzed, and an R2R3-MYB regulatory gene *BrMYB2*, located on chromosome A07, controlling the dominant purple-head trait was isolated. High expression of *BrMYB2* generated a large accumulation of anthocyanins in 11S91, accompanied by highly upregulated *BrTT8*, *BrF3*′*H*, *BrDFR1*, *BrANS1*, *BrUGT*s, *BrAT*s, and *BrGST*s. 11S91 inherited the purple locus from purple trait donor 95T2-5, and they shared consensus CDSs and gDNAs with those of *BrMYB2* (*cBrMYB2* and *gBrMYB2*). Two SNPs in *cBrMYB2* in 11S91 did not cause loss of function; in addition to several SNPs at both ends of intron 1, a large deletion had occurred in intron 1 of *gBrMYB2* in 11S91. Genetic transformation of *Arabidopsis* showed that *gBrMYB2* overexpression lines presented deeper purple color and higher expression than did the c*BrMYB2* and c*Brmyb2* lines, whereas *gBrmyb2* with a long intron 1 did not cause the purple phenotype. We first show that *BrMYB2* promotes anthocyanin biosynthesis under the control of the short intron 1 of g*BrMYB2* in purple head Chinese cabbage, and *gBrmyb2* with a long intron 1 represses anthocyanin production in white head Chinese cabbage. This evidence provides a new understanding of anthocyanin biosynthesis and purple germplasm generation in *Brassica* vegetables.

## Introduction

Anthocyanins are water-soluble pigments synthesized based on the phenylpropanoid pathway involved in secondary metabolism in plants; anthocyanins not only provide vibrant colors to plants for attracting animal pollinators and for seed dispersal but also provide strong radical-scavenging abilities to protect against biotic and abiotic stresses^1,2^. Anthocyanin-rich vegetables and fruits are also gaining in popularity because of their health attributes in humans^3^.

The mechanism of anthocyanin biosynthesis and regulation has been well studied in model plant species such as *Arabidopsis*, maize, petunia, and snapdragon^4,5^ and in economically important plant species such as apple, pear, strawberry, and peach^6^. Investigations of the genetic and molecular mechanisms of anthocyanin biosynthesis in plants have led to substantial scientific breakthroughs: it has been shown that three main steps are involved in these processes^6^. First, the primary phenylpropanoid metabolic pathway supplies precursor substrates for subsequent flavonoid synthesis, including phenylalanine ammonia lyase (PAL), cinnamate 4-hydroxylase (C4H), and 4-coumarate: CoA ligase (4CL) involved in it^1^. Second, the early biosynthesis pathway provides precursor substrates for flavonol and anthocyanin synthesis, including chalcone synthase (CHS), chalcone isomerase (CHI), flavanone 3-hydroxylase (F3H), flavanone 3′-hydroxylase (F3′H), and flavonol synthase (FLS) involved in this step^7^. Third, the late biosynthesis pathway gives rise to the biosynthesis and modification of anthocyanins via processing of dihydroflavonol 4-reductase (DFR), anthocyanidin synthase (ANS), UDP-glucosyltransferase (UGT), and acyltransferase (AT)^4^. Competition between FLS and DFR produces either flavonols or anthocyanins in the following processes, respectively^1^.

Functional R2R3-MYB transcription factors (such as MYB11, MYB12, and MYB111) usually participate in the direct activation of early biosynthesis genes (EBGs) such as *CHS*, *CHI*, *F3H*, *F3*′*H*, and *FLS*, whereas *F3*′*H* and late biosynthesis genes (LBGs) such as *DFR*, *ANS*, *UGT*s, and *AT*s are activated by a MYB-bHLH-WD40 ternary complex (MBW), which is formed by an R2R3-MYB factor, a bHLH factor, and a WD40-repeat factor^8^. These regulators or complexes are able to bind to the promoters of anthocyanin biosynthesis genes (ABGs) and activate their expression; the regulatory network of flavonoid biosynthesis usually functions as a positive feedback mechanism with the participation and interaction of both positive regulators and negative regulators, governing the accumulation of and reduction in anthocyanin or proanthocyanidin^5^. In *Arabidopsis*, the following positive regulators mainly participate in the formation of the MBW complex: the R2R3-MYBs AtPAP1, AtPAP2, AtMYB113, AtMYB114, and AtTT2; the bHLHs AtTT8, AtEGL3, and AtGL3; and the WD40 factor AtTTG1^4^. Overexpression of *AtPAP1*, *AtPAP2*, *AtMYB113*, and *AtMYB114* leads to a large accumulation of anthocyanins in a TTG1- and bHLH- cooperative way^8^; the MBW complex formed by AtTT2, AtTT8, and AtTTG1 stimulates proanthocyanidin production in *Arabidopsis* seeds^9^. Moreover, AtTT8, AtGL3, and AtEGL3 show redundant functions during regulation^5^. Negative regulatory factors contain R3-MYBs AtMYBL2 and AtCPC and lateral organ boundary domain (LBD) factors AtLBD37, AtLBD38, and AtLBD39; repression processes of AtMYBL2 and AtCPC in anthocyanin biosynthesis include inhibition of ABGs activation and suppression of MBW complex activity, and similar regulatory mechanisms associated with AtLBD37, AtLBD38, and AtLBD39 have been reported^10^. Usually, activation of positive R2R3-MYB factors results in anthocyanin accumulation in plants, and their functional loss will lead to color loss in plants^6^. For example, loss of function of the *MYB* promoter occurs by a retrotransposon insert in grape^11^ and by methylation in pear^12^. Similarly, purple cabbage is created by either substitution of the promoter or deletion of *BoMYBL2-1*^13^. In addition, insertion of DNA transposons into *bHLH2* leads to a dramatic decrease in red pigments and pale flowers in morning glory^14^. These findings suggest that the diversity of mutations in anthocyanin biosynthesis is different and nondirective in plants.

Research on anthocyanins has mainly focused on mapping, component identification, and identifying expression patterns of ABGs in natural mutant *Brassica* plants. Mapping has shown that the purple trait is a dominant trait; however, the loci differ across various *Brassica* species. The purple trait in purple cauliflower (*Brassica oleracea* L. var. *botrytis*) is controlled by *BoMYB2*^15,16^; red cabbage (*B. oleracea* L. var. *capitata*), *BoMYB2* or *BoMYBL2.1*^13,17,18^; zicaitai (*B. rapa* L. ssp. *chinensis* var. *purpurea*), *BrbHLH49*, *BrEGL3.2* and *BrMYBL2.1*^19–21^; purple bok choy (*B. rapa* L. ssp. *chinensis*), *BrMYB73*^22^; purple tumorous stem mustard (*Brassica juncea* var. *tumida* Tsen et Lee), *BjTT8*^23^; purple Chinese cabbage (created by interspecific hybridization), the R2R3-MYB gene *c3563g1i2* from the B genome of *Brassica*^7^; purple Kohlrabi (*B. oleracea* var. *gongylodes* L.), *BoPAP2* and *BoTT8*^24,25^; and purple *B. juncea*, *BjP11*^26^. The main anthocyanins in *Brassica* plants are highly glycosylated and acylated cyanidins with high stability under acidic and low-temperature conditions^27^. The main mechanism of ABGs activated by the MBW complex during anthocyanin biosynthesis in *Brassica* crops is believed to be similar across the aforementioned plant species^6^. Thus, information on new locus mining, component identification, and regulatory mechanism prediction will facilitate the discovery of new genes and the understanding of anthocyanin biosynthesis in *Brassica* species. Chinese cabbage (*B. rapa* L. ssp. *pekinensis*) is an important vegetable with white, yellow, orange or green heads and is widely cultivated in Asian countries; however, anthocyanin-rich Chinese cabbage is important due to the absence of novel natural mutants. Hence, purple head Chinese cabbage is usually bred from other purple varieties and species. For example, a purple head Chinese cabbage generated from interspecific hybridization between Chinese cabbage (2*n* = AA = 20) and red leaf mustard (*B. juncea*, 2*n* = AABB = 36) had the purple gene on chromosome A02^28^, which was different from our previous predictions^27,29^. Therefore, exploration of different purple head Chinese cabbage lines with various anthocyanin accumulation mechanisms is important.

In this study, the new purple head Chinese cabbage bred by the intersubspecific hybridization between white head Chinese cabbage (2*n* = AA = 20) and purple flowering Chinese cabbage (2*n* = AA = 20) provides an excellent opportunity to explore the molecular mechanism of anthocyanin biosynthesis. We first performed fine mapping and homology gene screening to determine the candidate gene of purple head Chinese cabbage and verified it using genetic transformation and complementary hybridization approaches. The goal was to gain new insight into the anthocyanin biosynthesis mechanism and provide a theoretical basis for the future breeding of purple *Brassica* plants.

## Results

### Genetic and physical mapping of *BrPur*

**Fig. 1:**
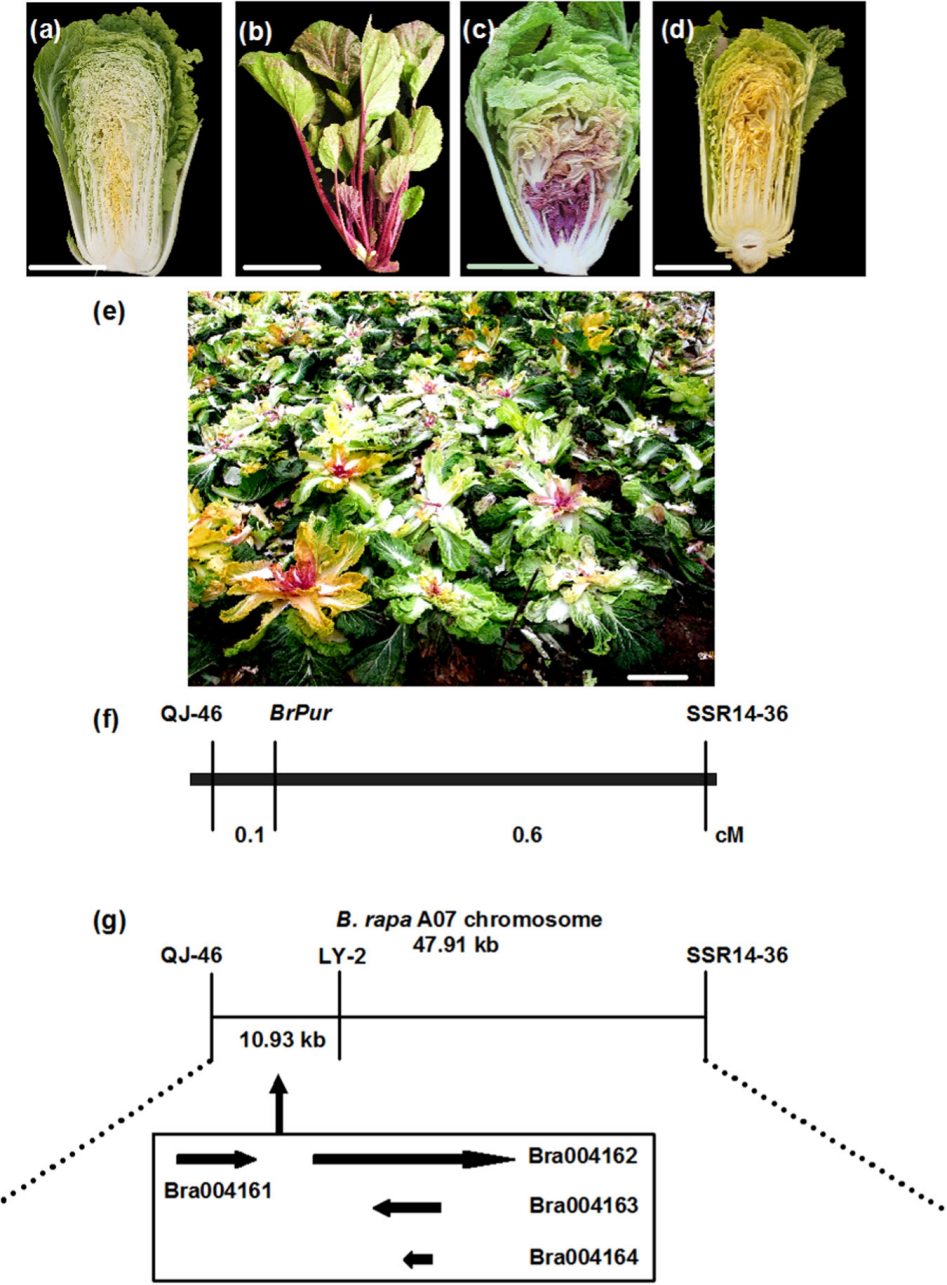
Phenotype, genetic map, and identification of candidate *BrPur* genes in purple head Chinese cabbage. **a** 94S17 was the female parent of 11S91, with a white head. **b** 95T2-5, a purple flowering Chinese cabbage, was the male parent. **c** 11S91, with stable inheritance of purple head leaves, was bred from the hybridization of 94S17 and 95T2-5 and continuous self-breeding. **d** 14S162 was the female parent used for creating the mapping population. **e** Selected individuals of the F_2_ population formed by crossing 14S162 and 11S91. **f** Genetic map of *BrPur* based on fine mapping with markers QJ-46 (0.1 cM) and SSR14-36 (0.6 cM). **g** Physical map showing the candidate region of the *BrPur* locus, which was delimited to a 10.93 kb region on chromosome A07, with four predicted genes between markers QJ-46 and LY-2. The scale bar is 10 cm in (**a**–**d**)

Head color investigations showed that the segregation ratio of purple individuals to nonpurple individuals in the F_2_ population was consistent with the ratio of 3:1^29^, and two other F_2_ populations also yielded the same results, which together indicated that the purple-head trait was controlled by a single dominant gene. Two markers, CL-12 and B214-87, were closely linked to *BrPur*, with genetic distances of 3.1 cM and 3.5 cM (physical distance of 577.235 kb), respectively^29^. Additional molecular markers were designed and tested, and fine genetic mapping showed that two markers, QJ-46 and SSR14-36, were closely linked to *BrPur*, with genetic distances of 0.1 cM and 0.6 cM (physical distance of 47.91 kb), respectively (Fig. 1f). Four predicted genes existed within a 10.93 kb region between markers QJ-46 and LY-2 on chromosome A07 in *B. rapa* genome, namely, *Bra004161*, *Bra004162*, *Bra004163*, and *Bra004164* (Fig. 1g). Among them, *Bra004162* encoded an R2R3-MYB transcription factor that is highly homologous to AtPAP1 and AtPAP2, which are involved in the formation of the MBW complex in *Arabidopsis*^6^. In combination with recent reports that expression of *BrMYB2* (Bra004162) was significantly positively correlated with total anthocyanin content in purple head Chinese cabbage^10,27^, our results suggest that *BrMYB2* is the candidate gene (*BrPur*) of the purple-head trait.

### Sequence analysis of the candidate gene and functional markers of head color

**Fig. 2:**
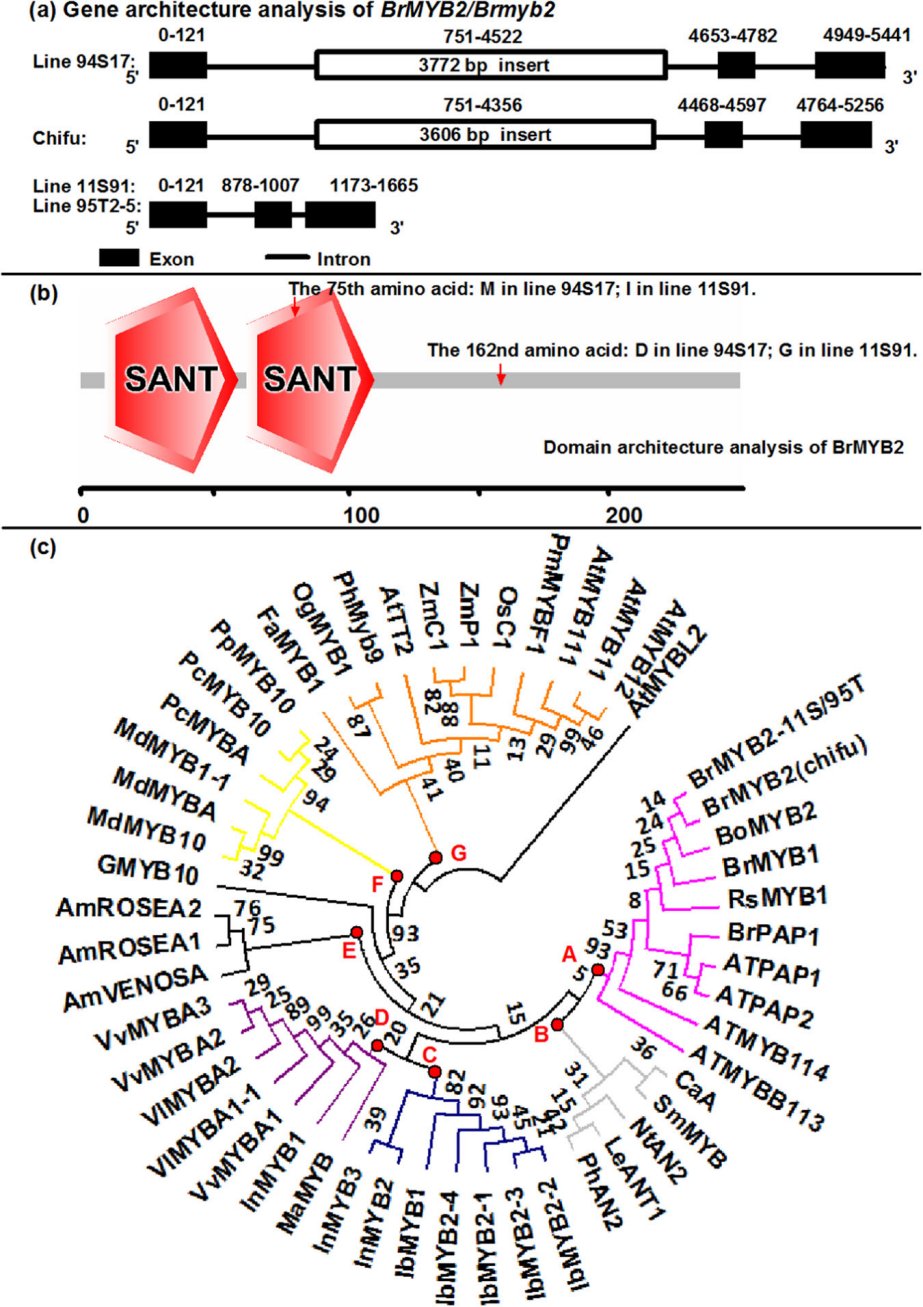
Structure comparison of *BrMYB2* and a phylogenetic tree of BrMYB2 homologs. **a** Comparison of exons and introns of *BrMYB2*. **b** Display of predicted BrMYB2 domains. **c** Phylogenetic tree of MYBs. The numbers next to the nodes indicate bootstrap values from 1000 trials, and the branches with the same color indicate that those MYBs are classified into groups. The GenBank number or BRAD ID of these proteins is supplied in Table S2

Sequence analysis showed that *BrMYB2* of 95T2-5 and 11S91 had the same coding DNA sequences (CDSs) and genomic DNA (gDNA) sequences, and their CDSs were similar to those of Bra004162 of Chifu (Figs. S1–2). The *BrPur* CDS was designated *cBrMYB2* for 11S91 and 95T2-5 and was designated *cBrmyb2* (the *Brpur* CDS) for 94S17. Two SNPs existed at the 225th bp and 485th bp sites and caused two amino acid substitutions, methionine to isoleucine and aspartic acid to glycine from 94S17 to 11S91 at the 74th and 162nd sites, respectively (Figs. 2b, S2 and S3). Both *BrMYB2* and *Brmyb2* contained an intact open reading frame and coded a deduced protein comprising 247 amino acids (Figs. 2b, S2 and S3). The MYB protein contained two conserved SANT domains (the important functional areas), namely, R2- and R3-MYB repeats (Fig. 2b). Both *gBrMYB2* and *gBrmyb2* consisted of three exons and two introns (Fig. 2a), but the gDNA length was distinctly different; the length of *gBrMYB2* in 11S91 and 95T2-5 was only 1665 bp but increased to 5441 bp in 94S17. Furthermore, their gDNAs were extremely different from that of Chifu (Figs. 2a and S2d, e). In addition to several SNPs at both ends of the first intron (intron 1) of *gBrmyb2*, a large insertion fragment of 3772 bp was found in the middle of the intron 1 in 94S17; however, the GT-AG splicing sites at both ends of the intron 1 were invariant (Figs. 2a and S2d, e). In addition, other phenotypes of Chinese cabbage, including three white-head lines (15S1080, 15S1084, and 13S1147; Fig. S1b–d), an orange-head line 13S93 (Fig. S1e), and four purple-head lines (14S838, Z33, Z190, and Z240; Fig. S1f–i), were analyzed for variation within *BrMYB2*. These lines showed sequence results identical to those of the abovementioned three lines; for example, the CDSs and gDNAs of *BrMYB2* of the purple-head lines 14S838, Z33, Z190, and Z240 were the same as those of 11S91, whereas the CDSs and gDNAs of *Brmyb2* of the nonpurple-head lines 15S1080, 15S1084, 13S1147, and 13S93 were the same as those of 94S17 (Fig. S2c–e). These results showed that the sequences of *BrMYB2* and *Brmyb2* in purple and nonpurple Chinese cabbage, respectively, were extremely conserved but different from those of Chifu.

Furthermore, based on sequence differences between *gBrMYB2* and *gBrmyb2*, two codominant markers, BrP1 and BrP2, were designed and used for PCR-based testing of the head phenotype of F_2_ individuals. Both BrP1 and BrP2 amplified large products (4080 bp, and 4122 bp, respectively) in the nonpurple plants, small products (306 bp and 348 bp, respectively) in the purple-head plants, and two products (4080 bp/306 bp and 4122 bp/348 bp, respectively) in the heterozygous plants (Fig. S4c, d). The polymorphic bands amplified with BrP1 and BrP2 were completely consistent with the head color (Fig. S4c, d), which further indicated that *BrMYB2* was the candidate gene controlling purple head color in 11S91. Moreover, the codominant markers BrP1 and BrP2 were useful in molecular marker-assisted breeding of purple head Chinese cabbage. Fifty-one MYBs from different plant species were used to construct a phylogenetic tree (Fig. 2c), which could help understand the relationship of BrMYB2 to reported anthocyanin MYBs. The amino acids of the MYBs were highly conserved and contained a common [D/E]Lx_2_[R/K]x_3_Lx_6_Lx_3_R motif in the R3 domain for interactions with R/B-like bHLH proteins^30^, but the region downstream from this motif was divergent in both sequence and length. The MYBs were classified into seven different groups (Fig. 2c), and important signature motifs of each group might affect related anthocyanin biosynthesis. MYBs in Group A mainly contained *Cruciferae* species such as cabbage, Chinese cabbage, radish, and *Arabidopsis*, and they had a common KPRPRSFT motif (but it was not present within AtMYB114) (Fig. S5); in addition, these *BrMYB*s also showed high DNA sequence similarity (Fig. S2b). The MYBs of *Solanaceae* species in Group B were adjacent to the BrMYBs, and the common motif was [K/R]PRPRTFS, with several amino acid changes (Fig. S6). Group C and Group D mainly contained MYBs from *Convolvulaceae* and *Vitaceae* plants, respectively. Notably, the MYBs in these groups mainly belonged to a family whose members might have higher sequence identity in the downstream areas (Fig. 2c). Only MYBs in Group G included different categories of plants, such as MYBs of maize and rice, which are monocots, and AtTT2, AtMYB12, AtMYB111, and AtMYB11 of *Arabidopsis*, a dicot (Fig. 2c). AtMYB111, AtMYB12, and AtMYB11 participate in the activation of EBGs in *Arabidopsis*^5^. These results indicated that MYBs related to anthocyanin production in monocots showed higher sequence similarity to MYBs of *Arabidopsis* controlling EBGs, whereas MYBs involved in anthocyanin synthesis in dicots from one family had a closer genetic relationship. Thus, BrMYB2 might affect anthocyanin biosynthesis, as it is highly similar to previously reported AtMYBs.

### Expression patterns of *BrMYB2* and related ABGs in different tissues

**Fig. 3:**
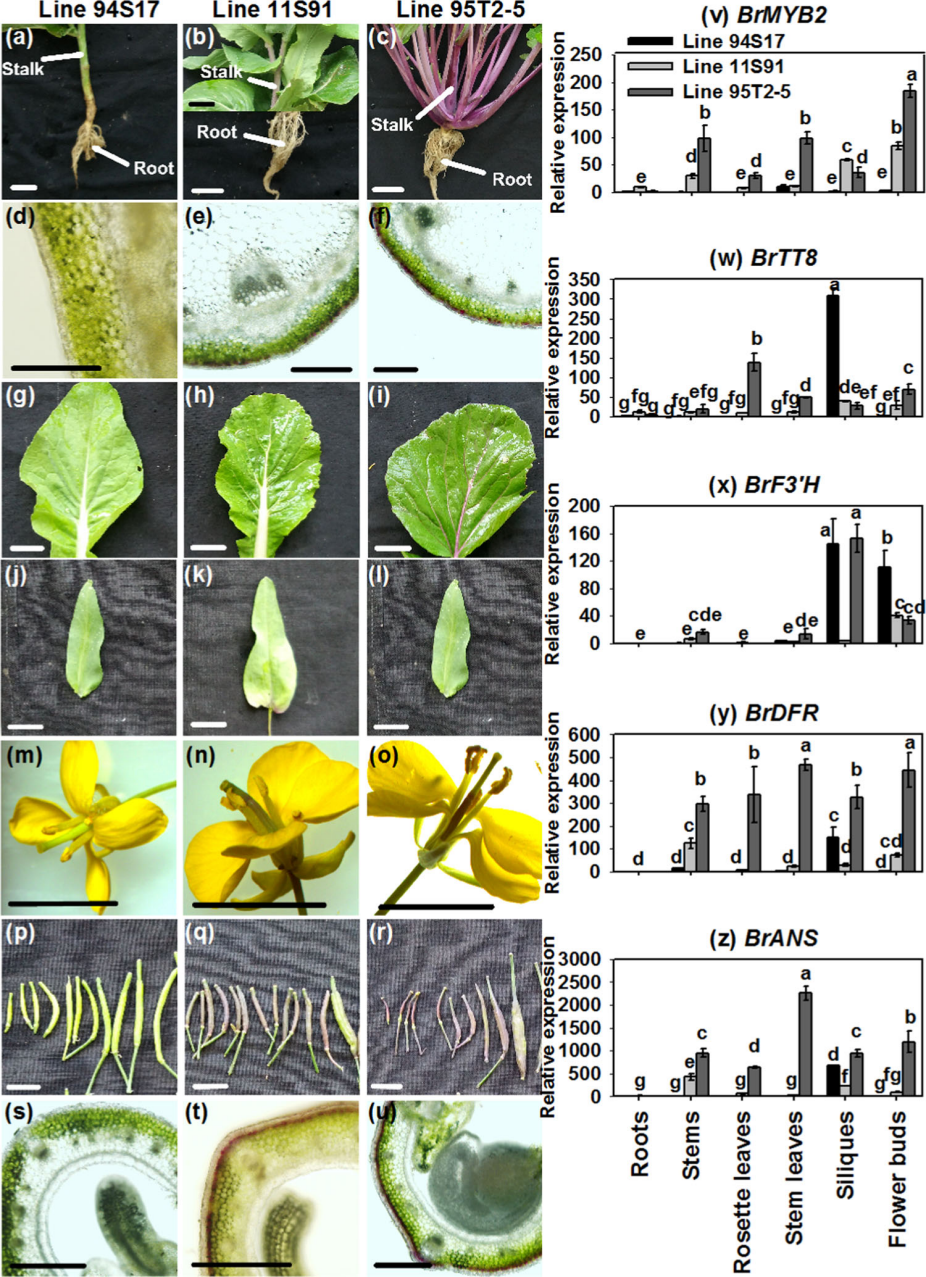
Expression patterns of *BrMYB2* and related ABGs in different tissues. **a–u** Phenotypes of different tissues of three Chinese cabbage lines; the images in each vertical sample column (left to right) were collected from 94S17, 11S91, and 95T2-5. **a**–**c** Stalks and roots. **d**–**f** Transection of stems. **g**–**i** Rosette leaves. **j**–**l** Stem leaves. **m**–**o** Flowers. **p**–**r** Siliques. **s**–**u** Transection of siliques. **v**–**z** Gene expression patterns. The rosette leaves of 94S17 were treated as controls in the data analysis; the values are presented as the means ± SDs (*n* = 3). The different letters above each column are significantly different at *p* < 0.05 according to Duncan’s test. The scale bar is 1.5 cm in (**a**–**c**) and (**g**–**t**) and 200 μm in (**d**–**f**) and (**s**–**u**).

Generally, the expression patterns of *BrMYB2* had organ and genetic specificity and were tightly correlated with the degree of purple color in different tissues. In 94S17, *BrMYB2* was significantly expressed at low levels in all nonpurple tissues, such as the roots, stems, rosette leaves, stem leaves, siliques, and flower buds (Fig. 3v). *BrMYB2* was highly expressed in the stems, rosette leaves, stem leaves, siliques, and flower buds in 95T2-5 and 11S91; there was greater *BrMYB2* expression and deeper purple color in these tissues in 95T2-5 than in 11S91 (except in the siliques, in which the expression was higher in 11S91) (Fig. 3a–u). In regard to color appearance, all tissues of 94S17; the roots of 95T2-5; and the roots, stem leaves, and rosette leaves of 11S91 showed no purple color (Fig. 3a–u). Moreover, *BrMYB2* was expressed little in these tissues (Fig. 3v). The bHLH regulatory gene *BrTT8* together with three structural ABGs, *BrF3*′*H*, *BrDFR*, and *BrANS*, was also investigated. Compared with that in 11S91, the degree of upregulation of *BrTT8* in 95T2-5 was not high, but the expression levels of the LBGs *BrDFR* and *BrANS* in 95T2-5 in all tissues were much higher than those in 11S91; the EBG *BrF3*′*H* showed significantly higher expression only in the siliques in 95T2-5 compared with 11S91 (Fig. 3w–z). Similar to *BrMYB2*, these genes showed significantly low expression in the roots, stems, rosette leaves, stem leaves, and flower buds of 94S17; however, they were highly expressed in the siliques. Previous studies indicated that *TT8*, *F3*′*H*, *DFR*, and *ANS* redundantly participated in proanthocyanidin biosynthesis and brown testa formation^31,32^. In this study, 94S17, 11S91 and 95T2-5 have brown testa. Taken together, the results show that *BrMYB2* controls anthocyanin biosynthesis in different tissues and is tightly correlated with purple color emergence in purple Chinese cabbage. In addition, related ABGs, such as *BrTT8*, *BrF3*′*H*, *BrDFR*, and *BrANS*, respond to *BrMYB2* and participate in anthocyanin biosynthesis, and they might be redundantly involved in proanthocyanidin biosynthesis during brown testa formation in three Chinese cabbage lines.

**Fig. 4:**
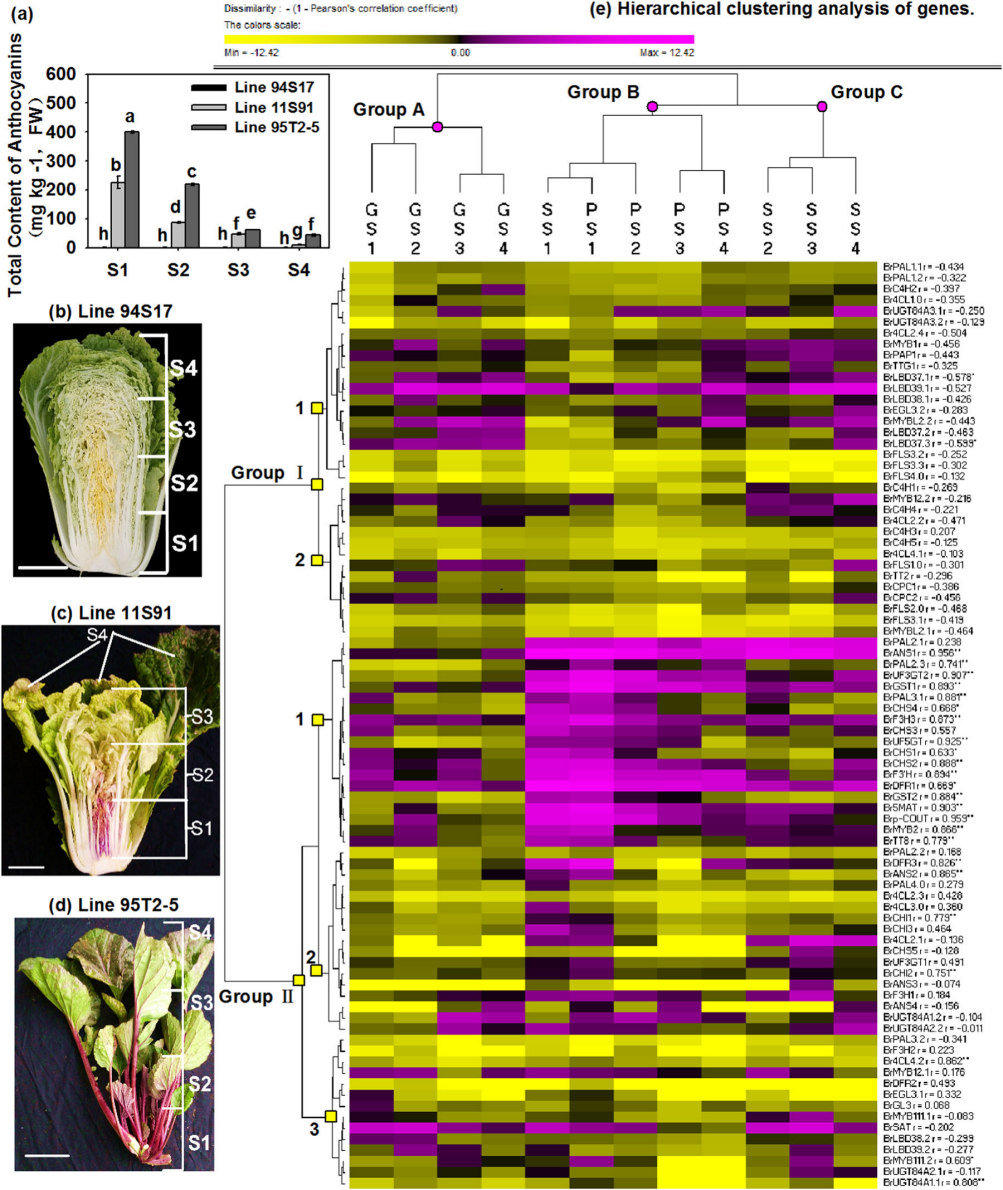
Total anthocyanin content and expression patterns of ABGs in Chinese cabbage at the head-formation stage. **a** Total anthocyanin content of samples; the values are presented as the means ± SDs (*n* = 3), and the different letters above each column are significantly different at *p* < 0.05 according to Duncan’s test. **b**–**d** The head of 11S91 was divided into four parts: S1, the interior heading leaves with deep purple color; S2, the interior leaves with light purple color; S3, the exterior heading leaves; S4, the outer functional leaves. The leaf size and positions of the 94S17 and 95T2-5 samples were the same as those of 11S91. **e** Hierarchical clustering analysis of gene expression patterns. The expression data were log2-normalized, clustered using PermutMatrix software^61^ and analyzed with the Pearson distance and Ward’s method. The magenta boxes indicate upregulation, and the yellow boxes indicate downregulation; the color brightness is directly proportional to the expression ratio. The first capital letters ‘G’, ‘P,’ and ‘S’ are different leaf tissues of 94S17, 95T2-5, and 11S91, respectively. The relative coefficient ‘r’ with ‘*’ and ‘**’ indicates that gene expression is significantly and highly significantly correlated with total anthocyanin content at the levels of 0.05 and 0.01, respectively

Similar to the determination of different tissues, 95T2-5 had the highest extent of total anthocyanin content, ranging from 42.675 mg kg^−1^ in S4 external leaves to 399.454 mg kg^−1^ in S1 inner leaves during the mature period, followed by 11S91, whose content ranged from 10.080 mg kg^−1^ in S4 external leaves to 225.544 mg kg^−1^ in S1 inner leaves; however, 94S17 accumulated a very low amount of anthocyanins in the head (Fig. 4a–d). To better understand the anthocyanin biosynthesis mechanism and the role of *BrMYB2*, a total of 84 ABGs, including 21 phenylpropanoid metabolic pathway genes (PMPGs) (*BrPAL*s, *BrC4H*s, and *Br4CL*s), 18 EBGs (*BrCHS*s, *BrCHI*s, *BrF3H*s, and *BrF3*′*H*), 19 LBGs (*BrDFR*s, *BrANS*s, *BrUGT*s, and *BrAT*s), and two transport genes (*BrGST*s), as well as 13 positive regulatory genes and 11 negative regulatory genes identified recently in Chinese cabbage^10^, were tested to explore their expression patterns in mature heads of 11S91 (Fig. 4e and Table S3). In the figure, the columns reflect different samples, the rows display various genes, and the samples with similar gene expression patterns are aggregated on a branch. Group A consists of only heading leaves S1–S4 from 94S17, Group B includes leaves S1–S4 from 95T2-5 and S1 leaves from 11S91, and Group C contains only heading leaves S2–S4 from 11S91 (Fig. 4e). These results further showed that a significant change in the anthocyanin regulatory mechanism occurred between 94S17 and purple-head lines during head maturity, and the purple S1 leaves of 11S91 inherited the characteristic of anthocyanin biosynthesis from the purple trait donor 95T2-5.

In the rows, the genes were classified into two main groups. Group I contains genes with similar expression levels in both white and purple head Chinese cabbage (Fig. 4e). For example, genes in Group I.1 showed relatively high or similar expression in 94S17 and 11S91 and low expression in 95T2-5. These genes include the PMPGs *BrPAL1.1*, *BrPAL1.2*, *BrC4H2*, *Br4CL1.0*, and *Br4CL2.4*; the EBGs *BrFLS3.2*, *BrFLS3.3*, and *BrFLS4.0*; the LBGs *BrUGT84A3.1* and *BrUGT84A3.2*; the positive regulatory genes *BrMYB1*, *BrPAP1*, *BrTTG1*, and *BrEGL3.2*; and the negative regulatory genes *BrMYBL2.2*, *BrLBD37.1*, *BrLBD37.2 BrLBD37.3*, *BrLBD38.1*, and *BrLBD39.1*. Notably, the gene expression in this group was upregulated in most samples but negatively correlated with total anthocyanin content (Fig. 4e). The genes in Group I.2 were downregulated in nearly all samples and negatively correlated with total anthocyanin content. These genes included the PMPGs *BrC4H1*, *BrC4H3*, *BrC4H4*, *BrC4H5, Br4CL2.2*, and *Br4CL4.1*; the EBGs *BrFLS1.0*, *BrFLS2.0*, and *BrFLS3.1*; the positive regulator genes *BrMYB12.2* and *BrTT2*; and the negative regulator genes *BrMYBL2.1*, *BrCPC1*, and *BrCPC2*. On the contrary, the genes in Group II mainly showed higher expression in the purple-head lines than in the white-head lines. Note that genes in Group II.1 were extremely highly expressed in purple-head lines and were significantly correlated with total anthocyanin content (except for *BrPAL2.1* and *BrCHS3*). These genes included the PMPGs *BrPAL2.3* and *BrPAL3.1*; the EBGs *BrCHS1*, *BrCHS2*, *BrCHS4*, *BrF3H3*, and *BrF3*′*H*; the LBGs *BrDFR1*, *BrANS1*, *BrUF3GT2*, *BrUF5GT*, *Br5MAT*, and *Brp-COUT*; the transport genes *BrGST1* and *BrGST2*; and the positive regulatory genes *BrMYB2* and *BrTT8* (Fig. 4e and Table S3). However, the genes in Group II.2 had lower expression in the outer leaves than in the inner leaves or were not significantly correlated with total anthocyanin content. These genes included the PMPGs *BrPAL2.2*, *BrPAL4.0*, *Br4CL2.1*, *Br4CL2.3*, and *Br4CL3.0*; the EBGs *BrCHS5*, *BrCHI1*, *BrCHI2*, *BrCHI3*, and *BrF3H1*; and the LBGs *BrDFR3*, *BrANS2*, *BrANS3*, *BrANS4*, *BrUF3GT1*, *BrUGT84A1.2*, and *BrUGT84A2.2*. The genes in Group II.3 displayed low correlations with total anthocyanin content or were expressed at low levels in purple head Chinese cabbage. These genes included the PMPGs *BrPAL3.2* and *Br4CL4.2*; the EBG *BrF3H2*, the LBGs *BrDFR2*, *BrSAT*, *BrUGT84A1.1*, and *BrUGT84A2.1*; the positive regulatory genes *BrMYB12.1*, *BrMYB111.1*, *BrMYB111.2*, *BrEGL3.1*, and *BrGL3*; and the negative regulatory genes *BrLBD38.2* and *BrLBD39.3*. These results showed that the increased upregulation of *BrMYB2* promoted a large accumulation of anthocyanins in purple head Chinese cabbage and in its purple trait donor at the head-formation stage. This was accompanied by high upregulated expression of the bHLH regulatory gene *BrTT8*; the PMPGs *BrPAL2.1*, *BrPAL2.3*, and *BrPAL3.1*; the EBGs *BrCHS1*, *BrCHS2*, *BrCHS3*, *BrCHS4*, *BrF3H3*, and *BrF3*′*H*; the LBGs *BrDFR1*, *BrANS1*, *BrUF3GT2*, *BrUF5GT*, *Br5MAT*, and *Brp-COUT*; and the transport genes *BrGST1* and *BrGST2*.

### Variations upstream of *BrMYB2* have no effects on the function of its promoter

**Fig. 5:**
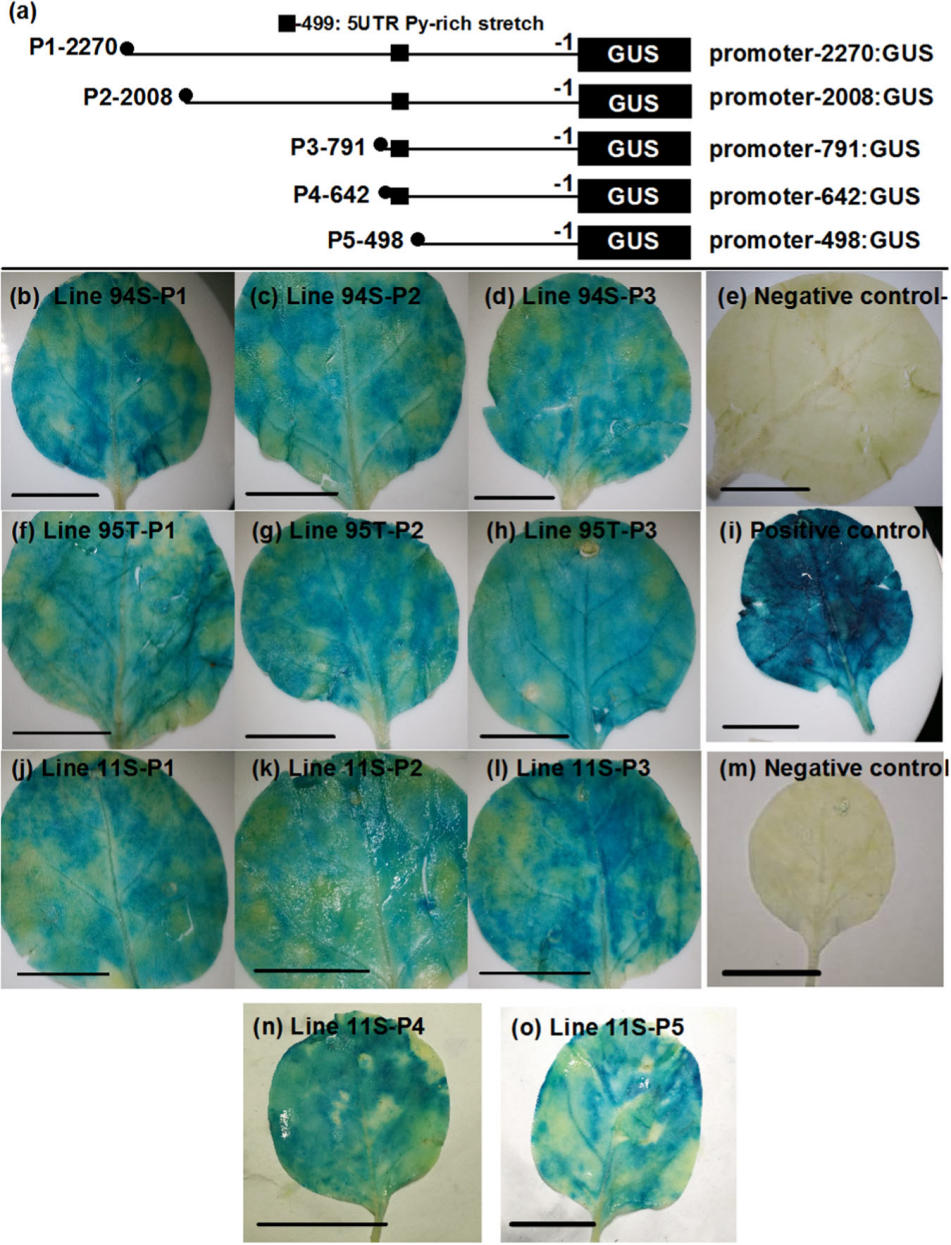
Histochemical dyeing assay of GUS in transformed tobacco leaves for analyzing *BrMYB2* promoter activity. **a***GUS* constructs containing different lengths of *BrMYB2* promoter sequences (based on 11S91). Schematic diagram of constructs utilized for transient transformation of tobacco ‘NC89’ leaves that were 6 cm in size (~28 DAS [days after sowing]). Tobacco leaves were infiltrated with different plasmids from bacteria at a concentration of OD_600_ = 0.530 and cultured for 72 h under a 12-h light/12-h dark photoperiod at 23 °C (125 mmol m^–2^ s^–1^). The leaves were imaged after GUS solution staining and alcohol decolonization. **b**, **f**, **j** Constructs with a 2270 bp long *BrMYB2* promoter (based on that of 11S91). **c**, **g**, **k** Constructs with a 2008 bp long *BrMYB2* promoter (based on that of 11S91). **d**, **h**, **l** Constructs with a 791 bp long *BrMYB2* promoter (based on that of 11S91). **n** Construct with 642 bp long *BrMYB2* promoter. **o** Construct with a 498 bp long *BrMYB2* promoter. **e** Negative control: noninfiltrated leaves. **i** Positive control: leaves infiltrated with the CaMV35S:*GUS* construct. **m** Negative control: leaves infiltrated with the *GUS* construct without CaMV35S promoter. The data as shown are representative of three independent repeats (*n* = 3). The scale bar is 2.5 cm

First, we speculated that differences existed in the *BrMYB2* promoter and questioned whether these variations might lead to different degrees of expression of *BrMYB2*. A CT repeat deletion was found upstream from the ATG translation start site of *BrMYB2* in 11S91, and four CT repeat deletions were found in the same site in 95T2-5 (Fig. S8). The CT repeat area was a 5′-UTR Py-rich stretch that served as a cis-acting element providing high transcription levels, according to the PlantCARE database (Fig. S7). Although different numbers of CT repeats were constructed and transiently transformed into tobacco leaves, samples treated with different constructs from three Chinese cabbage lines showed identical degrees of blue color (Fig. 5b–l). Moreover, activity analyses of the *BrMYB2* promoter, including a series of cis-acting element deletions (such as CAAT-boxes, TATA-boxes, and AS-2-boxes) from −791 to −2270 bp, revealed the same degree of color (Fig. 5b–l). In addition, *BrMYB2* promoter activity was not changed when the 5′-UTR Py-rich stretch region was deleted (Fig. 5o); the *BrMYB2* promoter activity was lower than the cauliflower mosaic virus 35S (CaMV35S) promoter activity, as the positive control had a deeper blue color (Fig. 5). These results indicated that variations upstream of *BrMYB2* may not cause loss of promoter activity and do not affect the expression of *BrMYB2*.

### Two SNPs do not lead to loss of function of *BrMYB2*

**Fig. 6:**
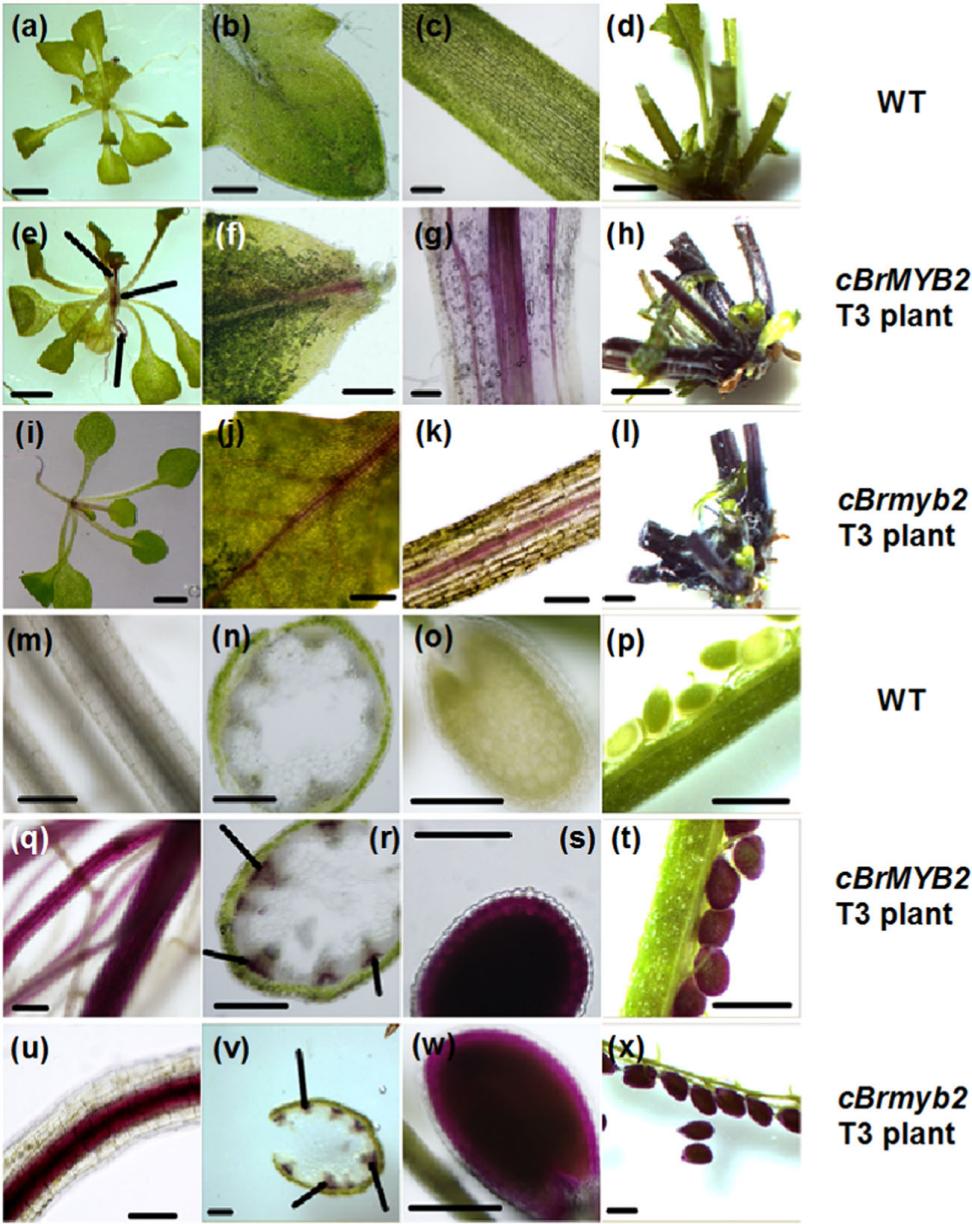
Phenotype comparisons of different tissues of c*BrMYB2* and *cBrmyb2* transgenic *Arabidopsis* under control of the CaMV35S. **a**–**d**, **m**–**p** WT *Arabidopsis*; **e**–**h**, **q**–**t** T_3_ CaMV35S:c*BrMYB2* lines; **i**–**l**, **u**–**x** T_3_ CaMV35S:c*Brmyb2* lines. **a**, **e**, **i** Twelve-day-old seedlings. **b**, **f**, **j** Inner leaves close to the growing point. **c**, **g**, **k** Rosette leaf petioles. **d**, **h**, **l** Rosette leaf stalk bases. **m**, **q**, **u** Roots. **n**, **r**, **v** Cross-sections of stalks. **o**, **s**, **w** Seeds at approximately 7 days after pollination. **p**, **t**, **x** Siliques at approximately 7 days after pollination. The scale bars are 200 μm

In addition to promoter variations, two SNPs in *cBrMYB2* were present and conserved between white-head lines and purple-head lines (Fig. S2c). To test whether these SNPs were responsible for the purple-head phenotype, different introductions of c*BrMYB* and *cBrmyb2* into *Arabidopsis* were performed using genetic transformation. A total of 62 T_1_ CaMV35S:*cBrMYB2* lines and 56 T_1_ CaMV35S:*cBrmyb2* lines were obtained after PCR detection (Fig. S9). Seven c*BrMYB2* lines (Line 02, Line 06, Line 14, Line 27, Line 32, Line 46, and Line 49) and four c*Brmyb2* lines (Line 01, Line 02, Line 05, and Line 06) with the best purple-color phenotype were identified and selected for generating T_3_ homozygous plants (Fig. S10). Compared with wild type (WT) *Arabidopsis*, both transgenic c*BrMYB2* and *cBrmyb2* lines showed distinct purple premature seeds and purple vascular bundles of the leaves, roots, and stalks (Fig. 6).

The expression of *BrMYB2* in Line 02, Line 14, Line 27, Line 32, Line 46, and Line 49 was 1.5-4 times higher than that in Line 06 (Fig. S11i); however, no *BrMYB2* expression was detected in WT *Arabidopsis* in either quantitative real-time PCR (qRT-PCR) or semi-qRT-PCR analyses. Accompanied by the high upregulation of *AtTT8*, EBGs such as *AtCHI*, *AtCHS*, *AtF3H*, and *AtF3*′*H* and LBGs such as *AtDFR*, *AtANS*, *AtUF3GT1*, *AtUF3GT2*, *AtUF5GT*, *At5MAT*, and *AtGST* showed higher expression in the transgenic lines than in the WT (Fig. S11j–x). *AtTT2*, *AtEGL3*, and *AtTTG1* presented relatively high expression in the transgenic lines (Fig. S11). These results indicated that both *cBrmyb2* and *cBrMYB2* can activate the anthocyanin biosynthesis pathway and that silent mutations caused by two SNPs in *cBrMYB2* cannot cause loss of function.

### The short intron 1 with a large deletion in *BrMYB2* promotes high transcription, which is responsible for the purple-head phenotype

**Fig. 7:**
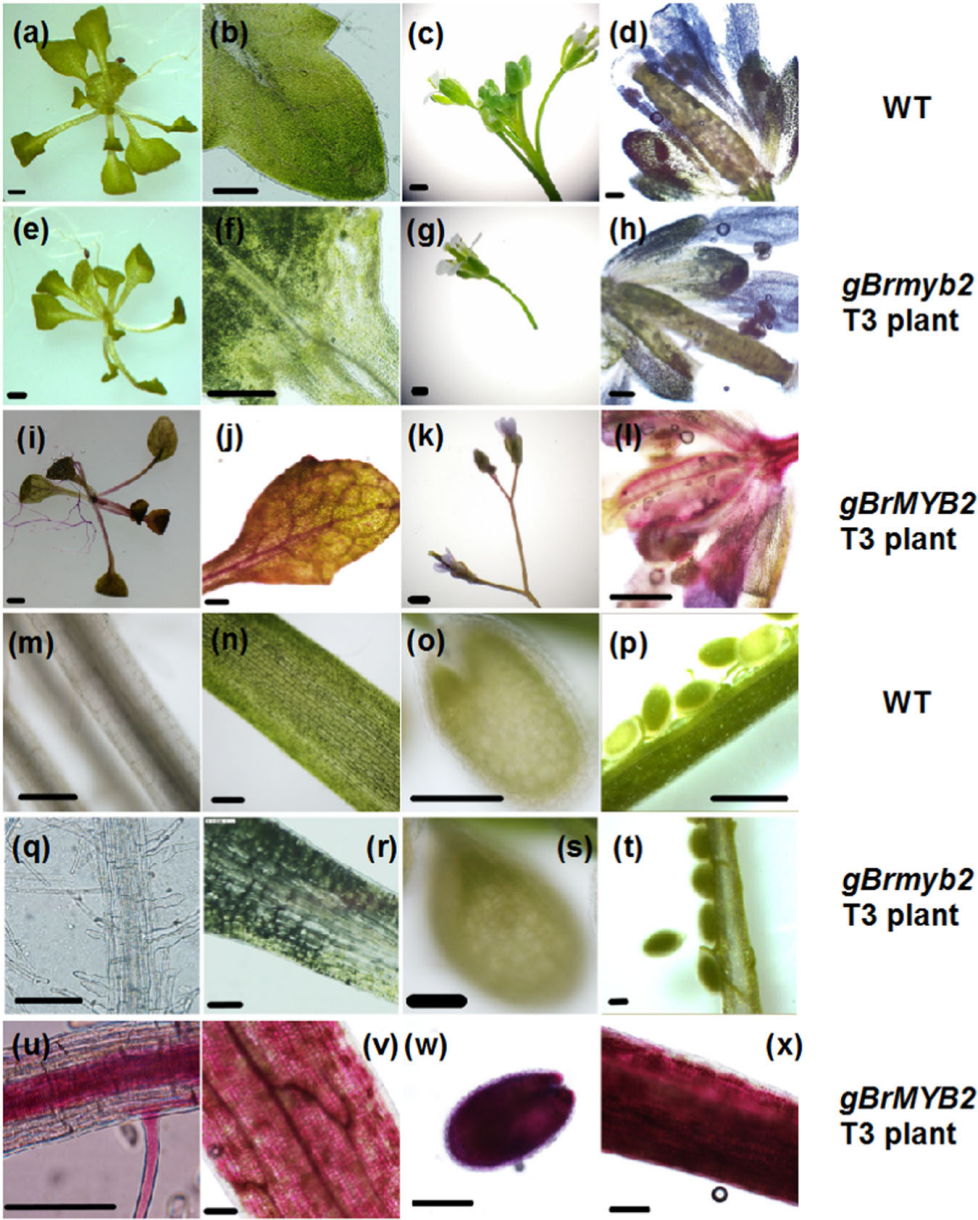
Phenotype comparisons of different tissues of transgenic g*BrMYB2* and g*Brmyb2 Arabidopsis* under the CaMV35S control. **a**–**d**, **m**–**p** WT *Arabidopsis*; **e**–**h**, **q**–**t** T_3_ CaMV35S:g*Brmyb2* lines; **i**–**l**, **u**–**x** T_3_ CaMV35S:g*BrMYB2* lines. **a**, **e**, **i** Twelve-day-old seedlings. **b**, **f**, **j** Inner leaves close to the growing point. **c**, **g**, **k** Inflorescences. **d**, **h**, **l** Flowers. **m**, **q**, **u** Roots. **n**, **r**, **v** Rosette leaf petioles. **o**, **s**, **w** Seeds at approximately 7 days after pollination. **p**, **t**, **x** Siliques at approximately 7 days after pollination. The scale bars are 200 μm

We questioned whether the different intron 1 of *BrMYB2* was responsible for the different phenotypes of anthocyanin accumulation. A total of 46 T_1_ CaMV35S:*gBrMYB2* lines and 59 T_1_ CaMV35S:*gBrmyb2* lines were obtained after PCR detection after *Arabidopsis* genetic transformation (Fig. S12). Ten g*BrMYB2* lines with the best purple coloration were selected for generating T_3_ homozygous plants; however, the phenotype of all the g*Brmyb2* lines was the same as that of WT *Arabidopsis* (Fig. S13). On the contrary, only the g*BrMYB2* lines showed a deeper purple color in the roots, flowers, siliques, rosette leaves, stalks, inflorescences, premature seeds, and seedlings older than 12 days (Fig. 7). Interestingly, the degrees of purple color of these tissues were much deeper than that of the tissues of the c*BrMYB2* and c*Brmyb2* lines (Figs. 6 and 7). Thus, only *gBrMYB2* with a short intron 1 promotes purple color formation and produces a purple phenotype, but *gBrmyb2* with a long intron 1 causes purple color deficiency and no purple phenotype.

**Fig. 8:**
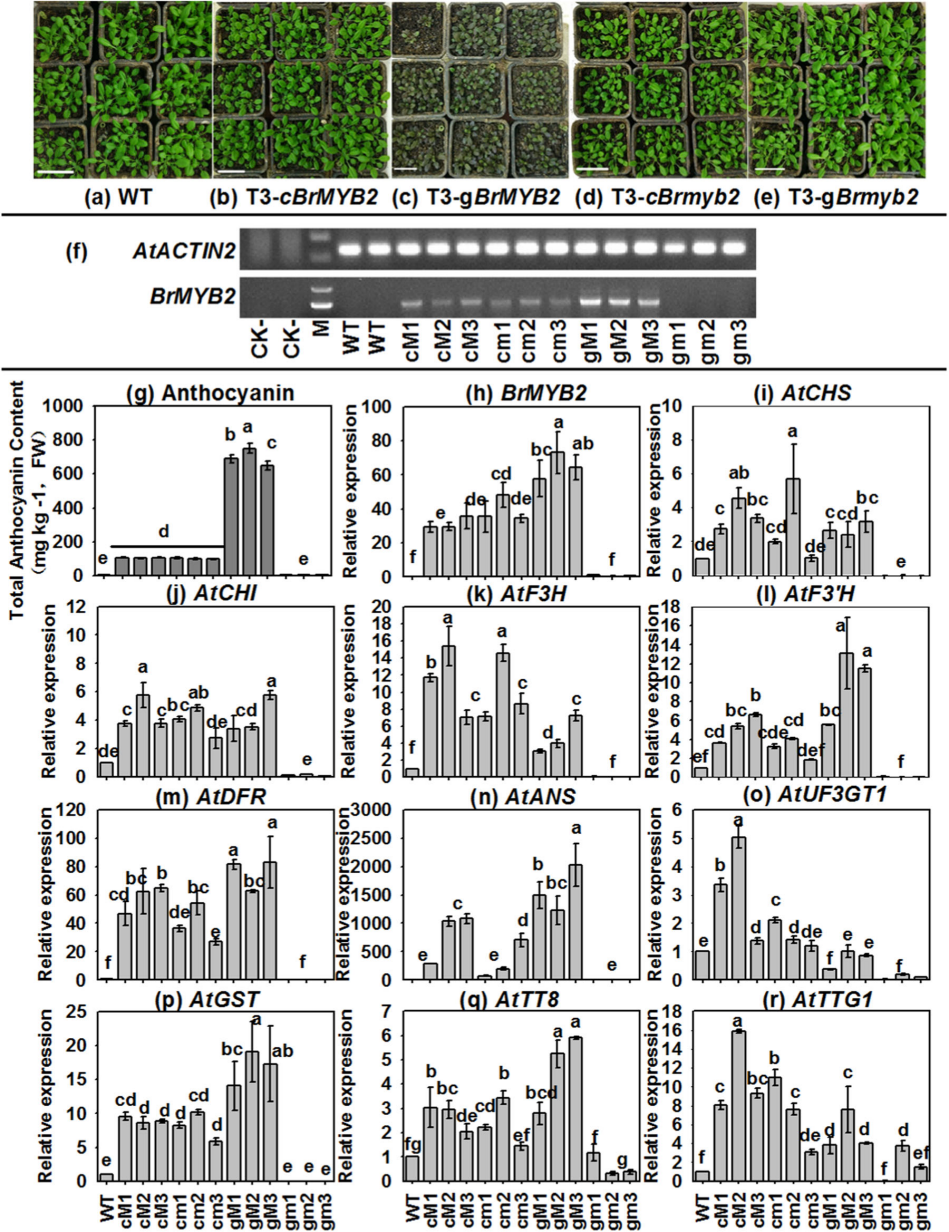
Total anthocyanin content and expression patterns of related ABGs in different transgenic *Arabidopsis* lines. **a**–**e** Thirty-day-old *Arabidopsis* seedlings. **a** WT *Arabidopsis*; **b** T_3_ CaMV35S:*cBrMYB2* lines; **c** T_3_ CaMV35S:*gBrMYB2* lines; **d** T_3_ CaMV35S:*cBrmyb2* lines; **e** T_3_ CaMV35S:*gBrmyb2* lines. **f** Semi-qRT-PCR analysis of full-length *BrMYB2*. **g** Total anthocyanin contents of samples. **h** qRT-PCR analysis of *BrMYB2*, and Line 01 of the T_3_*gBrmyb2* lines served as the control. **i**–**r** Expression patterns of ABGs in *Arabidopsis*, and the WT line served as the control. The values are presented as the means ± SDs (*n* = 3). The different letters above each column are significantly different at *p* < 0.05 according to Duncan’s test. The first ‘c’ and ‘g’ letters of the abscissa labels represent cDNA and gDNA, respectively; the second letters ‘M’ and ‘m’ represent *BrMYB2* and *Brmyb2*, respectively

Moreover, we measured the total anthocyanin content and related gene expression in all the transgenic plants. Both the g*Brmyb2* lines and WT *Arabidopsis* showed a nonpurple phenotype in their aboveground parts, which presented extremely low anthocyanin contents (Fig. 8a–g); the phenotypes of the aboveground parts of the *cBrMYB2* and *cBrmyb2* plants were identical and presented relatively high contents of anthocyanins (Fig. 8a–g). Notably, the whole leaves of *gBrMYB2* plants containing intron 1 had a deep purple color and the highest anthocyanin content, followed by those of the c*BrMYB2* and *cBrmyb2* lines, the *gBrmyb2* lines and WT *Arabidopsis* (Fig. 8a–g).

Furthermore, the expression patterns of *BrMYB2* and other ABGs were measured in transgenic lines. *BrMYB2* was expressed little in *gBrmyb2* plants but highly expressed in *cBrMYB2*, *cBrmyb2*, and *gBrMYB2* plants (Fig. 8f–h); the expression of *BrMYB2* was consistent with the total anthocyanin content, and the *gBrMYB2* lines had the highest expression of *BrMYB2*, followed by the c*BrMYB2* and *cBrmyb2* lines, the *gBrmyb2* lines and WT *Arabidopsis* (Fig. 8f–h). In addition, the reverse transcription results showed that *gBrMYB2* with short intron 1 had undergone correct intron splicing and produced the same transcript as that of the *cBrMYB2* lines; however, *Brmyb2* in the *gBrmyb2* lines with long intron 1 was expressed at a low level (Figs. 8f and S14). Moreover, ABGs in *gBrmyb2* lines were not activated, which indicated that the function of *gBrmyb2* was totally lost (Fig. 8i–r); the expression of ABGs in *cBrMYB2, cBrmyb2*, and *gBrMYB2* plants was also different. For example, the expression levels of the several EBGs (i.e., *AtCHI*, *AtCHS*, and *AtF3H*), the LBG *AtUG3GT1*, and the regulatory gene *AtTTG1* were similar in *cBrMYB2* and *cBrmyb2* plants, but their expression levels were lower in *gBrMYB2* plants with intron 1 (Fig. 8h–r). In contrast, the expression patterns of ABGs directly responsible for downstream anthocyanin biosynthesis were similar to those of *BrMYB2*, with higher expression in *gBrMYB2* plants with intron 1 than in *cBrMYB2* and *cBrmyb2* plants (Fig. 8h–q). These ABGs included the EBG *AtF3*′*H*; LBGs *AtDFR* and *AtANS*; transport gene *AtGST*; and regulatory gene *AtTT8* (Fig. 8h–q). Generally, *cBrMYB2* and *cBrmyb2* transgenic plants with moderate expression levels of *BrMYB2* presented higher upregulation of EBGs than did the *gBrMYB2* plants; *gBrMYB2* plants with the highest expression of *BrMYB2* mainly exhibited higher upregulation of the LBGs, *AtTT8*, and *AtGST*. Similar to the results of the mature Chinese cabbage heads, these ABGs may be directly controlled by *BrMYB2*. Hence, these results further illustrated that intron 1 of *BrMYB2* plays an important role in affecting anthocyanin biosynthesis and ABG regulation. Though *gBrmyb2* plants were under the control of the CaMV35S promoter, the long intron 1 with a large insertion in *gBrmyb2* still completely inhibited anthocyanin biosynthesis; the short intron 1 with a large deletion of *gBrMYB2* highly promoted anthocyanin production. As a result, *gBrmyb2* fully lost its function to activate anthocyanin biosynthesis.

## Discussion

### The new *BrPur* locus in *Brassica*

The purple trait of *Brassica* vegetables has become a popular research topic in recent years. Purple head Chinese cabbage not only has health benefits but also provides an important agronomic trait for germplasm resources. Interestingly, there are different loci that reportedly control the purple trait of *Brassica* species^19–22,28,33–35^. Recent investigations of an F_2_ population derived from the hybridization of zicaitai and common caixin (*B*. rapa L. ssp. *parachinensis*) demonstrated that two candidate genes were speculated to control anthocyanin accumulation in zicaitai, including the bHLH gene *BrEGL3.2* (positive) and the R3-MYB gene *BrMYBL2.1* (negative) located on chromosomes A09 and A07, respectively^19,20^. Similar to a rapid-cycling *B. rapa*, a recessive locus affording no anthocyanins was mapped to chromosome A09 without candidate gene prediction^36^. However, the mapping of an F_2_ population generated by green Chinese cabbage and purple bok choy showed that the dominant gene *Pur* was located at the end of chromosome A03^33^, and similar results were found in purple nonheading Chinese cabbage and in another *B. rapa* line^22,35^. In purple turnip (*B. rapa* cv. Iyo-hikabu), anthocyanin coloration was shown to be controlled by a single dominant gene on chromosome A07 based on a doubled-haploid population generated by crossing purple turnip and Chinese cabbage, although the responsible gene was not verified^37^. Another report speculated that *BrbHLH49* on chromosome A07 might positively regulate anthocyanin accumulation in zicaitai^21^. A purple Chinese cabbage created from the hybridization between Chinese cabbage and red leaf mustard revealed that the purple gene was translocated onto chromosome A02^28^. Though these purple *Brassica* plants might have similar phenotypes or anthocyanin-rich tissues, their different dominant *BrPur* loci mainly depend on the genetic background of the purple trait donors, at least in *Brassica* species. In our work, the first reported R2R3-MYB gene, *BrMYB2* (*BrPur*), located on chromosome A07 of *B. rapa*, was the key gene responsible for anthocyanin accumulation in heading leaves, stalks, and siliques of purple head Chinese cabbage; moreover, it is a novel, unpublished locus that differs from all reported loci in purple *Brassica* crops.

### Upregulation of *BrMYB2* promotes high expression of ABGs to generate purple traits

AtMYB113, AtMYB114, AtPAP1, and AtPAP2 activate anthocyanin biosynthesis in *Arabidopsis*, which have three orthologs in *B. rapa*, namely, Bra004162, Bra039763, and Bra001917, located on chromosomes A07, A02, and A03, respectively^8^. Recent studies of purple head Chinese cabbage revealed that total anthocyanin content is associated with high expression of only *BrMYB2* (Bra004162) among these three *BrMYB*s, accompanied by upregulation of *BrTT8* and a series of ABGs, including *BrF3’H*, *BrDFR*, *BrANS*, *BrUGT*s, and *BrGST*s^10,27^. Recent advances have shown that MYBs activate bHLHs and ABGs through strong interactions with bHLHs to promote anthocyanin biosynthesis in crop plants^38–40^. In purple cauliflower and red cabbage, *BoMYB2* is responsible for the purple trait by interacting with BoHLHs and related ABGs^15–17^, and homologous comparisons showed that *BrMYB2* is highly homologous with *BoMYB2* (Fig. 2c). High upregulation of *TT8*, *PAL*s, *F3*′*H*, *DFR*, *ANS*, *UGT*s, and *GST*s in both *Arabidopsis* transformants and Chinese cabbage strongly indicates that *BrMYB2* may cooperate with *AtTT8* or *BrTT8* to regulate anthocyanin biosynthesis in *Arabidopsis* and Chinese cabbage, respectively. In addition, *BrMYB2* also showed higher expression in young, tender purple tissues such as inner leaves or tissues close to the growing point (flowering buds and siliques); in addition, *BrTT8*, *BrF3*′*H*, *BrDFR*, and *BrANS* might function redundantly in proanthocyanidin biosynthesis and brown testa formation in Chinese cabbage, as shown in a previous study^31,32^.

### Expression of *BrMYB2* was not affected by mutations in its upstream promoter

Upregulation of *MYB*s has been considered to be the key factor in anthocyanin production in natural variants or artificially bred plants^4^, and the different transcription levels of these *MYB*s are usually caused by their promoters. For example, in apple, a rearrangement in the upstream regulatory region of *MdMYB10* is responsible for increased anthocyanin accumulation and for producing a striking phenotype involving red foliage and fruit flesh^41^. When the *PcMYB10* promoters were compared between red-skinned pear and green-skinned pear, a higher methylation level of the *PcMYB10* promoter caused lower expression of *PcMYB10*, and anthocyanin accumulation in the green-skinned pear was tightly associated with the methylation level of the *PcMYB10* promoter^12^. In addition, genetic variation in a harbinger DNA transposon insertion upstream of *BoMYB2* crucially affects the upregulation of *BoMYB2* to induce a purple phenotype of cauliflower^15^, whereas a retrotransposon-induced mutation in the 5′-flanking region of *VvmybA1* is associated with the loss of anthocyanins in white grape^42^. Recently, deletion in the upstream promoter of *BoMYBL2-1* at the 347 bp site resulted in purple cabbage^13^. The extremely close relationship among species of *Brassica* vegetables may provide compatibility between the MBW regulatory machinery and their binding sites in promoters^7^. Hence, the promoter activity of candidate genes is critical to the novel purple trait, and potential discrepancies may be distributed in cis-regulatory regions or even epigenetic marks^12,15^. However, unlike the above reports on purple color formation, our results appear to present a different mechanism responsible for the increased expression of *BrMYB2* during anthocyanin biosynthesis. Although variations in the 5′-UTR Py-rich stretch existed in the *BrMYB2* promoter, its activity remained unchanged. Hence, the differential expression of *BrMYB2* is not caused by promoter differences.

### Mutations in c*BrMYB2* cannot lose gene function

Natural gene mutations are not targeted, and sometimes these nonorientation mutations may produce different gene functions; the final results of mutations include missense mutations, synonymous mutations, silent mutations, frameshift mutations, and nonsense mutations^43^. For example, mutation of a single proline to leucine substitution in *BnRGA* led to a semidwarf mutant phenotype of *B. napus*^44^. A nonsynonymous SNP in the third exon of *Brnym1* produced an amino acid substitution in the highly conserved domain of magnesium dechelatase, which generated a stay-green phenotype of Chinese cabbage^45^. Similarly, variations in amino acids caused by SNPs in *Bror* did not affect its gene function, whereas an insertion in *Bror* caused early termination of translation and loss of gene function in orange head Chinese cabbage^46^. Coding regions of *BrMYB2* in 11S91 and 94S17 shared 99.19% sequence identity; two SNPs were found and were conserved. However, the gene functions of *cBrMYB2* and *cBrmyb2* were nearly identical because their transformants both produced a purple phenotype. Thus, silent mutations of base substitutions in *BrPur* are not the key factor in purple head formation.

### Intron 1 of *gBrMYB2* controls the transcription of *BrMYB2* and anthocyanin biosynthesis

Introns regulate gene expression in eukaryotes in various ways, such as positive, negative, and intron-mediated manners^47^. The regulatory sites of most important genes are not situated in the promoter but rather are located within introns, and genes regulated by introns are often highly expressed in most tissues^48^. Even when the promoters have been deleted, some introns located several hundred nucleotides downstream of the transcription start site still strongly stimulate mRNA accumulation^48^. For instance, a 90 bp sequence in intron 1 of *AtPAP1* is essential for sucrose responses^49^; intron 1 of *Ostua* is the key regulatory element of high accumulation of rice tubulin^50^; similarly, high expression of *OsTubA1* requires intron 1, and *OsTubA1* cannot be highly expressed when intron 1 exists in the untranslated region^51^. The *BnFAD2-C5* intron provides an enhancement function as a promoter, and the intron-mediated enhancement regions are mainly concentrated in the +631 bp to +1033 bp site^52^. Similarly, the 5′-UTR intron of *AtVTC1* acts as an enhancer for Vc biosynthesis in *Arabidopsis*^53^. A recent study further supported that introns promoted expression from all transcribed positions but had no effect when they were located upstream of the most 5′ transcription start site^48^. In our research, introduction of *gBrMYB2* with the short intron 1 and normal intron 2 showed a deep purple phenotype for all tissues of transgenic *Arabidopsis*, and their degrees of coloration were much deeper than those of *cBrMYB2* and *cBrmyb2* transgenic plants without related introns. In contrast, introduction of *gBrmyb2* with the long intron 1 and normal intron 2 resulted in phenotypes identical to those of WT *Arabidopsis*. Moreover, the short intron 1 enhanced the high transcription of *BrMYB2* in transgenic *gBrMYB2* lines.

In addition, introns have a repressor function for gene self-regulation. For example, a reverse repetitive sequence in intron 1 of the human collagen *α1(I)* gene inhibits its transcription only when the intron 3′ region is located downstream of *α1(I)*^54^. Mutant rice plants harboring a T-DNA insertion in intron 1 of *OsCAO1* were deficient in chlorophyll b and produced pale-green leaves^55^. Without changes in the GT-AG splice site at the both ends of intron 1, *Brmyb2* with a long fragment insertion in the middle of intron 1 was completely repressed, which led to failure to produce a purple phenotype. In addition, positive genetic methods and reverse genetic methods are usually used for gene functional verification; in addition to transgenic techniques, mutant complementary hybridization methods are usually and traditionally used in positive genetic research of crop species. In our subsequent research, a series of purple head Chinese cabbage lines were acquired by crossing purple head Chinese cabbage with other nonpurple head Chinese cabbage lines, followed by subsequent selection. The *BrPur*/*Brpur* loci that gave rise to purple color were conserved and tightly cosegragated with head color in these Chinese cabbage lines, and both Chinese cabbage lines produced functional transcripts of *BrMYB2/Brmyb2* (Figs. S1 and S2).

**Fig. 9:**
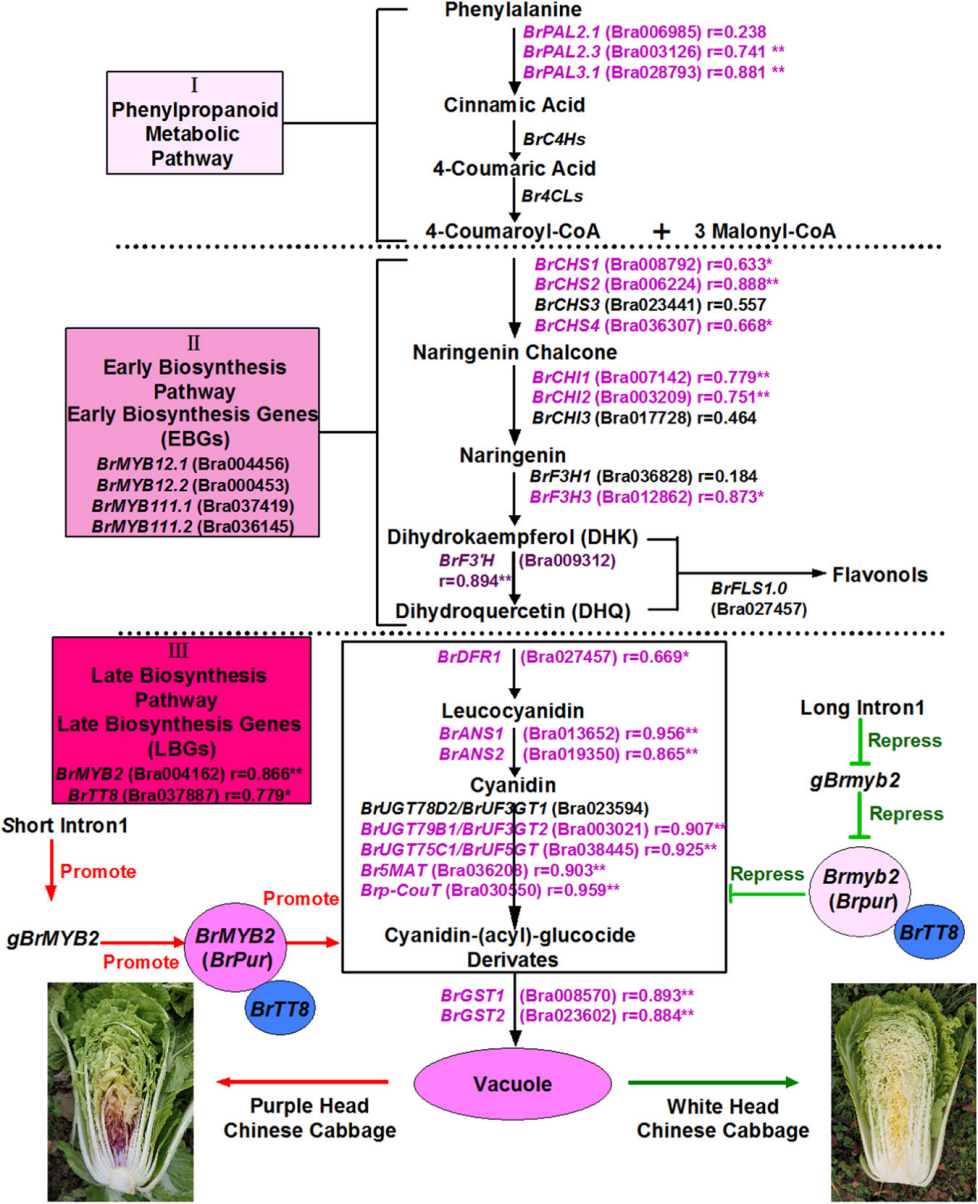
Anthocyanin biosynthesis pathway in purple head Chinese cabbage. *gBrMYB2* with short intron 1 controls anthocyanin biosynthesis and accumulation through coexpression of *BrTT8* and activation of a series of structural ABGs, such as *BrF3’H*, *BrDFR1*, *BrANS1*, *BrUGT*s, *BrAT*s, and *BrGST*s, resulting in a purple-head phenotype. Similarly, *gBrmyb2* of common white head Chinese cabbage with long intron 1 completely represses anthocyanin biosynthesis by restricting the expression of *Brmyb2* and ABGs, resulting in a nonpurple-head trait

In conclusion, *BrMYB2* is the key regulatory gene governing the purple-head trait in Chinese cabbage, and a short intron 1 of this novel gene promotes high expression of *BrMYB2* in purple head Chinese cabbage, which activates anthocyanin biosynthesis through substantial upregulation of *BrTT8*. This process is accompanied by upregulation of the PMPGs *BrPAL2.1*, *BrPAL2.3*, and *BrPAL3.1*; the EBGs *BrCHS1*, *BrCHS2*, *BrCHS3*, *BrCHS4*, *BrF3H3*, and *BrF3’H*; the LBGs *BrDFR1*, *BrANS1*, *BrUF3GT2*, *BrUF5GT*, *Br5MAT*, and *Brp-COUT*; and the transport genes *BrGST1* and *BrGST2* (Fig. 9). In contrast, the large intron 1 of *Brmyb2* completely inhibits its transcription in white head Chinese cabbage, resulting in no activation of the anthocyanin biosynthesis pathway. These findings provide new evidence to understand the mechanism of anthocyanin biosynthesis and lay a good foundation for purple germplasm use in *Brassica* crops. In the future, we will focus on the regulation of *BrMYB2* together with interactions of other anthocyanin biosynthesis factors in purple head Chinese cabbage.

## Materials and methods

### Plant materials and growth conditions

The inbred line 94S17 with white heads and brown testa served as the female parent (Fig. 1a), and the flowering Chinese cabbage 95T2-5 inbred line served as the male parent (purple trait donor, Fig. 1b), which has deep purple phenotype and brown testa. Chinese cabbage 11S91 with a purple head (Fig. 1c) was bred from F_1_ plants of 94S17 and 95T2-5 with continuous self-crossing for ten generations from select individual plants. An F_2_ population of head Chinese cabbage generated by crossing 11S91 and 14S162 (Fig. 1d, a yellow head Chinese cabbage served as the female parent) was used for fine mapping (Fig. 1e). In addition, different phenotypes of Chinese cabbage containing three white-head lines (15S1080, 15S1084, and 13S1147; Fig. S1b–d), an orange-head line (13S93, Fig. S1e), and four purple-head lines (14S838, Z33, Z190, and Z240; Fig. S1f–i) were used for the variation analysis of candidate genes. Z33, Z190, and Z240 were F_2_ purple head Chinese cabbage lines bred from a 14S838 female parent and 15S1080 male parent. All the materials were grown outdoors in autumn in Yangling, Shaanxi Province, China. The head color of individual F_2_ plants was visually determined at the mature-head stage. In addition, samples were harvested at the mature-head stage (about 4 months after sowing), frozen in liquid nitrogen, and immediately stored in a −80 °C freezer (Sanyo, Japan). Each sample was analyzed in triplicate, and three biological replicates were tested.

The WT *Arabidopsis* Columbia and tobacco line NC89 were used for genetic transformation. *Arabidopsis* cultivation was performed as previously described, with modifications^56^. Briefly, seeds were surface sterilized using 75% alcohol and disinfection solution (consisting of aqueous sodium hypochlorite solution and sterile distilled water at a final volumetric ratio of 1:10), washed using sterile distilled water and sown on Murashige and Skoog (MS) media (2% sucrose, 0.8% agar, and a pH of ~5.5). To unify germination and break dormancy, the seed plates were incubated for 2.5 days in the dark at 4 °C and then transferred into a culture room under a 12-h light/12-h dark photoperiod (125 mmol m^–2^ s^–1^) at 23 °C for 12 days. Similarly, tobacco and Chinese cabbage seeds were planted directly after soaking for 24 h. Twelve-day-old *Arabidopsis* seedlings, tobacco seedlings, and Chinese cabbage seedlings (cultivated for only 10 DAS, after which they were transplanted to the farm) were grown in a culture room in black plastic bowls (10 × 10 cm) containing peat, vermiculite, and perlite at a volumetric ratio of 3:1:1. The photoperiod was changed to 16-h light/8-h dark for *Arabidopsis* seed propagation during the seed-formation stage.

### DNA and RNA extraction, gene mapping, and gene expression

DNA was extracted by the CTAB assay as described previously^57^. Markers were developed based on the *B. rapa* genome (BRAD Database) around the *BrPur* locus according to previous research^29^. PCR amplification, gel electrophoresis, and genetic map construction were performed as previously reported^29,57^. Total RNA extraction, cDNA synthesis, and qRT-PCR via an IQ5 optical system (Bio-Rad, USA) were performed following a previously reported protocol^27^. All the qRT-PCR data were normalized using the cycle threshold value corresponding to *BrEF-1α* in *B. rapa* and *AtActin2* in *Arabidopsis*. The gene-specific primers used are listed in Table S1 and were used in a recent report^10^; the primers were confirmed by melting curve analysis for specific amplification. The relative expression of target genes was calculated using the 2^−ΔΔCT^ method by IQ5 software (Bio-Rad, USA). All reactions were conducted for three replicates.

### Sequence isolation, plasmid construction, and plant genetic transformation

The CDS and gDNA of the candidate gene were cloned using gene-specific primers (listed in Table S1) and then transferred into a pVBG2307 binary vector. The constructed vectors were transferred into *Agrobacterium tumefaciens* strain GV3101 using the freeze-thaw method and then transformed into WT *Arabidopsis* using the floral-dip method^56^. T_2_ progeny with the best purple coloration were selected from independent lines, and T_3_ homozygous lines were generated for subsequent research.

To construct promoter:*GUS* vectors, several fragments containing the promoter region of the candidate gene with HindIII and BamI restriction enzyme cutting sites were cloned into a pBI121 vector. The original pBI121 vector with the CaMV35S promoter was treated as the positive control, and the vector without the CaMV35S promoter was treated as the negative control. The primers used in these experiments are listed in Table S1. In transient genetic transformation, different GV3101 cell lines harboring unique vectors were transferred into tobacco line NC89 using the injection infiltration method, with slight modifications^58^. The injection concentration of GV3101 cells (OD_600_) was set to 0.530 using an UV-vis spectrophotometer (Thermo Fisher Scientific, USA).

PrimeSTAR Max mix (Takara, Japan) was used in the cloning process to reduce the mismatch rate during the PCR amplification process. The reaction volume and the three-step PCR amplification procedure were performed following the instructions of the mixture. All constructs were verified by sequencing (Auguct, China) for at least three technical repeats.

### Sequence analysis of the candidate gene and its promoter

Gene structure analysis of exons and introns was performed using Gene Structure Display Server 2.0 (http://gsds.cbi.pku.edu.cn/). The conserved domains were predicted by the SMART tool (http://smart.embl-heidelberg.de/). DNA and protein sequence alignment analyses were conducted by the multiple sequence alignment module of DNAMAN software (Lynnon BioSoft, Canada). A phylogenetic tree was constructed with MEGA 5.1 by the neighbor-joining method with 1000 bootstrap values. Promoter functional prediction was performed by tools associated with the PlantCARE database (http://bioinformatics.psb.ugent.be/webtools/plantcare/html/).

### Total anthocyanin analysis

Determination of total anthocyanin content was performed using an UV–vis spectroscopy method as previously described^10^, and data were calculated using a previous equation^59^. Approximately 1.0 g of crushed fresh samples were used in each extraction, and the results were expressed as the average of three biological replicates.

### Microscopy analysis

Different tissues were prepared for histological observations by manual sectioning as previously described^60^ and investigated using a fluorescence microscope (Olympus, Japan) at an appropriate magnification under the bright field.

### Statistical analysis

Cluster analysis of gene expression patterns in head tissues was performed using a two-way hierarchical clustering methodology via PermutMatrix software^61^, and Pearson distance and Ward’s method were applied for aggregation. One-way analysis of variance was conducted using SPSS 13.0 (Chicago, USA), and Duncan’s multiple-range test was performed on data at the 0.05 confidence level. Pearson correlation coefficients were calculated via two-tailed tests using bivariate analysis.

## Supplementary information

supplementary material files
