# Supplementary material for: The novel gene BrMYB2, located on chromosome A07, with a short intron 1 controls the purple-head trait of Chinese cabbage (Brassica rapa L.)
Source: Hortic Res. 2020 Jul 1;7:97. doi: 10.1038/s41438-020-0319-z (PMC7326913; doi:10.1038/s41438-020-0319-z)
Supplement: Supplementary file 1 — supplementary material files [file 41438_2020_319_MOESM1_ESM.pdf]

Supplementary Information for

The novel gene *BrMYB2*, located on chromosome A07, with a short intron 1 controls the purple-head trait of Chinese cabbage (*Brassica rapa* L.)

Author: Qiong He [1, 2], Junqing Wu [1], Yihua Xue [1], Wenbin Zhao [1], Ru Li [1], Lugang Zhang [1, 3*]

[1]State Key Laboratory of Crop Stress Biology for Arid Areas, College of Horticulture, Northwest A&F University, 3 Taicheng Road, Yangling 712100, Shaanxi, People's Republic of China.

[2]College of Life Sciences, Northwest A&F University, 3 Taicheng Road, Yangling 712100, Shaanxi, People's Republic of China.

[3]State Key Laboratory of Vegetable Germplasm Innovation, Tianjin, People's Republic of China.

*Corresponding Author: Lugang Zhang

Contact e-mail of corresponding author: lugangzh@163.com

This PDF file includes:

Supplementary text

Figures S1 to S14

Tables S1 to S3

Figure S1 Electrophoresis results of c*BrMYB2* and different phenotypes of Chinese cabbage. **a** Electrophoresis results of *cBrMYB2* from purple head Chinese cabbage '11S91', and its female parent '94S17' and male parent '95T2-5'; **b-d** different white head Chinese cabbages; **e** orange head Chinese cabbage; **f-i** different purple head Chinese cabbages; **j** electrophoresis results of *cBrMYB2* from different phenotypes of Chinese cabbage. M1: DL2000 Marker.

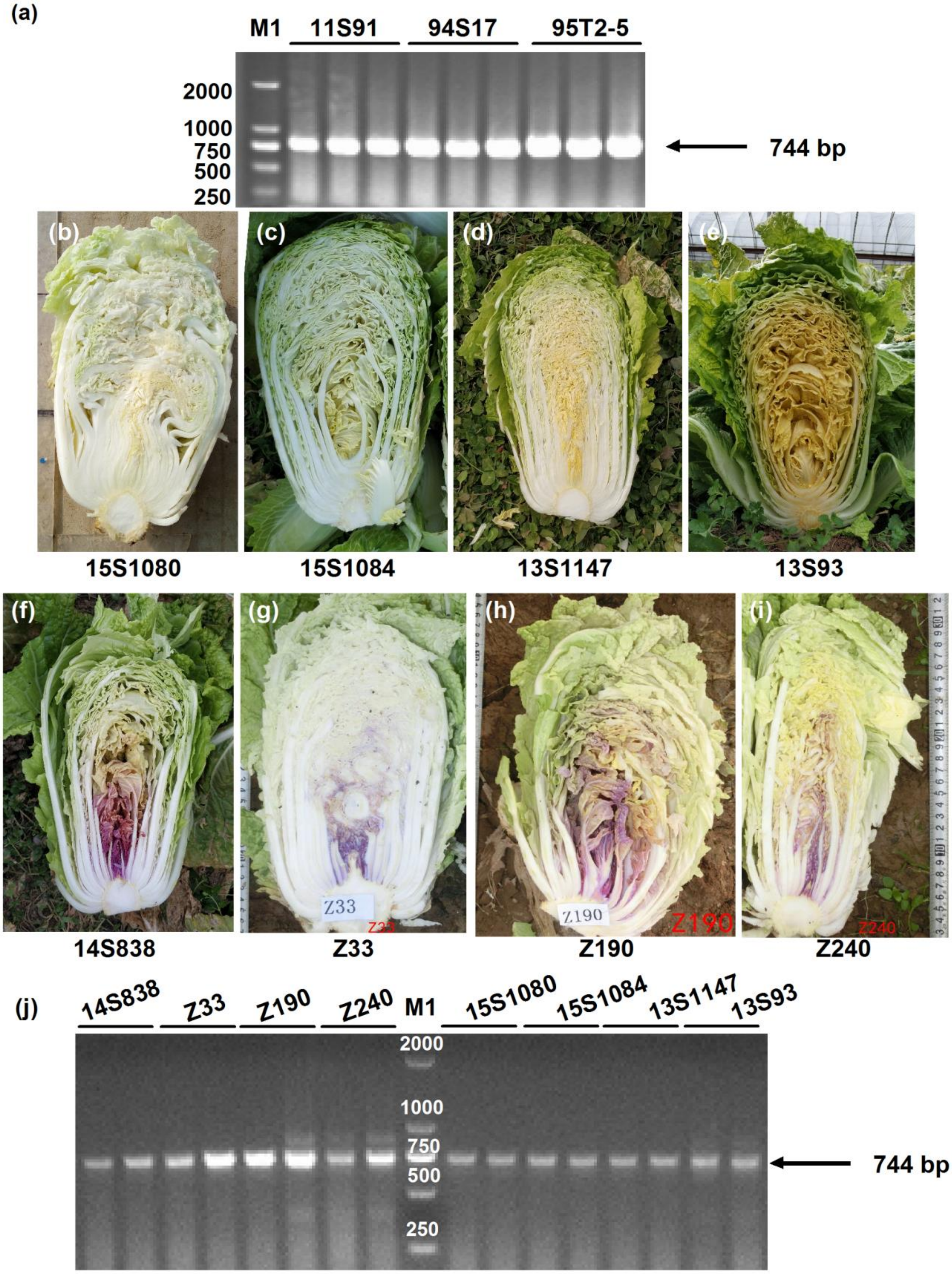

Figure S2 Multiple sequence alignment analysis of *BrMYBs* in *Brassica rapa*.

a Multiple sequence alignment analysis of c*BrMYB2* among purple head Chinese cabbage '11S91' and its parents '94S17' and '95T2-5'. '95T2-5' and '11S91' had consensus coding sequence (CDS). The *cBrMYB2* of 'Chifu' was downloaded from BRAD database.

```
CHIFU-cBrMYB2    ATGGAGGGTTCGTCCCAAGGGTTGAAAAAAGGTGCATGGACTGCTGAAGAAGATAATCTCTTGAGGCAATGCATTGATAA   80
94S17-cBrmyb2    ATGGAGGGTTCGTCCCAAGGGTTGAAAAAAGGTGCATGGACTGCTGAAGAAGATAATCTCTTGAGGCAATGCATTGATAA   80
11S91-cBrMYB2    ATGGAGGGTTCGTCCCAAGGGTTGAAAAAAGGTGCATGGACTGCTGAAGAAGATAATCTCTTGAGGCAATGCATTGATAA   80
95T2-5-cBrMYB2   ATGGAGGGTTCGTCCCAAGGGTTGAAAAAAGGTGCATGGACTGCTGAAGAAGATAATCTCTTGAGGCAATGCATTGATAA   80
Consensus        atggagggttcgtcccaagggttgaaaaaaggtgcatggactgctgaagaagataatctcttgaggcaatgcattgataa

CHIFU-cBrMYB2    GTATGGAGAAGGGAAATGGCACCAAGTTCCTTTAAGAGCTGGTCTAAATCGGTGCAGGAAGAGTTGTAGACTAAGATGGT  160
94S17-cBrmyb2    GTATGGAGAAGGGAAATGGCACCAAGTTCCTTTAAGAGCTGGTCTAAATCGGTGCAGGAAGAGTTGTAGACTAAGATGGT  160
11S91-cBrMYB2    GTATGGAGAAGGGAAATGGCACCAAGTTCCTTTAAGAGCTGGTCTAAATCGGTGCAGGAAGAGTTGTAGACTAAGATGGT  160
95T2-5-cBrMYB2   GTATGGAGAAGGGAAATGGCACCAAGTTCCTTTAAGAGCTGGTCTAAATCGGTGCAGGAAGAGTTGTAGACTAAGATGGT  160
Consensus        gtatggagaagggaaatggcaccaagttcctttaagagctggtctaaatcggtgcaggaagagttgtagactaagatggt

CHIFU-cBrMYB2    TGAACTATTTGAAGCCAAGTATCAAGAGAGGAAAACTCAACTCCGATGAAGTTGATCTTCTTATGCGCCTTCATAAGCTT  240
94S17-cBrmyb2    TGAACTATTTGAAGCCAAGTATCAAGAGAGGAAAACTCAACTCCGATGAAGTTGATCTTCTTATGCGCCTTCATAAGCTT  240
11S91-cBrMYB2    TGAACTATTTGAAGCCAAGTATCAAGAGAGGAAAACTCAACTCCGATGAAGTTGATCTTCTTATTCGCCTTCATAAGCTT  240
95T2-5-cBrMYB2   TGAACTATTTGAAGCCAAGTATCAAGAGAGGAAAACTCAACTCCGATGAAGTTGATCTTCTTATTCGCCTTCATAAGCTT  240
Consensus        tgaactatttgaagccaagtatcaagagaggaaaactcaactccgatgaagttgatcttcttat cgccttcataagctt

CHIFU-cBrMYB2    TTAGGAAACAGGTGGTCTTTAATTGCTGGTAGATTACCCGGTCGGACCGCCAATGACGTCAAAAATTACTGGAACACCCA  320
94S17-cBrmyb2    TTAGGAAACAGGTGGTCTTTAATTGCTGGTAGATTACCCGGTCGGACCGCCAATGACGTCAAAAATTACTGGAACACCCA  320
11S91-cBrMYB2    TTAGGAAACAGGTGGTCTTTAATTGCTGGTAGATTACCCGGTCGGACCGCCAATGACGTCAAAAATTACTGGAACACCCA  320
95T2-5-cBrMYB2   TTAGGAAACAGGTGGTCTTTAATTGCTGGTAGATTACCCGGTCGGACCGCCAATGACGTCAAAAATTACTGGAACACCCA  320
Consensus        ttaggaaacaggtggtctttaattgctggtagattacccggtcggaccgccaatgacgtcaaaaattactggaacaccca

CHIFU-cBrMYB2    TTTGAGTAAGAAACATGAACCGGGTTGTAAGACCCAGATGAAAAAGAGAAACATTCCTTGCTCTTATACCACACCAGCCC  400
94S17-cBrmyb2    TTTGAGTAAGAAACATGAACCGGGTTGTAAGACCCAGATGAAAAAGAGAAACATTCCTTGCTCTTATACCACACCAGCCC  400
11S91-cBrMYB2    TTTGAGTAAGAAACATGAACCGGGTTGTAAGACCCAGATGAAAAAGAGAAACATTCCTTGCTCTTATACCACACCAGCCC  400
95T2-5-cBrMYB2   TTTGAGTAAGAAACATGAACCGGGTTGTAAGACCCAGATGAAAAAGAGAAACATTCCTTGCTCTTATACCACACCAGCCC  400
Consensus        tttgagtaagaaacatgaaccgggttgtaagacccagatgaaaaagagaaacattccttgctcttataccacaccagccc

CHIFU-cBrMYB2    AAAAAATCGACGTTTTCAAACCTCGACCTCGATCCTTCACCGTTAACAGCGGCTGCAGCCATAATAATGGCATGCCAGAA  480
94S17-cBrmyb2    AAAAAATCGACGTTTTCAAACCTCGACCTCGATCCTTCACCGTTAACAGCGGCTGCAGCCATAATAATGGCATGCCAGAA  480
11S91-cBrMYB2    AAAAAATCGACGTTTTCAAACCTCGACCTCGATCCTTCACCGTTAACAGCGGCTGCAGCCATAATAATGGCATGCCAGAA  480
95T2-5-cBrMYB2   AAAAAATCGACGTTTTCAAACCTCGACCTCGATCCTTCACCGTTAACAGCGGCTGCAGCCATAATAATGGCATGCCAGAA  480
Consensus        aaaaaatcgacgttttcaaacctcgacctcgatccttcaccgttaacagcggctgcagccataataatggcatgccagaa

CHIFU-cBrMYB2    GCTGACATTGTTCCTCTATGCCTTGGACACAACGATACTAATAATGTTTCTGAAAATATAATCACATGTAACAAAGATGA  560
94S17-cBrmyb2    GCTGACATTGTTCCTCTATGCCTTGGACACAACGATACTAATAATGTTTCTGAAAATATAATCACATGTAACAAAGATGA  560
11S91-cBrMYB2    GCTGGCATTGTTCCTCTATGCCTTGGACACAACGATACTAATAATGTTTCTGAAAATATAATCACATGTAACAAAGATGA  560
95T2-5-cBrMYB2   GCTGGCATTGTTCCTCTATGCCTTGGACACAACGATACTAATAATGTTTCTGAAAATATAATCACATGTAACAAAGATGA  560
Consensus        gctg cattgttcctctatgccttggacacaacgatactaataatgtttctgaaaatataatcacatgtaacaaagatga

CHIFU-cBrMYB2    TGATAAATCTGAGCTTGTTAGTCATTTAATGGATGGTCAGAATAGGTGGTGGGAAAGTTTGCTAGATGAGAGCCAAGATC  640
94S17-cBrmyb2    TGATAAATCTGAGCTTGTTAGTCATTTAATGGATGGTCAGAATAGGTGGTGGGAAAGTTTGCTAGATGAGAGCCAAGATC  640
11S91-cBrMYB2    TGATAAATCTGAGCTTGTTAGTCATTTAATGGATGGTCAGAATAGGTGGTGGGAAAGTTTGCTAGATGAGAGCCAAGATC  640
95T2-5-cBrMYB2   TGATAAATCTGAGCTTGTTAGTCATTTAATGGATGGTCAGAATAGGTGGTGGGAAAGTTTGCTAGATGAGAGCCAAGATC  640
Consensus        tgataaatctgagcttgttagtcatttaatggatggtcagaataggtggtgggaaagtttgctagatgagagccaagatc

CHIFU-cBrMYB2    CAGCTGCGCTCTTTCCAGAAACTACAGCAATAAAAAAGGGCGCAACCTCCGCGTTTGACGTTGAGCAACTTTGGAGCCTG  720
94S17-cBrmyb2    CAGCTGCGCTCTTTCCAGAAACTACAGCAATAAAAAAGGGCGCAACCTCCGCGTTTGACGTTGAGCAACTTTGGAGCCTG  720
11S91-cBrMYB2    CAGCTGCGCTCTTTCCAGAAACTACAGCAATAAAAAAGGGCGCAACCTCCGCGTTTGACGTTGAGCAACTTTGGAGCCTG  720
95T2-5-cBrMYB2   CAGCTGCGCTCTTTCCAGAAACTACAGCAATAAAAAAGGGCGCAACCTCCGCGTTTGACGTTGAGCAACTTTGGAGCCTG  720
Consensus        cagctgcgctctttccagaaactacagcaataaaaaagggcgcaacctccgcgtttgacgttgagcaactttggagcctg

CHIFU-cBrMYB2    TTGGATGGAGAAACTGGAACTTG                                                          743
94S17-cBrmyb2    TTGGATGGAGAAACTGGAACTTG                                                          743
11S91-cBrMYB2    TTGGATGGAGAAACTGGAACTTG                                                          743
95T2-5-cBrMYB2   TTGGATGGAGAAACTGGAACTTG                                                          743
Consensus        ttggatggagaaactggaacttg
```

b Multiple sequence alignment analysis of c*BrMYBs* in *B. rapa*.

```
CHIFU-cBrMYB2      ATGGAGGGTTCGTCCCAAGGGTTGAAAAAAGGTGCATGGACTGCTGAAGAAGATAATCTCTTGAGGCAATGCATTGATAA   80
94S17-cBrmyb2      ATGGAGGGTTCGTCCCAAGGGTTGAAAAAAGGTGCATGGACTGCTGAAGAAGATAATCTCTTGAGGCAATGCATTGATAA   80
11S91-cBrMYB2      ATGGAGGGTTCGTCCCAAGGGTTGAAAAAAGGTGCATGGACTGCTGAAGAAGATAATCTCTTGAGGCAATGCATTGATAA   80
95T2-5-cBrMYB2     ATGGAGGGTTCGTCCCAAGGGTTGAAAAAAGGTGCATGGACTGCTGAAGAAGATAATCTCTTGAGGCAATGCATTGATAA   80
CHIFU-cBrMYB1      ATGGAGGATTCGTCCAAAGGGTTGACAAAAGGTGCATGGACCGCTGAAGAAGACAGTCTCTTGAGGCGATGCATTGATAA   80
CHIFU-cBrPAP1      ATGGAGGGTTCGCCAAAAGGGTTGAGAAAAGGTGCATGGACTGCTGAAGAAGATAGTCTCTTAAGGCAATGCATTGATAA   80
Consensus          atggagg ttcg c  aagggttga aaaaggtgcatggac gctgaagaaga a tctctt aggc atgcattgataa

CHIFU-cBrMYB2      GTATGGAGAAGGGAAATGGCACCAAGTTCCTTTAAGAGCTGGTCTAAATCGGTGCAGGAAGAGTTGTAGACTAAGATGGT  160
94S17-cBrmyb2      GTATGGAGAAGGGAAATGGCACCAAGTTCCTTTAAGAGCTGGTCTAAATCGGTGCAGGAAGAGTTGTAGACTAAGATGGT  160
11S91-cBrMYB2      GTATGGAGAAGGGAAATGGCACCAAGTTCCTTTAAGAGCTGGTCTAAATCGGTGCAGGAAGAGTTGTAGACTAAGATGGT  160
95T2-5-cBrMYB2     GTATGGAGAAGGGAAATGGCACCAAGTTCCTTTAAGAGCTGGTCTAAATCGGTGCAGGAAGAGTTGTAGACTAAGATGGT  160
CHIFU-cBrMYB1      GTATGGAGAAGGCAAATGGCATCAAGTTCCTTTAAGAGCTGGGCTTAATAGGTGTAGGAAGAGTTGTAGACTAAGATGGC  160
CHIFU-cBrPAP1      GTATGGAGAAGGAAAATGGCACCAAGTTCCTCTGCGAGCAGGGCTAAATCGGTGCAGGAAGAGCTGTAGACTAAGATGGT  160
Consensus          gtatggagaagg aaatggca caagttcct t  gagc gg ct aat ggtg aggaagag tgtagactaagatgg

CHIFU-cBrMYB2      TGAACTATTTGAAGCCAAGTATCAAGAGAGGAAAACTCAACTCCGATGAAGTTGATCTTCTTATGCGCCTTCATAAGCTT  240
94S17-cBrmyb2      TGAACTATTTGAAGCCAAGTATCAAGAGAGGAAAACTCAACTCCGATGAAGTTGATCTTCTTATGCGCCTTCATAAGCTT  240
11S91-cBrMYB2      TGAACTATTTGAAGCCAAGTATCAAGAGAGGAAAACTCAACTCCGATGAAGTTGATCTTCTTATTCGCCTTCATAAGCTT  240
95T2-5-cBrMYB2     TGAACTATTTGAAGCCAAGTATCAAGAGAGGAAAACTCAACTCCGATGAAGTTGATCTTCTTATTCGCCTTCATAAGCTT  240
CHIFU-cBrMYB1      TGAACTATTTGAAGCCAAATATCAAGAGAGGAAAACTTAGCTCTGATGAAGTTGATCTTCTTCTCCGTCTTCATAAGCTT  240
CHIFU-cBrPAP1      TGAACTATTTGAAGCCGAGTATCAAGAAAGGAAAACTTAGCTCTGATGAAGTTGATCTTCTTCTTCGCCTTCATAGGCTT  240
Consensus          tgaactatttgaagcc a tatcaaga aggaaaact a ctc gatgaagttgatcttctt t cg cttcata gctt

CHIFU-cBrMYB2      TTAGGAAACAGGTGGTCTTTAATTGCTGGTAGATTACCCGGTCGGACCGCCAATGACGTCAAAAATTACTGGAACACCCA  320
94S17-cBrmyb2      TTAGGAAACAGGTGGTCTTTAATTGCTGGTAGATTACCCGGTCGGACCGCCAATGACGTCAAAAATTACTGGAACACCCA  320
11S91-cBrMYB2      TTAGGAAACAGGTGGTCTTTAATTGCTGGTAGATTACCCGGTCGGACCGCCAATGACGTCAAAAATTACTGGAACACCCA  320
95T2-5-cBrMYB2     TTAGGAAACAGGTGGTCTTTAATTGCTGGTAGATTACCCGGTCGGACCGCCAATGACGTCAAAAATTACTGGAACACCCA  320
CHIFU-cBrMYB1      TTAGGAAACAGGTGGTCTTTAATTGCTGGTAGACTACCCGGTCGGACCGCTAATGATATCAAGAATTACTGGAACACCCA  320
CHIFU-cBrPAP1      CTAGGAAACAGGTGGTCTTTAATTGCTGGTAGATTACCCGGTAGGACCGCTAATGATGTCAAGAACTACTGGAACACCCA  320
Consensus           taggaaacaggtggtctttaattgctggtaga tacccggt ggaccgc aatga  tcaa aa tactggaacaccca

CHIFU-cBrMYB2      TTTGAGTAAGAAACATGAACCGGGTTGTAAGACCCAGATGAAAAAGAGAAACATTCCTTGCTCTTATACCACACCAGCCC  400
94S17-cBrmyb2      TTTGAGTAAGAAACATGAACCGGGTTGTAAGACCCAGATGAAAAAGAGAAACATTCCTTGCTCTTATACCACACCAGCCC  400
11S91-cBrMYB2      TTTGAGTAAGAAACATGAACCGGGTTGTAAGACCCAGATGAAAAAGAGAAACATTCCTTGCTCTTATACCACACCAGCCC  400
95T2-5-cBrMYB2     TTTGAGTAAGAAACATGAACCGGGTTGTAAGACCCAGATGAAAAAGAGAAACATTCCTTGCTCTTATACCACACCAGCCC  400
CHIFU-cBrMYB1      TCTGAGCAAGAAACATGAACCATGTTGTAAGACCAAGATGAAGAAGAGAAACGTTACATTCTCTTCTACCACACCCGCCC  400
CHIFU-cBrPAP1      TTTGAGTAAGAAACATGAACCAGGCTGTAATACCAAGATGAGAAAGAGAAACATTCCTTGCTCTTCAACCCAACCCGCCC  400
Consensus          t tgag aagaaacatgaacc  g tgtaa acc agatga  aagagaaac tt c t ctctt  acc  acc gccc

CHIFU-cBrMYB2      AAAAAATCGACGTTTTCAAACCTCGACCTCGATCCTTCACCGTTAACAGCGGCTGCAGCCATAATAATGGCATGCCAGAA  480
94S17-cBrmyb2      AAAAAATCGACGTTTTCAAACCTCGACCTCGATCCTTCACCGTTAACAGCGGCTGCAGCCATAATAATGGCATGCCAGAA  480
11S91-cBrMYB2      AAAAAATCGACGTTTTCAAACCTCGACCTCGATCCTTCACCGTTAACAGCGGCTGCAGCCATAATAATGGCATGCCAGAA  480
95T2-5-cBrMYB2     AAAAAATCGACGTTTTCAAACCTCGACCTCGATCCTTCACCGTTAACAGCGGCTGCAGCCATAATAATGGCATGCCAGAA  480
CHIFU-cBrMYB1      AAAAAATCGACGTTTTCAAACCTCGACCTCGACTCTTCACCGTTAACAATGGCTGCAGCCATCTCCATGGCCTGCCAGAA  480
CHIFU-cBrPAP1      AAAAAAATGAAGTTCTCAAACCTCGACCTCGATCCTTCACGGTTAACAACGGCTGCAGCCATTTCAATGGCCAGCCAAAA  480
Consensus          aaaaaa  ga gtt tcaaacctcgacctcga  cttcac gttaaca  ggctgcagccat    atggc  gcca aa

CHIFU-cBrMYB2      GCTGACATTGTTCCTCTATGCCTTGGACACAACGATACTAATAATGTTTCTGAAAATATAATCACATGTAACAAAGATGA  560
94S17-cBrmyb2      GCTGACATTGTTCCTCTATGCCTTGGACACAACGATACTAATAATGTTTCTGAAAATATAATCACATGTAACAAAGATGA  560
11S91-cBrMYB2      GCTGGCATTGTTCCTCTATGCCTTGGACACAACGATACTAATAATGTTTCTGAAAATATAATCACATGTAACAAAGATGA  560
95T2-5-cBrMYB2     GCTGGCATTGTTCCTCTATGCCTTGGACACAACGATACTAATAATGTTTCTGAAAATATAATCACATGTAACAAAGATGA  560
CHIFU-cBrMYB1      GTTGACGTTGTTCCTCCATGCCTTGGACTCAACAACATTAATAATGTCTGTGAAAATAGTATGACATGTAACAAAGCTGG  560
CHIFU-cBrPAP1      GTTGACGTTATTCCTCTATTCCTTGGAGTAAACAACACTAATAATGTTTGTGAAAATAGTATCACATATAAAAAAGATGC  560
Consensus          g tg c tt ttcctc at ccttgga   aac a a taataatgt t tgaaaata  at acat taa aaag tg

CHIFU-cBrMYB2      TGATAAATCTGAGCTTGTTAGTCATTTAATGGATGGTCAGAATAGGTGGTGGAAAGTTTGCTAGATGAGAGCCAAGATC  640
94S17-cBrmyb2      TGATAAATCTGAGCTTGTTAGTCATTTAATGGATGGTCAGAATAGGTGGTGGAAAGTTTGCTAGATGAGAGCCAAGATC  640
11S91-cBrMYB2      TGATAAATCTGAGCTTGTTAGTCATTTAATGGATGGTCAGAATAGGTGGTGGAAAGTTTGCTAGATGAGAGCCAAGATC  640
95T2-5-cBrMYB2     TGATAAATCTGAGCTTGTTAGTCATTTAATGGATGGTCAGAATAGGTGGTGGAAAGTTTGCTAGATGAGAGCCAAGATC  640
CHIFU-cBrMYB1      GGAGAAGTATGAACTTTATAGTAATTTAATGGATGGAGAGAATATGTGGTGGAGAGTTTGCTAGAGGAGAGCAAACAGC  640
CHIFU-cBrPAP1      GGAGAAATATGAGCTCGTTAATAATTTAATGGATGGAGAGAACATGTGGTGGAAGAGTTTGCTAGAGGAGAGCCAAGAAC  640
Consensus           ga aa t tga ct   ta t atttaatggatgg  agaa a gtggtgg a agtttgctaga gagagc aa a c

CHIFU-cBrMYB2      CAGCTGCGCTCTTTCCAGAAACTACAGCAATAAAAAAGGGCGCAACCTCCGCGTTTGACGTTGAGCAACTTTGGAGCCTG  720
94S17-cBrmyb2      CAGCTGCGCTCTTTCCAGAAACTACAGCAATAAAAAAGGGCGCAACCTCCGCGTTTGACGTTGAGCAACTTTGGAGCCTG  720
11S91-cBrMYB2      CAGCTGCGCTCTTTCCAGAAACTACAGCAATAAAAAAGGGCGCAACCTCCGCGTTTGACGTTGAGCAACTTTGGAGCCTG  720
95T2-5-cBrMYB2     CAGCTGCGCTCTTTCCAGAAACTACAGCAATAAAAAAGGGCGCAACCTCCGCGTTTGACGTTGAGCAACTTTGGAGCCTG  720
CHIFU-cBrMYB1      CTGACGGGCTCGTTCCAAAAGGTACGGCAACAAAAAAGGGGGCAACCTTTGCGTTTGACGTTGAGCAACTTTGGAATATG  720
CHIFU-cBrPAP1      CAGTTGCGATTGTTCCAGAATCTACAGAGACAGAAAAGCTAGCAACCTCGGCGTTTGACGTTGAGCAACTTTGGAATCTG  720
Consensus          c g  g g t  ttcca aa  tac g  a a aaaag   gcaacct  gcgtttgacgttgagcaactttgga   tg

CHIFU-cBrMYB2      TTGGATGGAGAAAC..TGGAACTTGA...                                                     744
94S17-cBrmyb2      TTGGATGGAGAAAC..TGGAACTTGA...                                                     744
11S91-cBrMYB2      TTGGATGGAGAAAC..TGGAACTTGA...                                                     744
95T2-5-cBrMYB2     TTGGATGGAGAAAC..TGGAACTTGA...                                                     744
CHIFU-cBrMYB1      TTGGATGGAGAGACTGTAGAACTTGATTA                                                     749
CHIFU-cBrPAP1      TTAGATGGTGAGACCGTGGAACTTGATTA                                                     749
Consensus          tt gatgg ga ac  t gaacttga
```

c Multiple sequence alignment analysis of *cBrMYB2* from different phenotypes of Chinese cabbage. The *cBrMYB2* of ‘Chifu’ was downloaded from BRAD database.

```
11S91-cBrMYB2      ATGGAGGGTTCGTCCCAAGGGTTGAAAAAAGGTGCATGGACTGCTGAAGAAGATAATCTCTTGAGGCAATGCATTGATAA   80
14S838-cBrMYB2     ATGGAGGGTTCGTCCCAAGGGTTGAAAAAAGGTGCATGGACTGCTGAAGAAGATAATCTCTTGAGGCAATGCATTGATAA   80
95T2-5-cBrMYB2     ATGGAGGGTTCGTCCCAAGGGTTGAAAAAAGGTGCATGGACTGCTGAAGAAGATAATCTCTTGAGGCAATGCATTGATAA   80
Z33-cBrMYB2        ATGGAGGGTTCGTCCCAAGGGTTGAAAAAAGGTGCATGGACTGCTGAAGAAGATAATCTCTTGAGGCAATGCATTGATAA   80
Z190-cBrMYB2       ATGGAGGGTTCGTCCCAAGGGTTGAAAAAAGGTGCATGGACTGCTGAAGAAGATAATCTCTTGAGGCAATGCATTGATAA   80
Z240-cBrMYB2       ATGGAGGGTTCGTCCCAAGGGTTGAAAAAAGGTGCATGGACTGCTGAAGAAGATAATCTCTTGAGGCAATGCATTGATAA   80
Chifu-cBrMYB2      ATGGAGGGTTCGTCCCAAGGGTTGAAAAAAGGTGCATGGACTGCTGAAGAAGATAATCTCTTGAGGCAATGCATTGATAA   80
13S93-cBrmyb2      ATGGAGGGTTCGTCCCAAGGGTTGAAAAAAGGTGCATGGACTGCTGAAGAAGATAATCTCTTGAGGCAATGCATTGATAA   80
13S1147-cBrmyb2    ATGGAGGGTTCGTCCCAAGGGTTGAAAAAAGGTGCATGGACTGCTGAAGAAGATAATCTCTTGAGGCAATGCATTGATAA   80
15S1080-cBrmyb2    ATGGAGGGTTCGTCCCAAGGGTTGAAAAAAGGTGCATGGACTGCTGAAGAAGATAATCTCTTGAGGCAATGCATTGATAA   80
15S1084-cBrmyb2    ATGGAGGGTTCGTCCCAAGGGTTGAAAAAAGGTGCATGGACTGCTGAAGAAGATAATCTCTTGAGGCAATGCATTGATAA   80
94S17-cBrmyb2      ATGGAGGGTTCGTCCCAAGGGTTGAAAAAAGGTGCATGGACTGCTGAAGAAGATAATCTCTTGAGGCAATGCATTGATAA   80
Consensus          atggagggttcgtcccaagggttgaaaaaaggtgcatggactgctgaagaagataatctcttgaggcaatgcattgataa

11S91-cBrMYB2      GTATGGAGAAGGGAAATGGCACCAAGTTCCTTTAAGAGCTGGTCTAAATCGGTGCAGGAAGAGTTGTAGACTAAGATGGT   160
14S838-cBrMYB2     GTATGGAGAAGGGAAATGGCACCAAGTTCCTTTAAGAGCTGGTCTAAATCGGTGCAGGAAGAGTTGTAGACTAAGATGGT   160
95T2-5-cBrMYB2     GTATGGAGAAGGGAAATGGCACCAAGTTCCTTTAAGAGCTGGTCTAAATCGGTGCAGGAAGAGTTGTAGACTAAGATGGT   160
Z33-cBrMYB2        GTATGGAGAAGGGAAATGGCACCAAGTTCCTTTAAGAGCTGGTCTAAATCGGTGCAGGAAGAGTTGTAGACTAAGATGGT   160
Z190-cBrMYB2       GTATGGAGAAGGGAAATGGCACCAAGTTCCTTTAAGAGCTGGTCTAAATCGGTGCAGGAAGAGTTGTAGACTAAGATGGT   160
Z240-cBrMYB2       GTATGGAGAAGGGAAATGGCACCAAGTTCCTTTAAGAGCTGGTCTAAATCGGTGCAGGAAGAGTTGTAGACTAAGATGGT   160
Chifu-cBrMYB2      GTATGGAGAAGGGAAATGGCACCAAGTTCCTTTAAGAGCTGGTCTAAATCGGTGCAGGAAGAGTTGTAGACTAAGATGGT   160
13S93-cBrmyb2      GTATGGAGAAGGGAAATGGCACCAAGTTCCTTTAAGAGCTGGTCTAAATCGGTGCAGGAAGAGTTGTAGACTAAGATGGT   160
13S1147-cBrmyb2    GTATGGAGAAGGGAAATGGCACCAAGTTCCTTTAAGAGCTGGTCTAAATCGGTGCAGGAAGAGTTGTAGACTAAGATGGT   160
15S1080-cBrmyb2    GTATGGAGAAGGGAAATGGCACCAAGTTCCTTTAAGAGCTGGTCTAAATCGGTGCAGGAAGAGTTGTAGACTAAGATGGT   160
15S1084-cBrmyb2    GTATGGAGAAGGGAAATGGCACCAAGTTCCTTTAAGAGCTGGTCTAAATCGGTGCAGGAAGAGTTGTAGACTAAGATGGT   160
94S17-cBrmyb2      GTATGGAGAAGGGAAATGGCACCAAGTTCCTTTAAGAGCTGGTCTAAATCGGTGCAGGAAGAGTTGTAGACTAAGATGGT   160
Consensus          gtatggagaagggaaatggcaccaagttcctttaagagctggtctaaatcggtgcaggaagagttgtagactaagatggt

11S91-cBrMYB2      TGAACTATTTGAAGCCAAGTATCAAGAGAGGAAAACTCAACTCCGATGAAGTTGATCTTCTTATTCGCCTTCATAAGCTT   240
14S838-cBrMYB2     TGAACTATTTGAAGCCAAGTATCAAGAGAGGAAAACTCAACTCCGATGAAGTTGATCTTCTTATTCGCCTTCATAAGCTT   240
95T2-5-cBrMYB2     TGAACTATTTGAAGCCAAGTATCAAGAGAGGAAAACTCAACTCCGATGAAGTTGATCTTCTTATTCGCCTTCATAAGCTT   240
Z33-cBrMYB2        TGAACTATTTGAAGCCAAGTATCAAGAGAGGAAAACTCAACTCCGATGAAGTTGATCTTCTTATTCGCCTTCATAAGCTT   240
Z190-cBrMYB2       TGAACTATTTGAAGCCAAGTATCAAGAGAGGAAAACTCAACTCCGATGAAGTTGATCTTCTTATTCGCCTTCATAAGCTT   240
Z240-cBrMYB2       TGAACTATTTGAAGCCAAGTATCAAGAGAGGAAAACTCAACTCCGATGAAGTTGATCTTCTTATTCGCCTTCATAAGCTT   240
Chifu-cBrMYB2      TGAACTATTTGAAGCCAAGTATCAAGAGAGGAAAACTCAACTCCGATGAAGTTGATCTTCTTATGCGCCTTCATAAGCTT   240
13S93-cBrmyb2      TGAACTATTTGAAGCCAAGTATCAAGAGAGGAAAACTCAACTCCGATGAAGTTGATCTTCTTATGCGCCTTCATAAGCTT   240
13S1147-cBrmyb2    TGAACTATTTGAAGCCAAGTATCAAGAGAGGAAAACTCAACTCCGATGAAGTTGATCTTCTTATGCGCCTTCATAAGCTT   240
15S1080-cBrmyb2    TGAACTATTTGAAGCCAAGTATCAAGAGAGGAAAACTCAACTCCGATGAAGTTGATCTTCTTATGCGCCTTCATAAGCTT   240
15S1084-cBrmyb2    TGAACTATTTGAAGCCAAGTATCAAGAGAGGAAAACTCAACTCCGATGAAGTTGATCTTCTTATGCGCCTTCATAAGCTT   240
94S17-cBrmyb2      TGAACTATTTGAAGCCAAGTATCAAGAGAGGAAAACTCAACTCCGATGAAGTTGATCTTCTTATGCGCCTTCATAAGCTT   240
Consensus          tgaactatttgaagccaagtatcaagagaggaaaactcaactccgatgaagttgatcttcttat cgccttcataagctt

11S91-cBrMYB2      TTAGGAAACAGGTGGTCTTTAATTGCTGGTAGATTACCCGGTCGGACCGCCAATGACGTCAAAAATTACTGGAACACCCA   320
14S838-cBrMYB2     TTAGGAAACAGGTGGTCTTTAATTGCTGGTAGATTACCCGGTCGGACCGCCAATGACGTCAAAAATTACTGGAACACCCA   320
95T2-5-cBrMYB2     TTAGGAAACAGGTGGTCTTTAATTGCTGGTAGATTACCCGGTCGGACCGCCAATGACGTCAAAAATTACTGGAACACCCA   320
Z33-cBrMYB2        TTAGGAAACAGGTGGTCTTTAATTGCTGGTAGATTACCCGGTCGGACCGCCAATGACGTCAAAAATTACTGGAACACCCA   320
Z190-cBrMYB2       TTAGGAAACAGGTGGTCTTTAATTGCTGGTAGATTACCCGGTCGGACCGCCAATGACGTCAAAAATTACTGGAACACCCA   320
Z240-cBrMYB2       TTAGGAAACAGGTGGTCTTTAATTGCTGGTAGATTACCCGGTCGGACCGCCAATGACGTCAAAAATTACTGGAACACCCA   320
Chifu-cBrMYB2      TTAGGAAACAGGTGGTCTTTAATTGCTGGTAGATTACCCGGTCGGACCGCCAATGACGTCAAAAATTACTGGAACACCCA   320
13S93-cBrmyb2      TTAGGAAACAGGTGGTCTTTAATTGCTGGTAGATTACCCGGTCGGACCGCCAATGACGTCAAAAATTACTGGAACACCCA   320
13S1147-cBrmyb2    TTAGGAAACAGGTGGTCTTTAATTGCTGGTAGATTACCCGGTCGGACCGCCAATGACGTCAAAAATTACTGGAACACCCA   320
15S1080-cBrmyb2    TTAGGAAACAGGTGGTCTTTAATTGCTGGTAGATTACCCGGTCGGACCGCCAATGACGTCAAAAATTACTGGAACACCCA   320
15S1084-cBrmyb2    TTAGGAAACAGGTGGTCTTTAATTGCTGGTAGATTACCCGGTCGGACCGCCAATGACGTCAAAAATTACTGGAACACCCA   320
94S17-cBrmyb2      TTAGGAAACAGGTGGTCTTTAATTGCTGGTAGATTACCCGGTCGGACCGCCAATGACGTCAAAAATTACTGGAACACCCA   320
Consensus          ttaggaaacaggtggtctttaattgctggtagattacccggtcggaccgccaatgacgtcaaaaattactggaacaccca

11S91-cBrMYB2      TTTGAGTAAGAAACATGAACCGGGTTGTAAGACCCAGATGAAAAAGAGAAACATTCCTTGCTCTTATACCACACCAGCCC   400
14S838-cBrMYB2     TTTGAGTAAGAAACATGAACCGGGTTGTAAGACCCAGATGAAAAAGAGAAACATTCCTTGCTCTTATACCACACCAGCCC   400
95T2-5-cBrMYB2     TTTGAGTAAGAAACATGAACCGGGTTGTAAGACCCAGATGAAAAAGAGAAACATTCCTTGCTCTTATACCACACCAGCCC   400
Z33-cBrMYB2        TTTGAGTAAGAAACATGAACCGGGTTGTAAGACCCAGATGAAAAAGAGAAACATTCCTTGCTCTTATACCACACCAGCCC   400
Z190-cBrMYB2       TTTGAGTAAGAAACATGAACCGGGTTGTAAGACCCAGATGAAAAAGAGAAACATTCCTTGCTCTTATACCACACCAGCCC   400
Z240-cBrMYB2       TTTGAGTAAGAAACATGAACCGGGTTGTAAGACCCAGATGAAAAAGAGAAACATTCCTTGCTCTTATACCACACCAGCCC   400
Chifu-cBrMYB2      TTTGAGTAAGAAACATGAACCGGGTTGTAAGACCCAGATGAAAAAGAGAAACATTCCTTGCTCTTATACCACACCAGCCC   400
13S93-cBrmyb2      TTTGAGTAAGAAACATGAACCGGGTTGTAAGACCCAGATGAAAAAGAGAAACATTCCTTGCTCTTATACCACACCAGCCC   400
13S1147-cBrmyb2    TTTGAGTAAGAAACATGAACCGGGTTGTAAGACCCAGATGAAAAAGAGAAACATTCCTTGCTCTTATACCACACCAGCCC   400
15S1080-cBrmyb2    TTTGAGTAAGAAACATGAACCGGGTTGTAAGACCCAGATGAAAAAGAGAAACATTCCTTGCTCTTATACCACACCAGCCC   400
15S1084-cBrmyb2    TTTGAGTAAGAAACATGAACCGGGTTGTAAGACCCAGATGAAAAAGAGAAACATTCCTTGCTCTTATACCACACCAGCCC   400
94S17-cBrmyb2      TTTGAGTAAGAAACATGAACCGGGTTGTAAGACCCAGATGAAAAAGAGAAACATTCCTTGCTCTTATACCACACCAGCCC   400
Consensus          tttgagtaagaaacatgaaccgggttgtaagacccagatgaaaaagagaaacattccttgctcttataccacaccagccc
```

```
11S91-cBrMYB2       AAAAAATCGACGTTTTCAAACCTCGACCTCGATCCTTCACCGTTAACAGCGGCTGCAGCCATAATAATGGCATGCCAGAA   480
14S838-cBrMYB2      AAAAAATCGACGTTTTCAAACCTCGACCTCGATCCTTCACCGTTAACAGCGGCTGCAGCCATAATAATGGCATGCCAGAA   480
95T2-5-cBrMYB2      AAAAAATCGACGTTTTCAAACCTCGACCTCGATCCTTCACCGTTAACAGCGGCTGCAGCCATAATAATGGCATGCCAGAA   480
Z33-cBrMYB2         AAAAAATCGACGTTTTCAAACCTCGACCTCGATCCTTCACCGTTAACAGCGGCTGCAGCCATAATAATGGCATGCCAGAA   480
Z190-cBrMYB2        AAAAAATCGACGTTTTCAAACCTCGACCTCGATCCTTCACCGTTAACAGCGGCTGCAGCCATAATAATGGCATGCCAGAA   480
Z240-cBrMYB2        AAAAAATCGACGTTTTCAAACCTCGACCTCGATCCTTCACCGTTAACAGCGGCTGCAGCCATAATAATGGCATGCCAGAA   480
Chifu-cBrMYB2       AAAAAATCGACGTTTTCAAACCTCGACCTCGATCCTTCACCGTTAACAGCGGCTGCAGCCATAATAATGGCATGCCAGAA   480
13S93-cBrmyb2       AAAAAATCGACGTTTTCAAACCTCGACCTCGATCCTTCACCGTTAACAGCGGCTGCAGCCATAATAATGGCATGCCAGAA   480
13S1147-cBrmyb2     AAAAAATCGACGTTTTCAAACCTCGACCTCGATCCTTCACCGTTAACAGCGGCTGCAGCCATAATAATGGCATGCCAGAA   480
15S1080-cBrmyb2     AAAAAATCGACGTTTTCAAACCTCGACCTCGATCCTTCACCGTTAACAGCGGCTGCAGCCATAATAATGGCATGCCAGAA   480
15S1084-cBrmyb2     AAAAAATCGACGTTTTCAAACCTCGACCTCGATCCTTCACCGTTAACAGCGGCTGCAGCCATAATAATGGCATGCCAGAA   480
94S17-cBrmyb2       AAAAAATCGACGTTTTCAAACCTCGACCTCGATCCTTCACCGTTAACAGCGGCTGCAGCCATAATAATGGCATGCCAGAA   480
Consensus           aaaaaatcgacgttttcaaacctcgacctcgatccttcaccgttaacagcggctgcagccataataatggcatgccagaa

11S91-cBrMYB2       GCTGGCATTGTTCCTCTATGCCTTGGACACAACGATACTAATAATGTTTCTGAAAATATAATCACATGTAACAAAGATGA   560
14S838-cBrMYB2      GCTGGCATTGTTCCTCTATGCCTTGGACACAACGATACTAATAATGTTTCTGAAAATATAATCACATGTAACAAAGATGA   560
95T2-5-cBrMYB2      GCTGGCATTGTTCCTCTATGCCTTGGACACAACGATACTAATAATGTTTCTGAAAATATAATCACATGTAACAAAGATGA   560
Z33-cBrMYB2         GCTGGCATTGTTCCTCTATGCCTTGGACACAACGATACTAATAATGTTTCTGAAAATATAATCACATGTAACAAAGATGA   560
Z190-cBrMYB2        GCTGGCATTGTTCCTCTATGCCTTGGACACAACGATACTAATAATGTTTCTGAAAATATAATCACATGTAACAAAGATGA   560
Z240-cBrMYB2        GCTGGCATTGTTCCTCTATGCCTTGGACACAACGATACTAATAATGTTTCTGAAAATATAATCACATGTAACAAAGATGA   560
Chifu-cBrMYB2       GCTGACATTGTTCCTCTATGCCTTGGACACAACGATACTAATAATGTTTCTGAAAATATAATCACATGTAACAAAGATGA   560
13S93-cBrmyb2       GCTGACATTGTTCCTCTATGCCTTGGACACAACGATACTAATAATGTTTCTGAAAATATAATCACATGTAACAAAGATGA   560
13S1147-cBrmyb2     GCTGACATTGTTCCTCTATGCCTTGGACACAACGATACTAATAATGTTTCTGAAAATATAATCACATGTAACAAAGATGA   560
15S1080-cBrmyb2     GCTGACATTGTTCCTCTATGCCTTGGACACAACGATACTAATAATGTTTCTGAAAATATAATCACATGTAACAAAGATGA   560
15S1084-cBrmyb2     GCTGACATTGTTCCTCTATGCCTTGGACACAACGATACTAATAATGTTTCTGAAAATATAATCACATGTAACAAAGATGA   560
94S17-cBrmyb2       GCTGACATTGTTCCTCTATGCCTTGGACACAACGATACTAATAATGTTTCTGAAAATATAATCACATGTAACAAAGATGA   560
Consensus           gctg cattgttcctctatgccttggacacaacgatactaataatgtttctgaaaatataatcacatgtaacaaagatga

11S91-cBrMYB2       TGATAAATCTGAGCTTGTTAGTCATTTAATGGATGGTCAGAATAGGTGGTGGGAAAGTTTGCTAGATGAGAGCCAAGATC   640
14S838-cBrMYB2      TGATAAATCTGAGCTTGTTAGTCATTTAATGGATGGTCAGAATAGGTGGTGGGAAAGTTTGCTAGATGAGAGCCAAGATC   640
95T2-5-cBrMYB2      TGATAAATCTGAGCTTGTTAGTCATTTAATGGATGGTCAGAATAGGTGGTGGGAAAGTTTGCTAGATGAGAGCCAAGATC   640
Z33-cBrMYB2         TGATAAATCTGAGCTTGTTAGTCATTTAATGGATGGTCAGAATAGGTGGTGGGAAAGTTTGCTAGATGAGAGCCAAGATC   640
Z190-cBrMYB2        TGATAAATCTGAGCTTGTTAGTCATTTAATGGATGGTCAGAATAGGTGGTGGGAAAGTTTGCTAGATGAGAGCCAAGATC   640
Z240-cBrMYB2        TGATAAATCTGAGCTTGTTAGTCATTTAATGGATGGTCAGAATAGGTGGTGGGAAAGTTTGCTAGATGAGAGCCAAGATC   640
Chifu-cBrMYB2       TGATAAATCTGAGCTTGTTAGTCATTTAATGGATGGTCAGAATAGGTGGTGGGAAAGTTTGCTAGATGAGAGCCAAGATC   640
13S93-cBrmyb2       TGATAAATCTGAGCTTGTTAGTCATTTAATGGATGGTCAGAATAGGTGGTGGGAAAGTTTGCTAGATGAGAGCCAAGATC   640
13S1147-cBrmyb2     TGATAAATCTGAGCTTGTTAGTCATTTAATGGATGGTCAGAATAGGTGGTGGGAAAGTTTGCTAGATGAGAGCCAAGATC   640
15S1080-cBrmyb2     TGATAAATCTGAGCTTGTTAGTCATTTAATGGATGGTCAGAATAGGTGGTGGGAAAGTTTGCTAGATGAGAGCCAAGATC   640
15S1084-cBrmyb2     TGATAAATCTGAGCTTGTTAGTCATTTAATGGATGGTCAGAATAGGTGGTGGGAAAGTTTGCTAGATGAGAGCCAAGATC   640
94S17-cBrmyb2       TGATAAATCTGAGCTTGTTAGTCATTTAATGGATGGTCAGAATAGGTGGTGGGAAAGTTTGCTAGATGAGAGCCAAGATC   640
Consensus           tgataaatctgagcttgttagtcatttaatggatggtcagaataggtggtgggaaagtttgctagatgagagccaagatc

11S91-cBrMYB2       CAGCTGCGCTCTTTCCAGAAACTACAGCAATAAAAAAGGGCGCAACCTCCGCGTTTGACGTTGAGCAACTTTGGAGCCTG   720
14S838-cBrMYB2      CAGCTGCGCTCTTTCCAGAAACTACAGCAATAAAAAAGGGCGCAACCTCCGCGTTTGACGTTGAGCAACTTTGGAGCCTG   720
95T2-5-cBrMYB2      CAGCTGCGCTCTTTCCAGAAACTACAGCAATAAAAAAGGGCGCAACCTCCGCGTTTGACGTTGAGCAACTTTGGAGCCTG   720
Z33-cBrMYB2         CAGCTGCGCTCTTTCCAGAAACTACAGCAATAAAAAAGGGCGCAACCTCCGCGTTTGACGTTGAGCAACTTTGGAGCCTG   720
Z190-cBrMYB2        CAGCTGCGCTCTTTCCAGAAACTACAGCAATAAAAAAGGGCGCAACCTCCGCGTTTGACGTTGAGCAACTTTGGAGCCTG   720
Z240-cBrMYB2        CAGCTGCGCTCTTTCCAGAAACTACAGCAATAAAAAAGGGCGCAACCTCCGCGTTTGACGTTGAGCAACTTTGGAGCCTG   720
Chifu-cBrMYB2       CAGCTGCGCTCTTTCCAGAAACTACAGCAATAAAAAAGGGCGCAACCTCCGCGTTTGACGTTGAGCAACTTTGGAGCCTG   720
13S93-cBrmyb2       CAGCTGCGCTCTTTCCAGAAACTACAGCAATAAAAAAGGGCGCAACCTCCGCGTTTGACGTTGAGCAACTTTGGAGCCTG   720
13S1147-cBrmyb2     CAGCTGCGCTCTTTCCAGAAACTACAGCAATAAAAAAGGGCGCAACCTCCGCGTTTGACGTTGAGCAACTTTGGAGCCTG   720
15S1080-cBrmyb2     CAGCTGCGCTCTTTCCAGAAACTACAGCAATAAAAAAGGGCGCAACCTCCGCGTTTGACGTTGAGCAACTTTGGAGCCTG   720
15S1084-cBrmyb2     CAGCTGCGCTCTTTCCAGAAACTACAGCAATAAAAAAGGGCGCAACCTCCGCGTTTGACGTTGAGCAACTTTGGAGCCTG   720
94S17-cBrmyb2       CAGCTGCGCTCTTTCCAGAAACTACAGCAATAAAAAAGGGCGCAACCTCCGCGTTTGACGTTGAGCAACTTTGGAGCCTG   720
Consensus           cagctgcgctctttccagaaactacagcaataaaaaagggcgcaacctccgcgtttgacgttgagcaactttggagcctg

11S91-cBrMYB2       TTGGATGGAGAAACTGGAACTTG   743
14S838-cBrMYB2      TTGGATGGAGAAACTGGAACTTG   743
95T2-5-cBrMYB2      TTGGATGGAGAAACTGGAACTTG   743
Z33-cBrMYB2         TTGGATGGAGAAACTGGAACTTG   743
Z190-cBrMYB2        TTGGATGGAGAAACTGGAACTTG   743
Z240-cBrMYB2        TTGGATGGAGAAACTGGAACTTG   743
Chifu-cBrMYB2       TTGGATGGAGAAACTGGAACTTG   743
13S93-cBrmyb2       TTGGATGGAGAAACTGGAACTTG   743
13S1147-cBrmyb2     TTGGATGGAGAAACTGGAACTTG   743
15S1080-cBrmyb2     TTGGATGGAGAAACTGGAACTTG   743
15S1084-cBrmyb2     TTGGATGGAGAAACTGGAACTTG   743
94S17-cBrmyb2       TTGGATGGAGAAACTGGAACTTG   743
Consensus           ttggatggagaaactggaacttg
```

d Multiple sequence alignment analysis of g*BrMYB2* between purple head Chinese cabbage '11S91' and its female parent '94S17'. '95T2-5' and '11S91' had consensus genomic DNA sequence (gDNA).

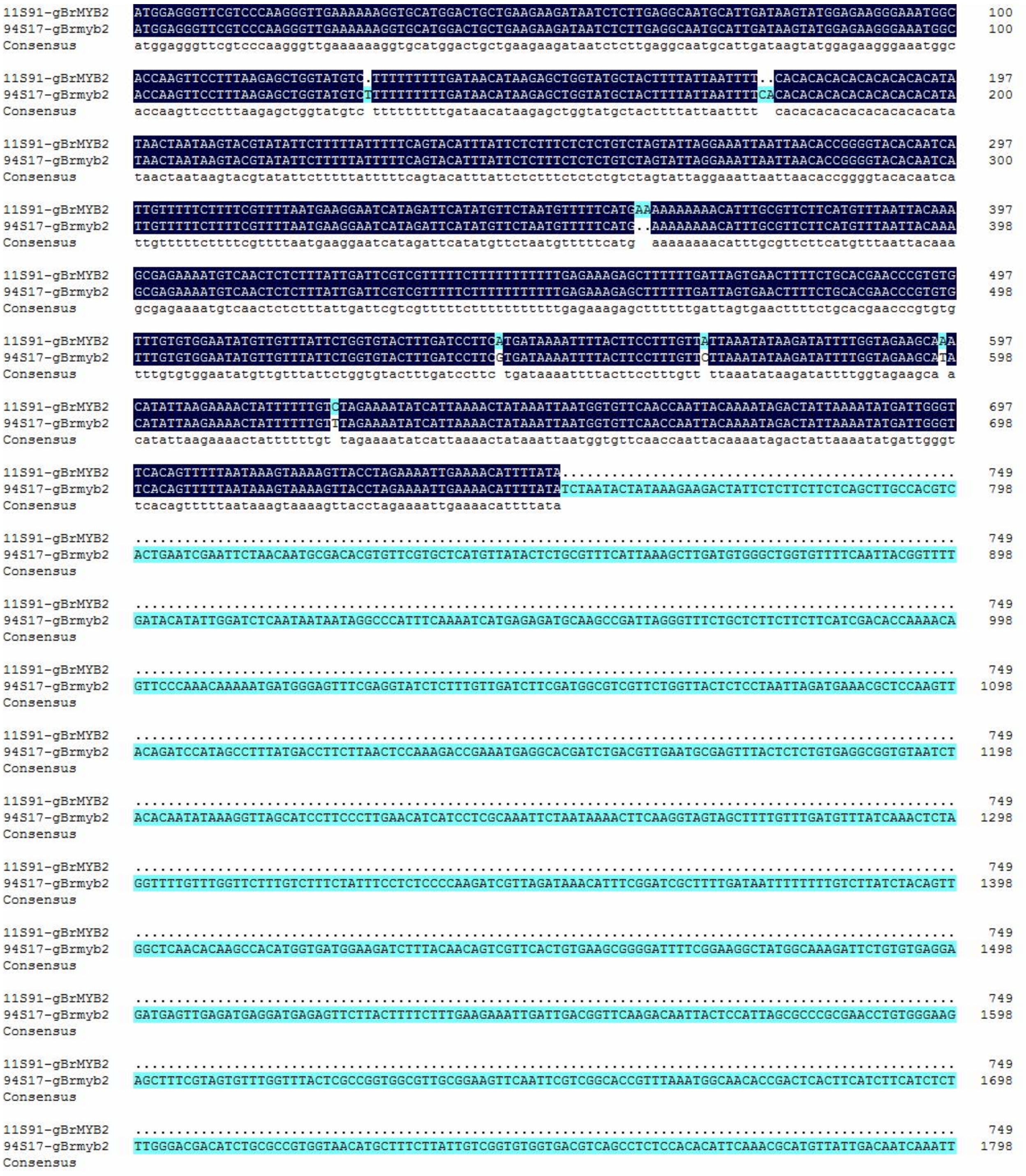

```
11S91-gBrMYB2   ....................................................................................................   749
94S17-gBrmyb2   ATTATGCAGGCTTTCTCTCAACATACAGAGCTTCCATAGTTATTGTTAAATCTTAATCATGTCTGAGGATACCATCACCGTCCCTCTCAGCTGCTGGAAT   1898
Consensus

11S91-gBrMYB2   ....................................................................................................   749
94S17-gBrmyb2   GTACTCAGCTTGGTTGGATGGAGCGTCTGCTTGGCTGGAATTCAGAAAGTTCTGCTCCAAAGGAACCTGTCTGAACGTCAATGATGCCTTTGGCCAAGAA   1998
Consensus

11S91-gBrMYB2   ....................................................................................................   749
94S17-gBrmyb2   TTATAACATGGTGGCTGATCCAAGAAATACCTGAACATTGCAATCTTCTTAAGAGCACCGAATGTAACATGTGACGAGGTGTCAAAAGCTCTTCTAGATT   2098
Consensus

11S91-gBrMYB2   ....................................................................................................   749
94S17-gBrmyb2   GTAAGATTGGTCTCCCATTTGGTTTGTTACTTAGGGGTAAAGTTCTATTTGCTTTGATCACTTCAACTTGCAAAATAGTGTTAACGGAACACATTCTATG   2198
Consensus

11S91-gBrMYB2   ....................................................................................................   749
94S17-gBrmyb2   CACTAATCTTATTTCCATCATTTTCTATTCACTCCACTAATCAAGTTCAGTCCAACGACTATTTATCATCCAGGAATCAAGTTCTATTTGTTAAATATAT   2298
Consensus

11S91-gBrMYB2   ....................................................................................................   749
94S17-gBrmyb2   TTGTGAATCACACACAGCCAAGCTATCCACAAATTTATTGAACAATCTCTTCAAAGACAAACTTATAATACACTGCCACTTTCAAAAAATGTTTTGTGTA   2398
Consensus

11S91-gBrMYB2   ....................................................................................................   749
94S17-gBrmyb2   CACTTCTTTGGACAGACGAAAACTATGACCGTCTTATTAATGGTAAGTCTGGCTCAAACCAAGCATCAATGCCATCACGTTCCTGCTCTTATTATTCTGA   2498
Consensus

11S91-gBrMYB2   ....................................................................................................   749
94S17-gBrmyb2   AAGGAGCAAGAGGAAAGCAGGTTGTAGCACAGTCATGTTCCAGCTCCATATTTCACAGAAGCCATCATTGCCGAGTTGAGTTGCAGTATTGTGTAAAAGT   2598
Consensus

11S91-gBrMYB2   ....................................................................................................   749
94S17-gBrmyb2   TTCTAACCACGAGGAGTTTAGTGTTGGAAAATATATTGGAGGTGCAGTGTTTATACCGAAAAGTTCTCAGGCAGAGATATTGTGTAGGTCTAAATGTCAT   2698
Consensus

11S91-gBrMYB2   ....................................................................................................   749
94S17-gBrmyb2   AAAAGGACAGAAAATATAGTAGATGGAATTCTGTTTTTGTGTCCAGTTATATTCTCCGACTGTAGCTTGAAGAGCAAACTATCTAAGAGAGGTGAGATTG   2798
Consensus

11S91-gBrMYB2   ....................................................................................................   749
94S17-gBrmyb2   GTTTTAATCTGCCATTTCTGGAGAGTTGTGAAGATGTTGAAGGAGAGCAAGGTGAAGAATGTGATTGCTCACGCTGGGTTGTAAGAGACAGTCTTAGCTA   2898
Consensus

11S91-gBrMYB2   ....................................................................................................   749
94S17-gBrmyb2   TTGGTTTCTGATTCTCAGATTTTTCAGCTGCAAATCTCAGACCGGTTTCTAAACACCAAAACAGCCTTTGGAAGACTTTCTTTTGTTGTGTGTTCTCCTG   2998
Consensus

11S91-gBrMYB2   ....................................................................................................   749
94S17-gBrmyb2   GCGGTGATGGGCCTACGGATCGCTGTGGTGACAGTGCAAAGGGGAGTGTTGTCGGGTGGTTATCAATTCAATACTATACAATTCTTTGCTTACAAAGCTG   3098
Consensus

11S91-gBrMYB2   ....................................................................................................   749
94S17-gBrmyb2   TGTAAGAGTATATTGTTATTATATAATGGTGAAAGAGTCGAAGTTGATGCTCACTGGAAGGAAATGAAGTAATGGAAAAAAAACTCCACTAGATGCAAGA   3198
Consensus

11S91-gBrMYB2   ....................................................................................................   749
94S17-gBrmyb2   AACAATAGTTTTCTAAGTAGCGTTGAAATATTATCAATAAAAATAAAAGTATATAACAAACAATGTTTATTTGATAAGATTTCTAAACAATAAAGAGTGT   3298
Consensus

11S91-gBrMYB2   ....................................................................................................   749
94S17-gBrmyb2   TAAAGATTCGGCAAATAAATTATTTAGAATGTACATGATACAAAATATTGCAAAGTTTTTTTTTTTTTTTTGCTAAAAGTAAATGTGTTGTGTTGATTTA   3398
Consensus

11S91-gBrMYB2   ....................................................................................................   749
94S17-gBrmyb2   AAATATTGCAAAGTTGTTATGAAAAATGTAATATCTGAAGATACCATCGAAAACATTAAAACATTAAATTTTAACACATTTCCAACAGTTAAAAATATCA   3498
Consensus

11S91-gBrMYB2   ....................................................................................................   749
94S17-gBrmyb2   ATTATCTTATATTATAAATGAATATGCTACGCAAAAAAATGTACAATAATTCTTTTTATCAGCTACAGAAAACAGTTATGAAATTTTGTAACAAACATCAA   3598
Consensus

11S91-gBrMYB2   ....................................................................................................   749
94S17-gBrmyb2   ATGTATTATTAACCGAAAGTCAACATTTTATCTATTAAAAAGTATTATACATGCTTATCAACGTCCCTACCACAAATTATAACTAATTAAATCAAGTTGA   3698
Consensus
```

```
11S91-gBrMYB2   ....................................................................................................   749
94S17-gBrmyb2   AGCCAATATCTAATACTCAATATTCTTTGTTCCAAAATCTTAATAATAAAAATGAAAAAATATTAGCAAAACATTGTTTACTTTTAATTTATTTTTGAAA   3798
Consensus

11S91-gBrMYB2   ....................................................................................................   749
94S17-gBrmyb2   ATTATTTAGTATTAGTTAAGTTTATTAAATATTTAATTTGATATTGTAAATTTGAATATATACAATCAAATACACATTTTTAAAAAAATAGATGAAAGTA   3898
Consensus

11S91-gBrMYB2   ....................................................................................................   749
94S17-gBrmyb2   TTTTTAAAACACGTATGTGTACCTAATAAATATGCAAAAAATCATACATGATAACTTTTTAAAAATAAAAAGCAATTAAATGTTATATGTAGATTTATTA   3998
Consensus

11S91-gBrMYB2   ....................................................................................................   749
94S17-gBrmyb2   TTTATTATTTATGTCATTCAGATAAAAATATAGCAAAGGTGTAATAATATTCATAATATATACTAGAAAATTATATGCATCATTAAATTATATATTACTA   4098
Consensus

11S91-gBrMYB2   ....................................................................................................   749
94S17-gBrmyb2   AAAACTAATTTAAATTTTATAAACAATAAAATATAATTATGAAAGTAGTTTATATAATGAATAAAATATACAAAATTATAGTTAATTTAAATTAATAATG   4198
Consensus

11S91-gBrMYB2   ....................................................................................................   749
94S17-gBrmyb2   ATATGAAAATAAAAATATGTCTTTACATAATCGAACAAAGAAAAAAATATAAAATAGTCTGACAACAAACAAAGATAGAAGAAATGAAATTAGTTGATTT   4298
Consensus

11S91-gBrMYB2   ....................................................................................................   749
94S17-gBrmyb2   ATACGAAACAAATAAGTTAAAATATATATACTAACATAATAAAAATATAATACAATATTATTAAAAATCACAATAATTGTATTAGAAACCTACTAAATAG   4398
Consensus

11S91-gBrMYB2   ....................................................................................................   749
94S17-gBrmyb2   TAACAAAACTGACTTGAAAGAAAACAATTTTAAAAATAATGACCTGGATAAAATAATCATTTTAAAACAAAAATTTTAAAAATTATTTAATTTTCCGCCC   4498
Consensus

11S91-gBrMYB2   ........................TATTGGATAATTAAAACATCAAAA..TTCAATAAAACATCCTATTTTTAGGAACATATGGAGTAGGAGACGCGAAT   823
94S17-gBrmyb2   GCCCGTAGGGCGGGTTTACTCTAGTATTGGATAATTGAAACATCAAAATTTTCAGTAAAACATCCTATTTTTAGGAACATATGGAGTAGGAGACGCGAAT   4598
Consensus                               tattggataatt aaacatcaaaa  ttca taaaacatcctatttttaggaacatatggagtaggagacgcgaat

11S91-gBrMYB2   GCAGTTTTTGCTCGTTCTTTTAATAATATTAAATGTCAATTATTGGTTTTGTAGGTCTAAATCGGTGCAGGAAGAGTTGTAGACTAAGATGGTTGAACTA   923
94S17-gBrmyb2   GCAGTTTTTGCTCGTTCTTTTAATAATATTAAATGTCAATTATTGGTTTTGTAGGTCTAAATCGGTGCAGGAAGAGTTGTAGACTAAGATGGTTGAACTA   4698
Consensus       gcagtttttgctcgttcttttaataatattaaatgtcaattattggttttgtaggtctaaatcggtgcaggaagagttgtagactaagatggttgaacta

11S91-gBrMYB2   TTTGAAGCCAAGTATCAAGAGAGGAAAACTCAACTCCGATGAAGTTGATCTTCTTATTCGCCTTCATAAGCTTTTAGGAAACAGGTTTACATTCCAGACA   1023
94S17-gBrmyb2   TTTGAAGCCAAGTATCAAGAGAGGAAAACTCAACTCCGATGAAGTTGATCTTCTTATGCGCCTTCATAAGCTTTTAGGAAACAGGTTTACATTCAAGACA   4798
Consensus       tttgaagccaagtatcaagagaggaaaactcaactccgatgaagttgatcttcttat cgccttcataagcttttaggaaacaggtttacattc agaca

11S91-gBrMYB2   CAAATTCAACTGTATTTCGTATCCTCATTCGGTCTAATCTAATCATTTGATTTGTTTTTTTTTTTGATAAAAA.TACTTAAATTTATTTCATATGTAAAT   1122
94S17-gBrmyb2   CAAATTCAACTGTATTTCGTATCCTCATTCGGTCTAATCTAATCATTTGATTTGTTTTTTTTTTTGATAAAAAGTACTTAAATTTATTTCATATGTAAAT   4898
Consensus       caaattcaactgtatttcgtatcctcattcggtctaatctaatcatttgatttgtttttttttttgataaaaa tacttaaatttatttcatatgtaaat

11S91-gBrMYB2   GATCCATTACTAAGTCAAATATATCCCTAATTTTTCAAATGCATGCTTAGGTGGTCTTTAATTGCTGGTAGATTACCCGGTCGGACCGCCAATGACGTCA   1222
94S17-gBrmyb2   GATCCATTACTAAGTCAAATATATCCCTAATTTTTCAAATGCATGCTTAGGTGGTCTTTAATTGCTGGTAGATTACCCGGTCGGACCGCCAATGACGTCA   4998
Consensus       gatccattactaagtcaaatatatccctaatttttcaaatgcatgcttaggtggtctttaattgctggtagattacccggtcggaccgccaatgacgtca

11S91-gBrMYB2   AAAATTACTGGAACACCCATTTGAGTAAGAAACATGAACCGGGTTGTAAGACCCAGATGAAAAAGAGAAACATTCCTTGCTCTTATACCACACCAGCCCA   1322
94S17-gBrmyb2   AAAATTACTGGAACACCCATTTGAGTAAGAAACATGAACCGGGTTGTAAGACCCAGATGAAAAAGAGAAACATTCCTTGCTCTTATACCACACCAGCCCA   5098
Consensus       aaaattactggaacacccatttgagtaagaaacatgaaccgggttgtaagacccagatgaaaaagagaaacattccttgctcttataccacaccagccca

11S91-gBrMYB2   AAAAATCGACGTTTTCAAACCTCGACCTCGATCCTTCACCGTTAACAGCGGCTGCAGCCATAATAATGGCATGCCAGAAGCTGGCATTGTTCCTCTATGC   1422
94S17-gBrmyb2   AAAAATCGACGTTTTCAAACCTCGACCTCGATCCTTCACCGTTAACAGCGGCTGCAGCCATAATAATGGCATGCCAGAAGCTGACATTGTTCCTCTATGC   5198
Consensus       aaaaatcgacgttttcaaacctcgacctcgatccttcaccgttaacagcggctgcagccataataatggcatgccagaagctg cattgttcctctatgc

11S91-gBrMYB2   CTTGGACACAACGATACTAATAATGTTTCTGAAAATATAATCACATGTAACAAAGATGATGATAAATCTGAGCTTGTTAGTCATTTAATGGATGGTCAGA   1522
94S17-gBrmyb2   CTTGGACACAACGATACTAATAATGTTTCTGAAAATATAATCACATGTAACAAAGATGATGATAAATCTGAGCTTGTTAGTCATTTAATGGATGGTCAGA   5298
Consensus       cttggacacaacgatactaataatgtttctgaaaatataatcacatgtaacaaagatgatgataaatctgagcttgttagtcatttaatggatggtcaga

11S91-gBrMYB2   ATAGGTGGTGGGAAAGTTTGCTAGATGAGAGCCAAGATCCAGCTGCGCTCTTTCCAGAAACTACAGCAATAAAAAAGGGCGCAACCTCCGCGTTTGACGT   1622
94S17-gBrmyb2   ATAGGTGGTGGGAAAGTTTGCTAGATGAGAGCCAAGATCCAGCTGCGCTCTTTCCAGAAACTACAGCAATAAAAAAGGGCGCAACCTCCGCGTTTGACGT   5398
Consensus       ataggtggtgggaaagtttgctagatgagagccaagatccagctgcgctctttccagaaactacagcaataaaaaagggcgcaacctccgcgtttgacgt

11S91-gBrMYB2   TGAGCAACTTTGGAGCCTGTTGGATGGAGAAACTGGAACTTG                                                             1664
94S17-gBrmyb2   TGAGCAACTTTGGAGCCTGTTGGATGGAGAAACTGGAACTTG                                                             5440
Consensus       tgagcaactttggagcctgttggatggagaaactggaacttg
```

e Multiple sequence alignment analysis of *gBrMYB2* from different phenotypes of Chinese cabbage. The *gBrMYB2* of 'Chifu' was downloaded from BRAD database.

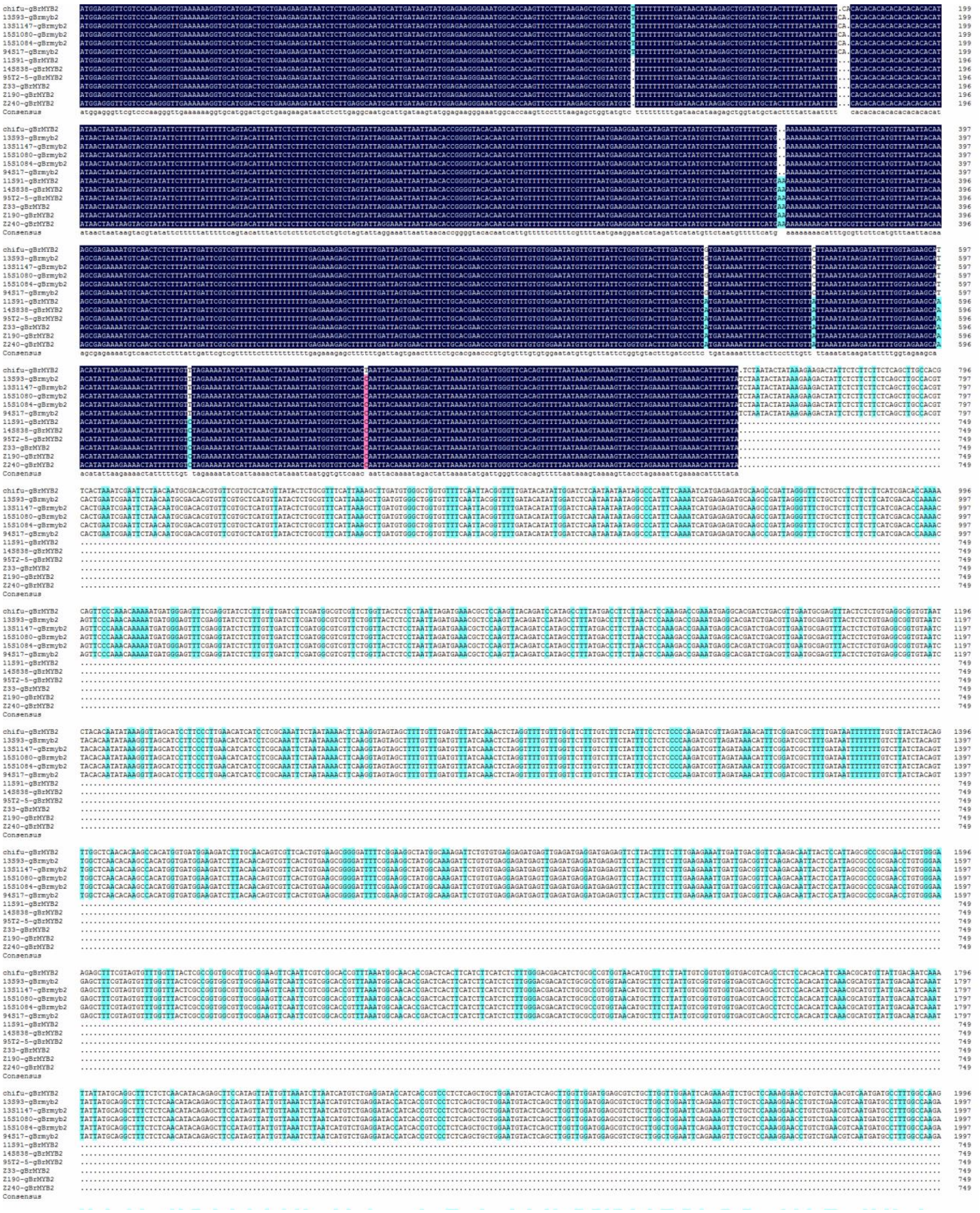

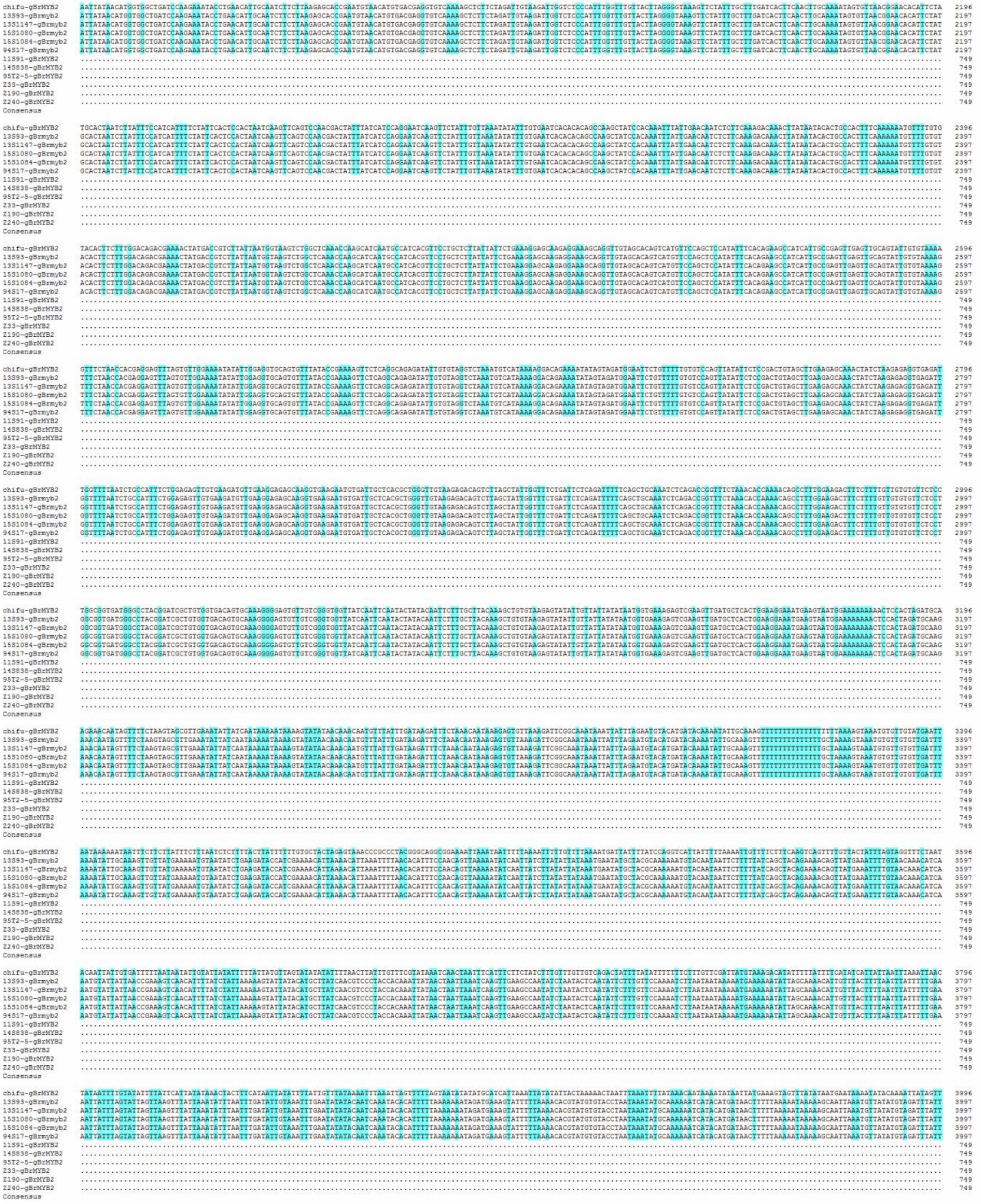

chifu-gBrMYB2
13S93-gBrmyb2
13S1147-gBrmyb2
15S1080-gBrmyb2
15S1084-gBrmyb2
94S17-gBrmyb2
11S91-gBrMYB2
14S838-gBrMYB2
95T2-5-gBrMYB2
Z33-gBrMYB2
Z190-gBrMYB2
Z240-gBrMYB2
Consensus

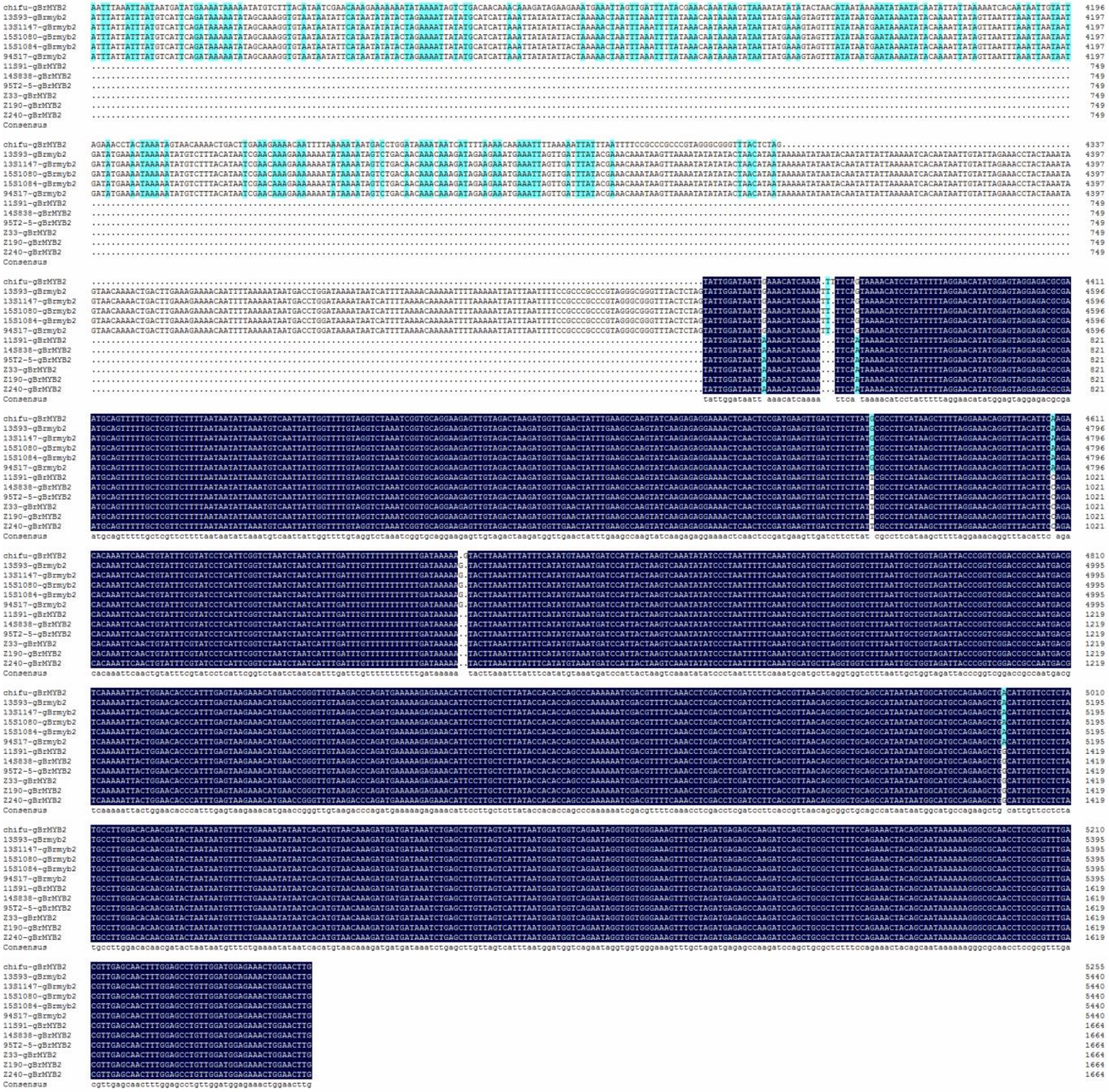

Figure S3 Multiple sequence alignment analysis of BrMYB2 in different Chinese cabbages. ‘11S91’ and ‘95T2-5’ had consensus BrMYB2 sequences, whereas ‘Chifu’ and ‘94S17’ had consensus BrMYB2 sequences.

```
BrMYB2-94S17.txt   MEGSSQGLKKGAWTAEEDNLLRQCIDKYGEGKWHQVPLRA    40
BrMYB2-11S91.txt   MEGSSQGLKKGAWTAEEDNLLRQCIDKYGEGKWHQVPLRA    40
Consensus          megssqglkkgawtaeednllrqcidkygegkwhqvplra

BrMYB2-94S17.txt   GLNRCRKSCRLRWLNYIKPSIKRGKLNSDEVDLLMRLHKL    80
BrMYB2-11S91.txt   GLNRCRKSCRLRWLNYIKPSIKRGKLNSDEVDLLIRLHKL    80
Consensus          glnrcrkscrlrwlnyikpsikrgklnsdevdll rlhkl

BrMYB2-94S17.txt   LGNRWSLIAGRLPGRTANDVKNYWNTHLSKKHEPGCKTQM   120
BrMYB2-11S91.txt   LGNRWSLIAGRLPGRTANDVKNYWNTHLSKKHEPGCKTQM   120
Consensus          lgnrwsliagrlpgrtandvknywnthlskkhepgcktqm

BrMYB2-94S17.txt   KKRNIPCSYTTPAQKIIVFKPRPRSFTVNSGCSHNNGMPE   160
BrMYB2-11S91.txt   KKRNIPCSYTTPAQKIIVFKPRPRSFTVNSGCSHNNGMPE   160
Consensus          kkrnipcsyttpaqkiivfkprprsftvnsgcshnngmpe

BrMYB2-94S17.txt   ADIVPLCLGHNDTNNVSENIITCNKDDDKSELVSHLMDGQ   200
BrMYB2-11S91.txt   AGIVPLCLGHNDTNNVSENIITCNKDDDKSELVSHLMDGQ   200
Consensus          a ivplclghndtnnvseniitcnkdddkselvshlmdgq

BrMYB2-94S17.txt   NRWWESLLDESQDPAAIFPETTAIKKGATSAFDVEQLWSL   240
BrMYB2-11S91.txt   NRWWESLLDESQDPAAIFPETTAIKKGATSAFDVEQLWSL   240
Consensus          nrwwesllldesqdpaaifpettaikkgatsafdveqlwsl

BrMYB2-94S17.txt   LDGETG                                     246
BrMYB2-11S91.txt   LDGETG                                     246
Consensus          ldgetg
```

Figure S4 Electrophoresis results of *gBrMYB2* from different phenotypes of Chinese cabbage and co-segregation analysis using markers 'BrP1' and 'BrP2' in the F_2 population. **a** Electrophoresis results of *gBrMYB2* from purple head Chinese cabbage '11S91', and its female parent '94S17' and male parent '95T2-5'; **b** electrophoresis results of *gBrMYB2* from different phenotypes of Chinese cabbage; **c** co-segregation analysis using marker 'BrP1' in the F_2 population; **d** co-segregation analysis using marker 'BrP2' in the F_2 population. W: white head Chinese cabbage; P: purple head Chinese cabbage; H: hybrid Chinese cabbage. M1: DL2000 Marker; M2: DL10000 Marker; M3: DL5000 Marker.

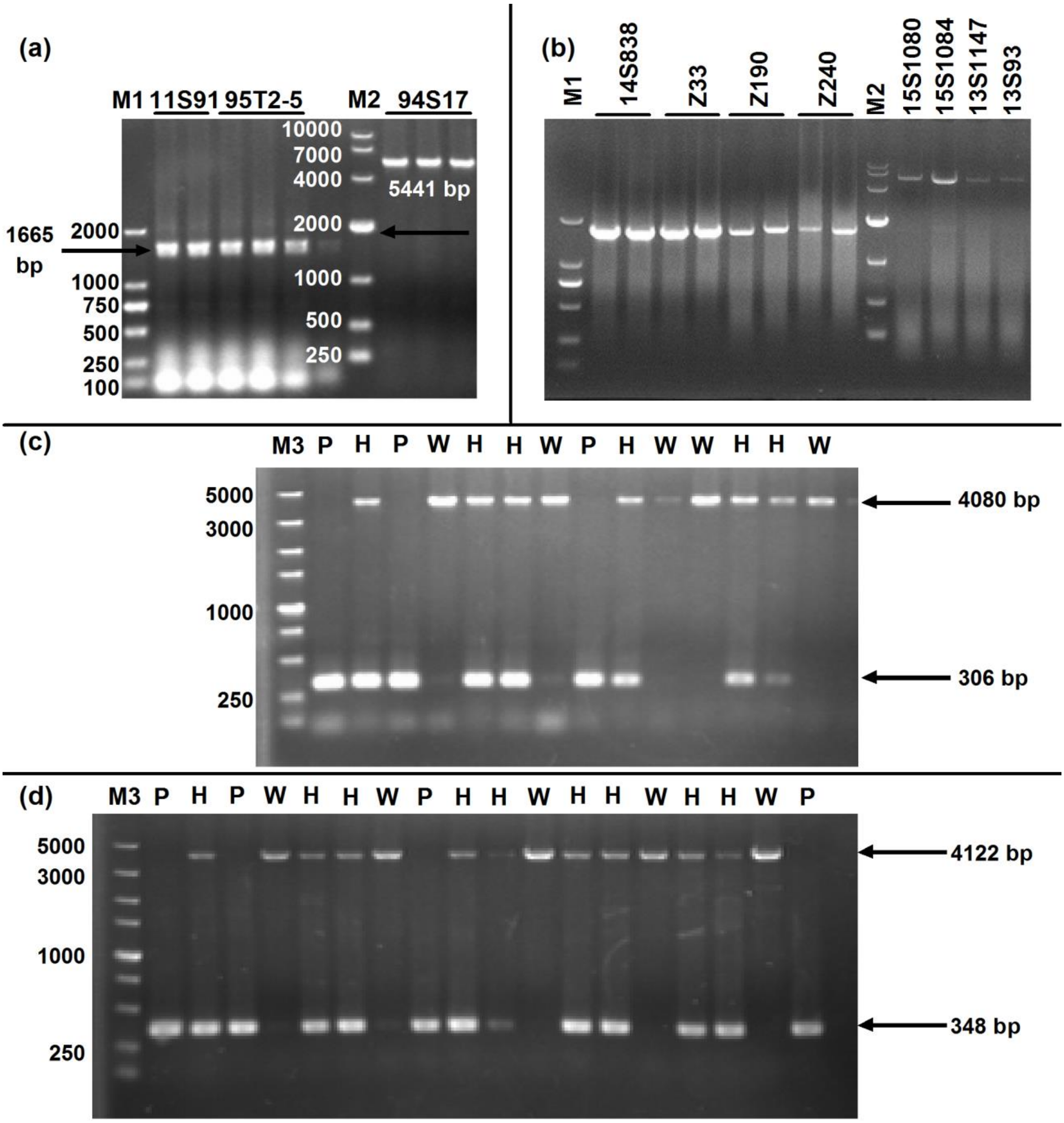

Figure S5 Multiple sequence alignment analysis of MYBs in different *Crucifer* species. At: *Arabidopsis*; Bo: *B. oleracea*; Br: *B. rapa*; Rs: *Raphanus sativus*. Arrowheads indicate differential proteins produced by SNPs between purple head Chinese cabbage '11S91' and its female parent '94S17'.

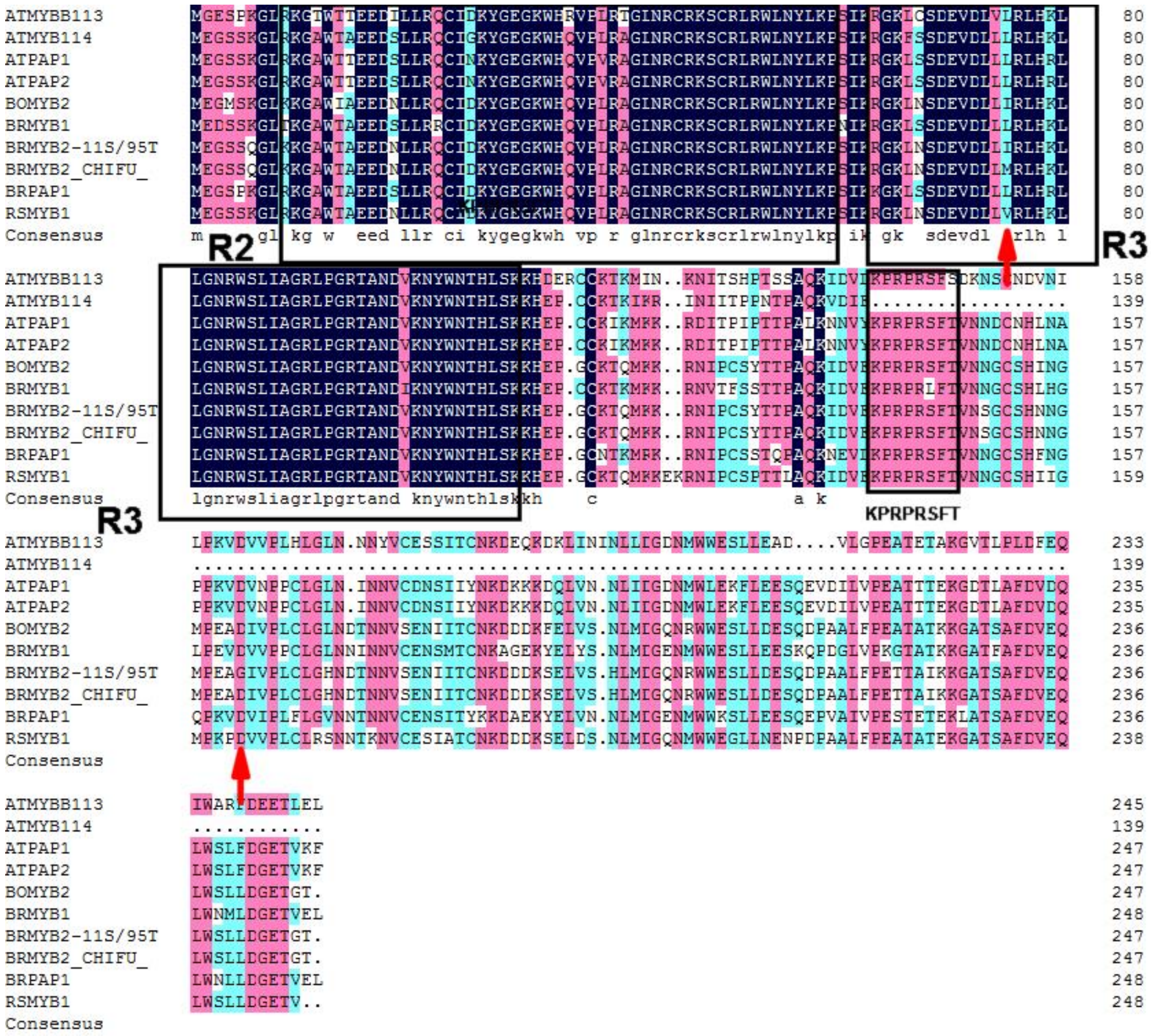

Figure S6 Multiple sequence alignment analysis of MYBs in different *Solanaceae* species.

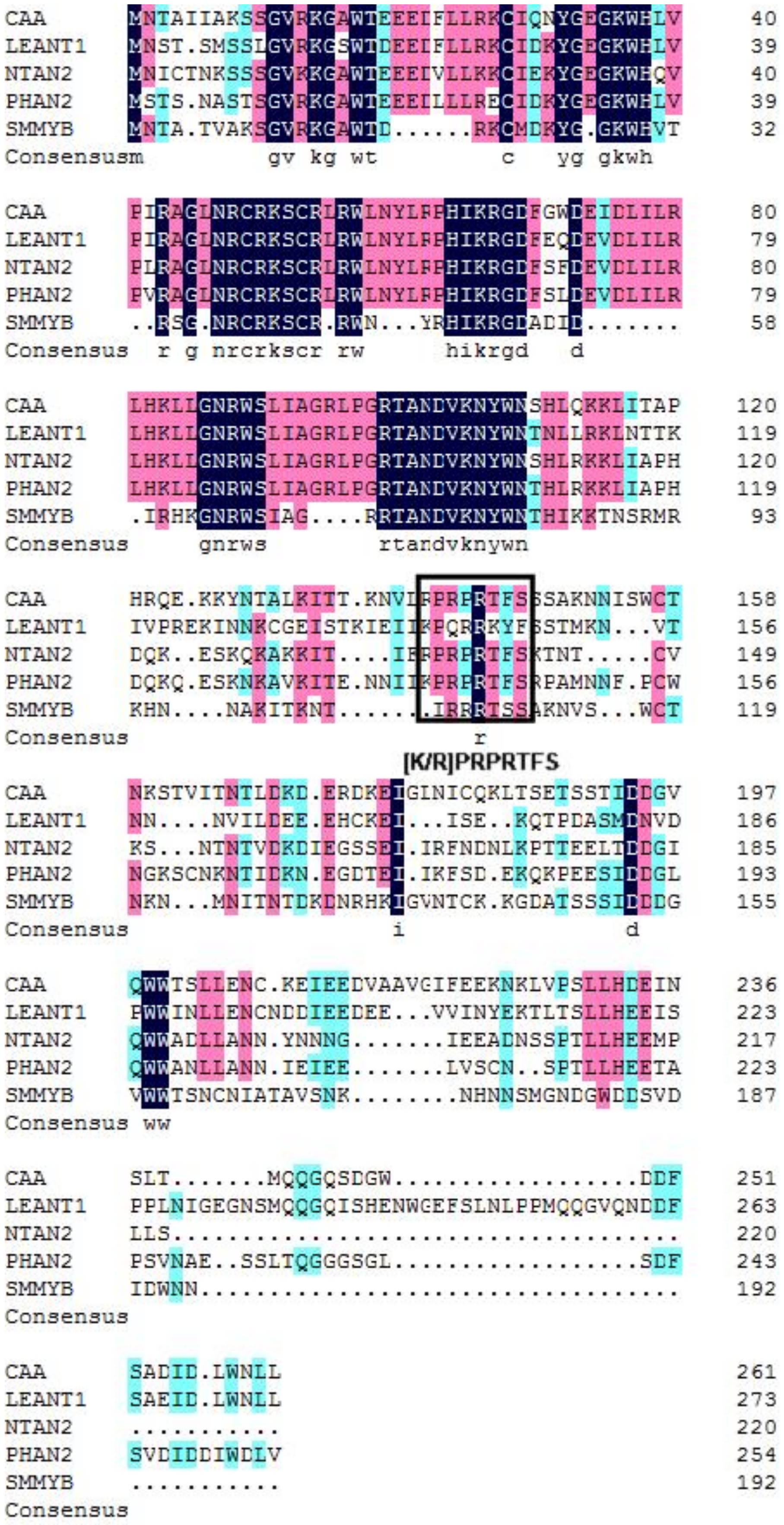

Figure S7 Prediction of elements of *BrMYB2* promoter in 11S91. Sequence marked with different color was in accordance with element showing in figure statement, and figure mainly showed results at the upstream region of 1500 bp started from ATG translation start site.

```
>userseq1980 1499nt
+ CTAAGTTTGA AAATCTGCAG ATGGATGAGA ATTAATCATT TGAGGAACTC AGTGGAAAAC TGAGTGCAAC
- GATTCAAACT TTTAGACGTC TACCTACTCT TAATTAGTAA ACTCCTTGAG TCACCTTTTG ACTCACGTTG

+ AGCAAATGAA GCACAAACAA TGGGAAAGAC ATACAAAGAC AAAAGGCTTG GAAGAAACTT CTCAGATGTT
- TCGTTTACTT CGTGTTTGTT ACCCTTTCTG TATGTTTCTG TTTTCCGAAC CTTCTTTGAA GAGTCTACAA

+ TACCCTCAAA GTTAAATCCA AGAAGACAGC TATGGACACT GCATTAGACA CTGACACAAT GGATTTTGGT
- ATGGGAGTTT CAATTTAGGT TCTTCTGTCG ATACCTGTGA CGTAATCTGT GACTGTGTTA CCTAAAACCA

+ GAATTCACGG GTCACTTACA AGCATATGAG ATGAATGAGA AACTCAAGGA AGAACTGTCT AAAAAAATCT
- CTTAAGTGCC CAGTGAATGT TCGTATACTC TACTTACTCT TTGAGTTCCT TCTTGACAGA TTTTTTTAGA

+ GAAACTAGAA AGCAGATTCA CATGCTGAAT AGTGGAACCA AGGATCTGGA GAAGATTCTC TCTGTTAGAA
- CTTTGATCTT TCGTCTAAGT GTACGACTTA TCACCTTGGT TCCTAGACCT CTTCTAAGAG AGACAATCTT

+ GGGTAGGCAA GTCCAACTTT GTGTCTCGGT TACAGTGGAA AACTACAGTA AAACAGAGTT TGTTCCGGCT
- CCCATCCGTT CAGGTTGAAA CACAGAGCCA ATGTCACCTT TTGATGTCAT TTTGTCTCAA ACAAGGCCGA

+ AAGACTTAAG AGATTGCGTC TGATACTACA GGAAGTAAGA TATCAACCAA GATCATCTAT TGTGAAACTG
- TTCTGAATTC TCTAACGCAG ACTATGATGT CCTTCATTCT ATAGTTGGTT CTAGTAGATA ACACTTTGAC

+ AAACCAGTGA GAACTATAAT GTAGGCTACT TCTGTGGTAA ATACATGCAC ATACCAAGAT ATACTTACAA
- TTTGGTCACT CTTGATATTA CATCCGATGA AGACACCATT TATGTACGTG TATGGTTCTA TATGAATGTT

+ ATTTCAAAAA AGAATCACTC CAGAGTTGAC TTTGCTTTCC AATCTTTACA GGTTTAACGT TAAATATGAA
- TAAAGTTTTT TCTTAGTGAG GTCTCAACTG AAACGAAAGG TTAGAAATGT CCAAATTGCA ATTTATACTT

+ ATTTGTAGTC AAACGATTAT GATGGATTTC ATCAAATCTT ATTGTTTTAA TATCAAATGA GGTTCAAGTT
- TAAACATCAG TTTGCTAATA CTACCTAAAG TAGTTTAGAA TAACAAAATT ATAGTTTACT CCAAGTTCAA

+ ACATATTTCT GAACTCCCTA GCCAAAAGTA CATACTCACA ATGTGAGACA CTTTAAATAC GGAACAATTT
- TGTATAAAGA CTTGAGGGAT CGGTTTTCAT GTATGAGTGT TACACTCTGT GAAATTTATG CCTTGTTAAA
```

```
+ GCAAAATAGT GATTTGTGTA CTCATAATTA TTGTAGCTGA TTTTTGTCTA CATACACTTG TTGACAGAAA
- CGTTTTATCA CTAAACACAT GAGTATTAAT AACATCGACT AAAAACAGAT GTATGTGAAC AACTGTCTTT

+ AACAATACGC TACTTATCAA GTTGTGTACT CATACATCAA CCCTCTGTCT CTCTCTCTCT CTCTCTCTCT
- TTGTTATGCG ATGAATAGTT CAACACATGA GTATGTAGTT GGGAGACAGA GAGAGAGAGA GAGAGAGAGA

+ CTCTCTCTCT CTCTCTCTCT CTCTCTCTCT CTCTCTCTCT CTCTCTCTCT CTCTCTCTCT CTCTCTCTCT
- GAGAGAGAGA GAGAGAGAGA GAGAGAGAGA GAGAGAGAGA GAGAGAGAGA GAGAGAGAGA GAGAGAGAGA

+ CTCTCTCTCT CTCTCTCTCT CTATTCTGTT TTTAACTCTG GTGCATGAAG CTGTTCTCTC TTTTAATCAT
- GAGAGAGAGA GAGAGAGAGA GATAAGACAA AAATTGAGAC CACGTACTTC GACAAGAGAG AAAATTAGTA

+ ATAAACCACC TTTCCACCAA TCATTAATCG TCCTTTCTGC TAACTTTGCT AAGAGGCATG TTTTTCCTTT
- TATTTGGTGG AAAGGTGGTT AGTAATTAGC AGGAAAGACG ATTGAAACGA TTCTCCGTAC AAAAAGGAAA

+ AAAGTACTGA AAAATATGAA GAGACTAAGT GATATGCTAT GCTATGACGA AAATGGACTA AGTGCTATGA
- TTTCATGACT TTTTATACTT CTCTGATTCA CTATACGATA CGATACTGCT TTTACCTGAT TCACGATACT

+ ACCTTTTCTG TATTTAGTTT GAATCAGTAG CAGAACATAA ATAACTAAAT TAGCAAATTA GACACGACAA
- TGGAAAAGAC ATAAATCAAA CTTAGTCATC GTCTTGTATT TATTGATTTA ATCGTTTAAT CTGTGCTGTT

+ AATGTGCTAT GAACCTTTTT TGTTTCTGCA TCGTACCCTG GAAGTTGTTA AAAAAAAAGA CACGACAAAA
- TTACACGATA CTTGGAAAAA ACAAAGACGT AGCATGGGAC CTTCAACAAT TTTTTTTTCT GTGCTGTTTT

+ ATGTGGATGT TAGACATGCA CGTCACTTCT CTTTGCCCGT CACGTGTATA AATAAAGTCC TCGAAGGATT
- TACACCTACA ATCTGTACGT GCAGTGAAGA GAAACGGGCA GTGCACATAT TTATTTCAGG AGCTTCCTAA

+ TGTCAAGCCA CAATAGAAAC CCTTTTCCTC AAGCCTGCCT TTACGTATAA CTTTTTAAAA TAAAATTTGG
- ACAGTTCGGT GTTATCTTTG GGAAAAGGAG TTCGGACGGA AATGCATATT GAAAAATTTT ATTTTAAACC

+ TTAGATACTT CTAAAATTAT AGCTGGTCC
- AATCTATGAA GATTTTAATA TCGACCAGG
```

- + 5UTR Py-rich stretch
- + ABRE
- + AE-box
- + ARE
- + Box 4
- + Box I
- + CAAT-box
- + CGTCA-motif
- + CTAG-motif
- + ERE
- + G-Box
- + G-box
- + GA-motif
- + [illegible]

Figure S8 Alignment analysis of *BrMYB2* promoter in different Chinese cabbages. Results showed the different CT repeat region.

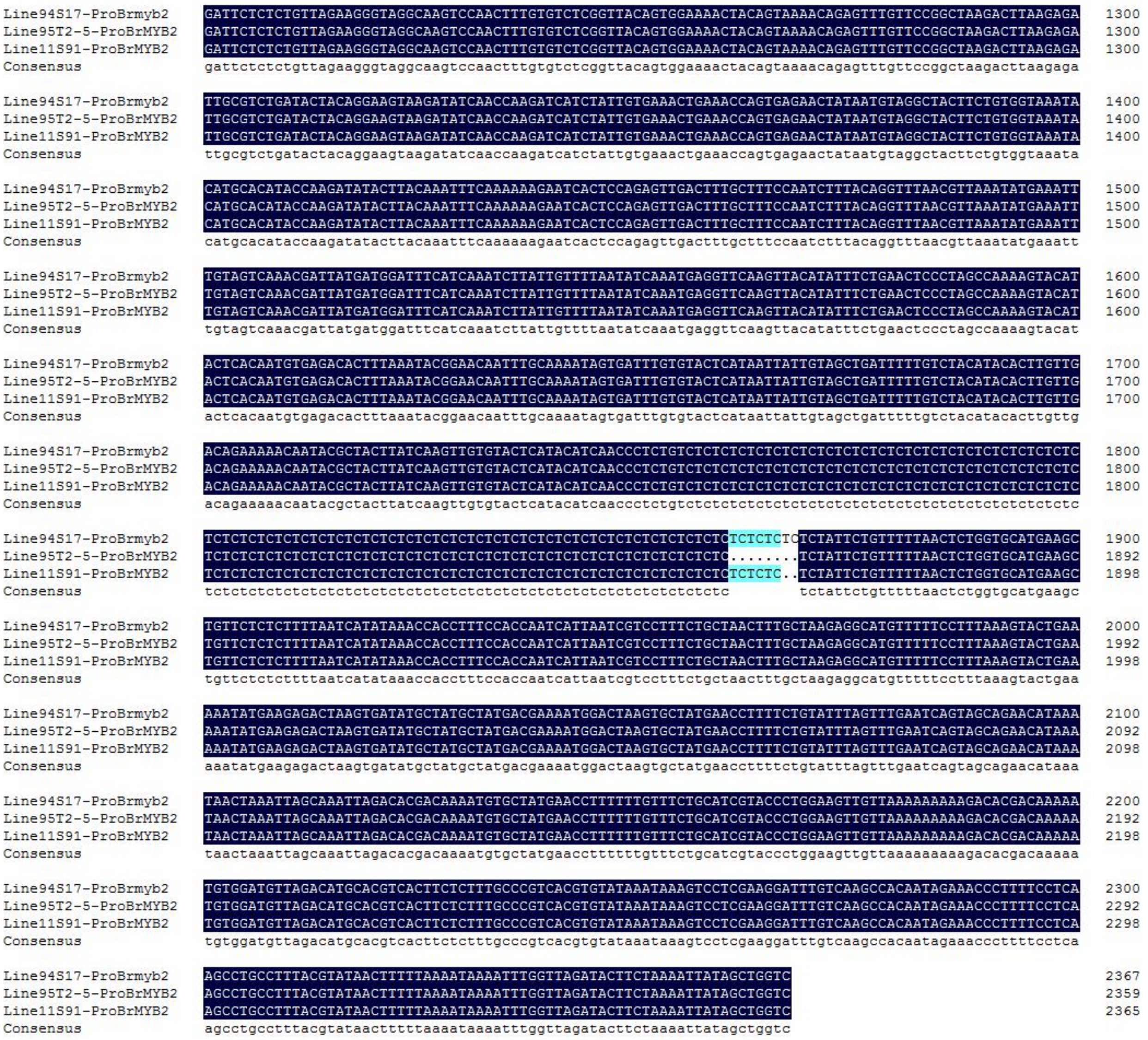

Figure S9 Results of PCR screening on T_1 and T_3 homozygous transgenic *Arabidopsis*. **a** T_1 CaMV35S:*cBrMYB2* lines detected by *BrMYB2* clone primer; **b** T_1 CaMV35S:*cBrmyb2* lines detected by *BrMYB2* clone primer; **c** T_3 transgenic lines detected by *BrMYB2* clone primer. M: DL2000 Marker.

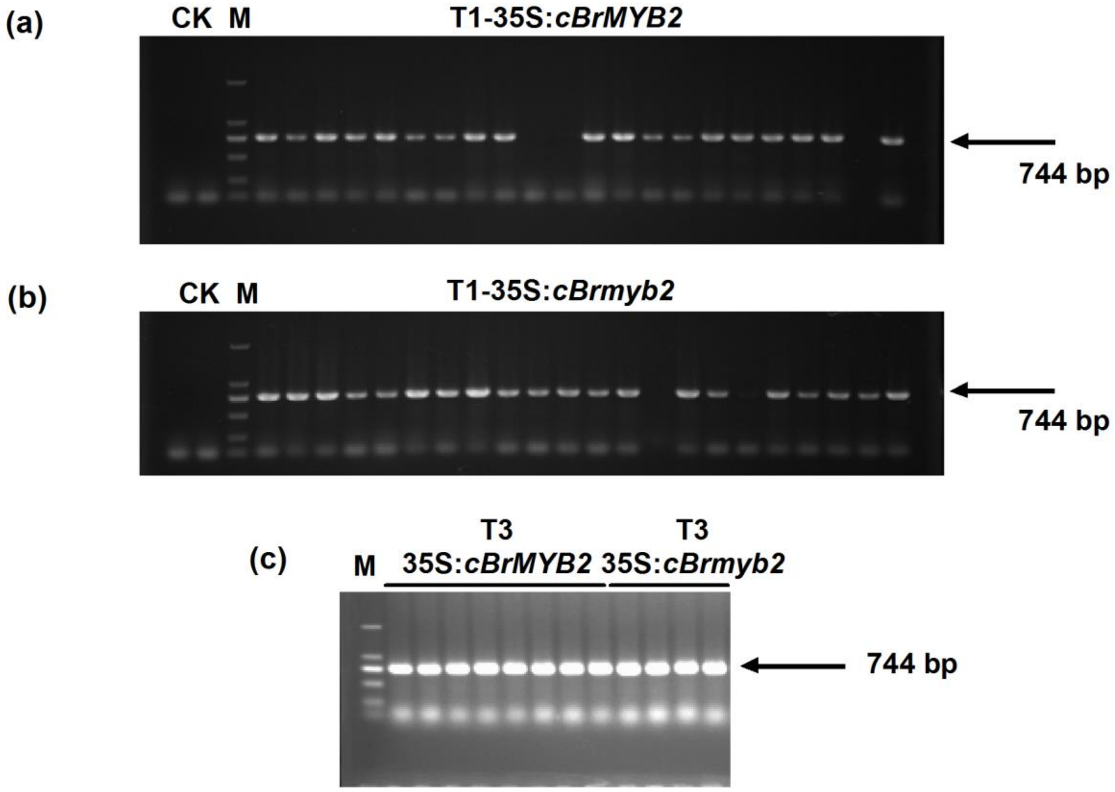

Figure S10 Identification of T_1 and T_3 homozygous transgenic *Arabidopsis* on MS solid medium containing 50mg·L^{-1} kanamycin (Kan). **a** WT *Arabidopsis* without Kan; **b** WT *Arabidopsis* with Kan; **c** T_1 CaMV35S:*cBrMYB2* lines with Kan; **d** T_1 CaMV35S:*cBrmyb2* lines with Kan; **e-k** T_3 CaMV35S:*cBrMYB2* lines with Kan; **l-o** T_3 CaMV:*cBrmyb2* lines with Kan. Scale bar is 1.2 cm.

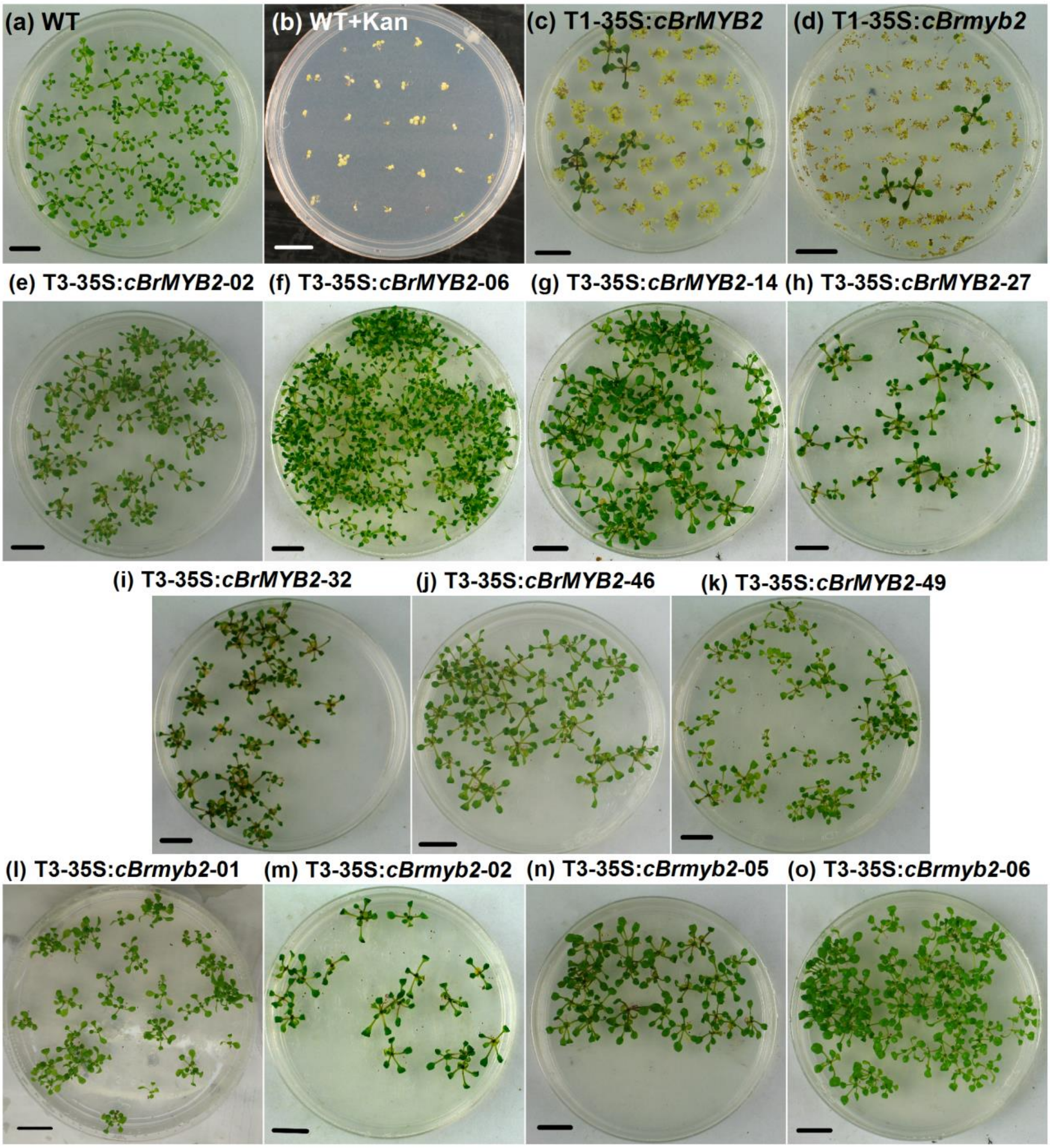

Figure S11 Gene expression patterns of anthocyanin biosynthesis in T_3 CaMV35S:c*BrMYB2* *Arabidopsis*. **a** WT *Arabidopsis*; **b-h** seven different CaMV35S:c*BrMYB2* lines; samples were 50-day *Arabidopsis* seedlings; **i** qRT-PCR and semi-qRT-PCR analysis of *BrMYB2*, and Line06 of T_3 *cBrMYB2* lines served as the control. **j-ab** Expression patterns of ABGs in *Arabidopsis*, and WT *Arabidopsis* served as the control in data analysis. Values are presented as means ± SD (n = 3). The different letters above each column are significantly different at $p < 0.05$ by Duncan's test. The scale bar is 2 cm.

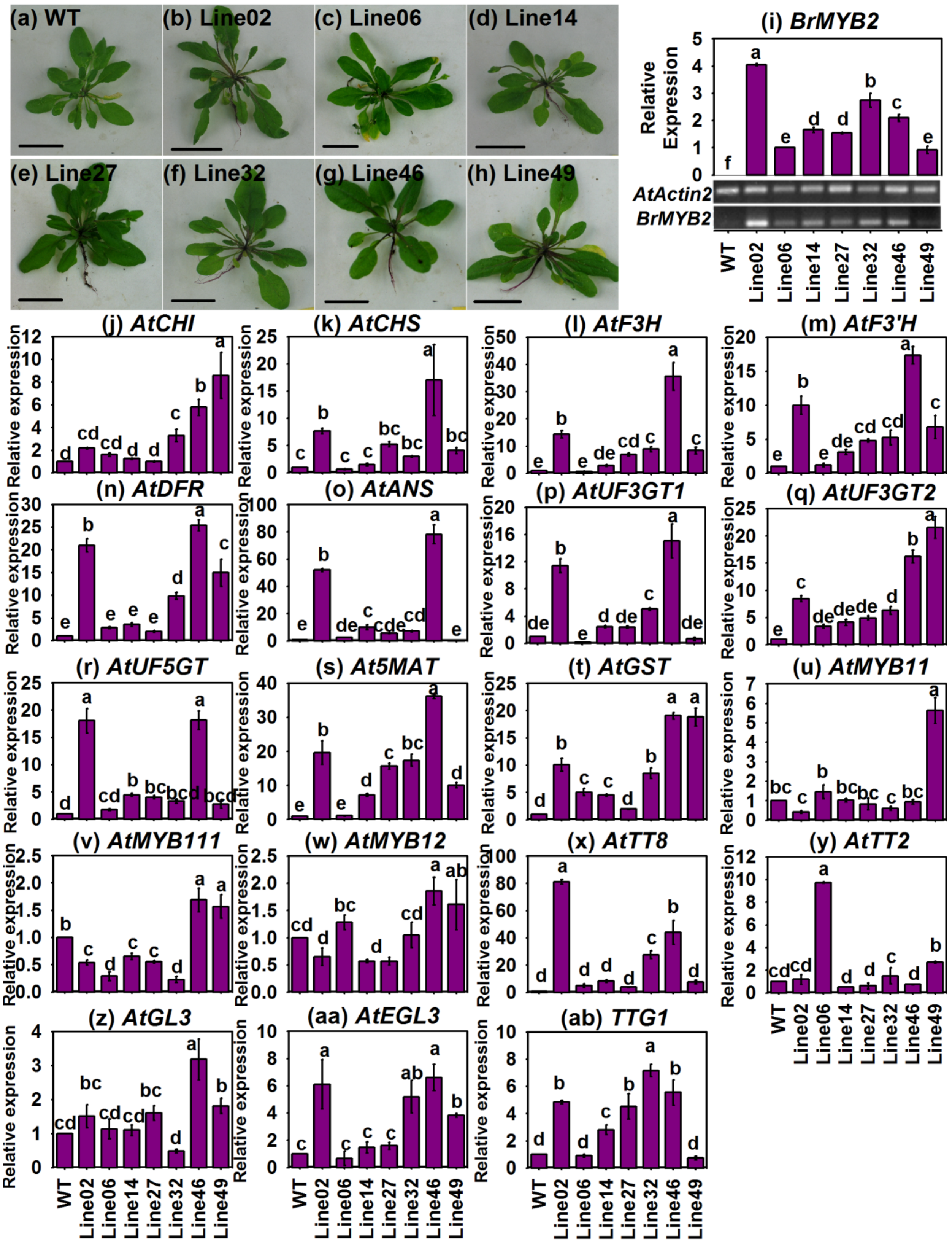

(a) WT
(b) Line02
(c) Line06
(d) Line14
(e) Line27
(f) Line32
(g) Line46
(h) Line49
(i) BrMYB2
Relative Expression
AtActin2
BrMYB2
WT
Line02
Line06
Line14
Line27
Line32
Line46
Line49
(j) AtCHI
(k) AtCHS
(l) AtF3H
(m) AtF3'H
(n) AtDFR
(o) AtANS
(p) AtUF3GT1
(q) AtUF3GT2
(r) AtUF5GT
(s) At5MAT
(t) AtGST
(u) AtMYB11
(v) AtMYB111
(w) AtMYB12
(x) AtTT8
(y) AtTT2
(z) AtGL3
(aa) AtEGL3
(ab) TTG1
Relative expression

Figure S12 Results of PCR detecting on T_1 and T_3 homozygous transgenic *Arabidopsis* using *BrMYB2* clone primer. **a** T_1 CaMV35S:*gBrMYB2* lines; **b** T_1 CaMV35S:*gBrmyb2* lines; **c** T_3 of CaMV35S:*gBrMYB2* transgenic *Arabidopsis*; **d** T_3 of CaMV35S:*gBrmyb2* transgenic *Arabidopsis*, M1: DL2000 Marker; M2: DL10000 Marker.

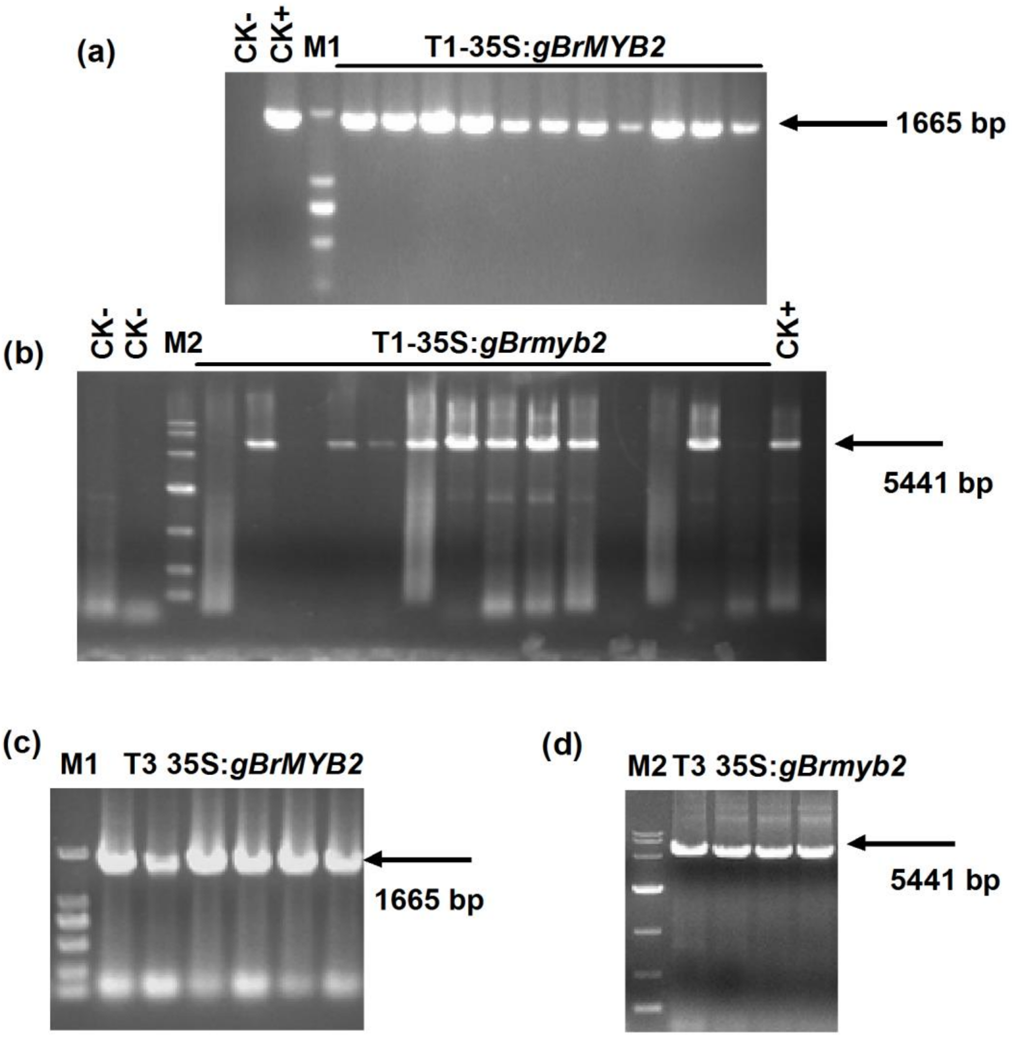

Figure S13 Identification of T_1 and T_3 homozygous transgenic *Arabidopsis* on MS solid medium containing 50mg·L^{-1} Kan. **a** WT *Arabidopsis* without Kan; **b** WT *Arabidopsis* with Kan; **c** T_1 CaMV35S:*gBrMYB2* lines with Kan; **d** T_1 CaMV35S:*gBrmyb2* lines with Kan; **e** 50-day WT *Arabidopsis*; **f** 50-day T_1 CaMV35S:*cBrMYB2* lines; **g** 50-day T_1 CaMV35S:*gBrmyb2* lines; **h** 50-day T_1 CaMV35S:*gBrMYB2* lines. **i-k** T_3 CaMV35S:*gBrMYB2* lines with Kan; **l-n** T_3 CaMV35S:*gBrmyb2* lines with Kan. Scale bar is 1.2 cm.

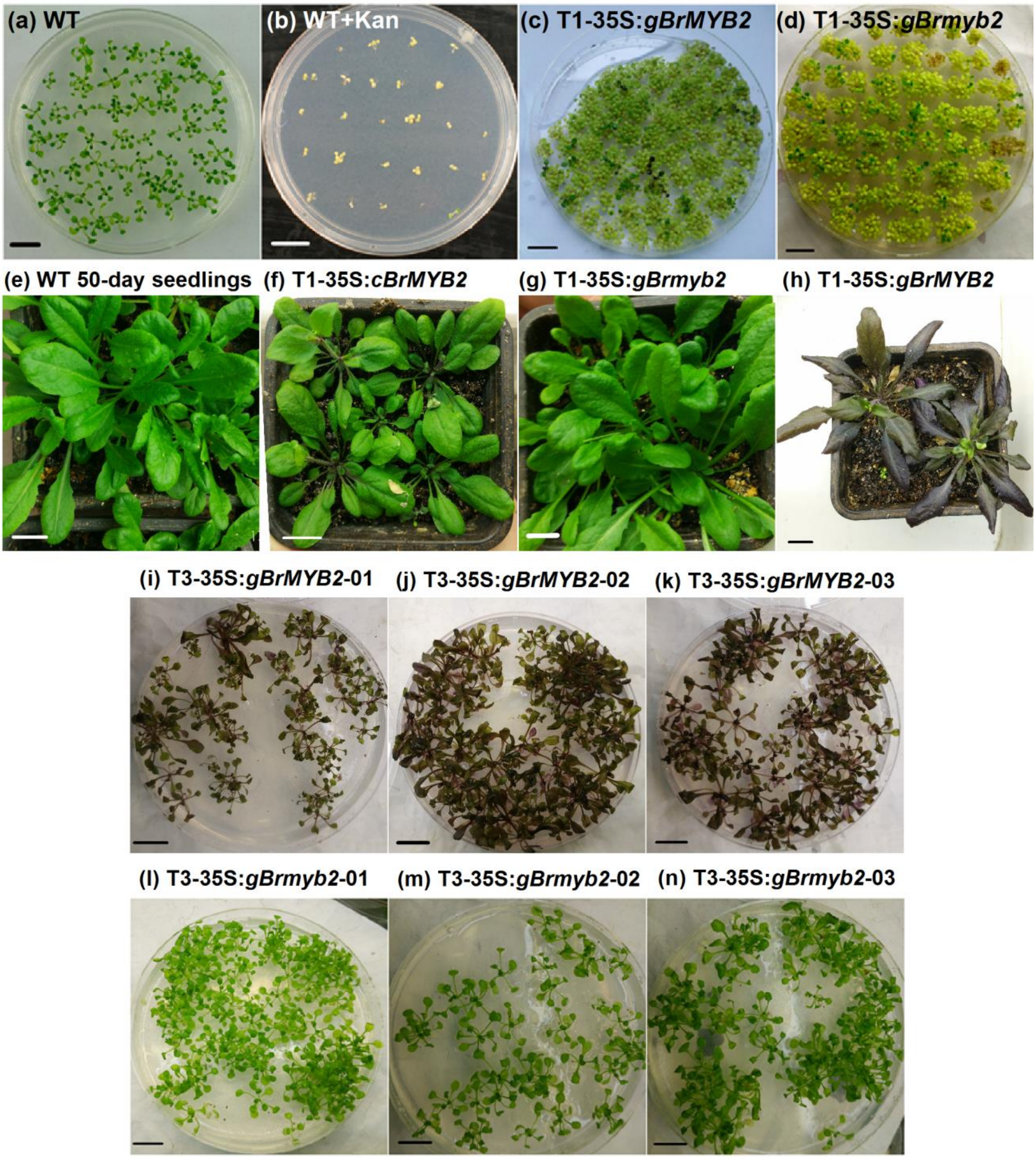

Figure S14 Sequence alignment analysis of identified *BrMYB2* and *Brmyb2* using reverse transcription-PCR in transgenic *Arabidopsis* lines.

```
T3-cBrmyb2-1    ATGGAGGGTTCGTCCCAAGGGTTGAAAAAAGGTGCATGGACTGCTGAAGAAGATAATCTCTTGAGGCAATGCATTGATAAGTATGGAGAAGGGAAATGGC   100
T3-cBrmyb2-2    ATGGAGGGTTCGTCCCAAGGGTTGAAAAAAGGTGCATGGACTGCTGAAGAAGATAATCTCTTGAGGCAATGCATTGATAAGTATGGAGAAGGGAAATGGC   100
T3-cBrmyb2-3    ATGGAGGGTTCGTCCCAAGGGTTGAAAAAAGGTGCATGGACTGCTGAAGAAGATAATCTCTTGAGGCAATGCATTGATAAGTATGGAGAAGGGAAATGGC   100
T3-cBrMYB2-1    ATGGAGGGTTCGTCCCAAGGGTTGAAAAAAGGTGCATGGACTGCTGAAGAAGATAATCTCTTGAGGCAATGCATTGATAAGTATGGAGAAGGGAAATGGC   100
T3-cBrMYB2-2    ATGGAGGGTTCGTCCCAAGGGTTGAAAAAAGGTGCATGGACTGCTGAAGAAGATAATCTCTTGAGGCAATGCATTGATAAGTATGGAGAAGGGAAATGGC   100
T3-cBrMYB2-3    ATGGAGGGTTCGTCCCAAGGGTTGAAAAAAGGTGCATGGACTGCTGAAGAAGATAATCTCTTGAGGCAATGCATTGATAAGTATGGAGAAGGGAAATGGC   100
T3-TgBrMYB2-1   ATGGAGGGTTCGTCCCAAGGGTTGAAAAAAGGTGCATGGACTGCTGAAGAAGATAATCTCTTGAGGCAATGCATTGATAAGTATGGAGAAGGGAAATGGC   100
T3-TgBrMYB2-1   ATGGAGGGTTCGTCCCAAGGGTTGAAAAAAGGTGCATGGACTGCTGAAGAAGATAATCTCTTGAGGCAATGCATTGATAAGTATGGAGAAGGGAAATGGC   100
T3-TgBrMYB2-1   ATGGAGGGTTCGTCCCAAGGGTTGAAAAAAGGTGCATGGACTGCTGAAGAAGATAATCTCTTGAGGCAATGCATTGATAAGTATGGAGAAGGGAAATGGC   100
Consensus       atggagggttcgtcccaagggttgaaaaaaggtgcatggactgctgaagaagataatctcttgaggcaatgcattgataagtatggagaagggaaatggc

T3-cBrmyb2-1    ACCAAGTTCCTTTAAGAGCTGGTCTAAATCGGTGCAGGAAGAGTTGTAGACTAAGATGGTTGAACTATTTGAAGCCAAGTATCAAGAGAGGAAAACTCAA   200
T3-cBrmyb2-2    ACCAAGTTCCTTTAAGAGCTGGTCTAAATCGGTGCAGGAAGAGTTGTAGACTAAGATGGTTGAACTATTTGAAGCCAAGTATCAAGAGAGGAAAACTCAA   200
T3-cBrmyb2-3    ACCAAGTTCCTTTAAGAGCTGGTCTAAATCGGTGCAGGAAGAGTTGTAGACTAAGATGGTTGAACTATTTGAAGCCAAGTATCAAGAGAGGAAAACTCAA   200
T3-cBrMYB2-1    ACCAAGTTCCTTTAAGAGCTGGTCTAAATCGGTGCAGGAAGAGTTGTAGACTAAGATGGTTGAACTATTTGAAGCCAAGTATCAAGAGAGGAAAACTCAA   200
T3-cBrMYB2-2    ACCAAGTTCCTTTAAGAGCTGGTCTAAATCGGTGCAGGAAGAGTTGTAGACTAAGATGGTTGAACTATTTGAAGCCAAGTATCAAGAGAGGAAAACTCAA   200
T3-cBrMYB2-3    ACCAAGTTCCTTTAAGAGCTGGTCTAAATCGGTGCAGGAAGAGTTGTAGACTAAGATGGTTGAACTATTTGAAGCCAAGTATCAAGAGAGGAAAACTCAA   200
T3-TgBrMYB2-1   ACCAAGTTCCTTTAAGAGCTGGTCTAAATCGGTGCAGGAAGAGTTGTAGACTAAGATGGTTGAACTATTTGAAGCCAAGTATCAAGAGAGGAAAACTCAA   200
T3-TgBrMYB2-1   ACCAAGTTCCTTTAAGAGCTGGTCTAAATCGGTGCAGGAAGAGTTGTAGACTAAGATGGTTGAACTATTTGAAGCCAAGTATCAAGAGAGGAAAACTCAA   200
T3-TgBrMYB2-1   ACCAAGTTCCTTTAAGAGCTGGTCTAAATCGGTGCAGGAAGAGTTGTAGACTAAGATGGTTGAACTATTTGAAGCCAAGTATCAAGAGAGGAAAACTCAA   200
Consensus       accaagttcctttaagagctggtctaaatcggtgcaggaagagttgtagactaagatggttgaactatttgaagccaagtatcaagagaggaaaactcaa

T3-cBrmyb2-1    CTCCGATGAAGTTGATCTTCTTATGCGCCTTCATAAGCTTTTAGGAAACAGGTGGTCTTTAATTGCTGGTAGATTACCCGGTCGGACCGCCAATGACGTC   300
T3-cBrmyb2-2    CTCCGATGAAGTTGATCTTCTTATGCGCCTTCATAAGCTTTTAGGAAACAGGTGGTCTTTAATTGCTGGTAGATTACCCGGTCGGACCGCCAATGACGTC   300
T3-cBrmyb2-3    CTCCGATGAAGTTGATCTTCTTATGCGCCTTCATAAGCTTTTAGGAAACAGGTGGTCTTTAATTGCTGGTAGATTACCCGGTCGGACCGCCAATGACGTC   300
T3-cBrMYB2-1    CTCCGATGAAGTTGATCTTCTTATTCGCCTTCATAAGCTTTTAGGAAACAGGTGGTCTTTAATTGCTGGTAGATTACCCGGTCGGACCGCCAATGACGTC   300
T3-cBrMYB2-2    CTCCGATGAAGTTGATCTTCTTATTCGCCTTCATAAGCTTTTAGGAAACAGGTGGTCTTTAATTGCTGGTAGATTACCCGGTCGGACCGCCAATGACGTC   300
T3-cBrMYB2-3    CTCCGATGAAGTTGATCTTCTTATTCGCCTTCATAAGCTTTTAGGAAACAGGTGGTCTTTAATTGCTGGTAGATTACCCGGTCGGACCGCCAATGACGTC   300
T3-TgBrMYB2-1   CTCCGATGAAGTTGATCTTCTTATTCGCCTTCATAAGCTTTTAGGAAACAGGTGGTCTTTAATTGCTGGTAGATTACCCGGTCGGACCGCCAATGACGTC   300
T3-TgBrMYB2-1   CTCCGATGAAGTTGATCTTCTTATTCGCCTTCATAAGCTTTTAGGAAACAGGTGGTCTTTAATTGCTGGTAGATTACCCGGTCGGACCGCCAATGACGTC   300
T3-TgBrMYB2-1   CTCCGATGAAGTTGATCTTCTTATTCGCCTTCATAAGCTTTTAGGAAACAGGTGGTCTTTAATTGCTGGTAGATTACCCGGTCGGACCGCCAATGACGTC   300
Consensus       ctccgatgaagttgatcttcttat cgccttcataagcttttaggaaacaggtggtctttaattgctggtagattacccggtcggaccgccaatgacgtc

T3-cBrmyb2-1    AAAAATTACTGGAACACCCATTTGAGTAAGAAACATGAACCGGGTTGTAAGACCCAGATGAAAAAGAGAAACATTCCTTGCTCTTATACCACACCAGCCC   400
T3-cBrmyb2-2    AAAAATTACTGGAACACCCATTTGAGTAAGAAACATGAACCGGGTTGTAAGACCCAGATGAAAAAGAGAAACATTCCTTGCTCTTATACCACACCAGCCC   400
T3-cBrmyb2-3    AAAAATTACTGGAACACCCATTTGAGTAAGAAACATGAACCGGGTTGTAAGACCCAGATGAAAAAGAGAAACATTCCTTGCTCTTATACCACACCAGCCC   400
T3-cBrMYB2-1    AAAAATTACTGGAACACCCATTTGAGTAAGAAACATGAACCGGGTTGTAAGACCCAGATGAAAAAGAGAAACATTCCTTGCTCTTATACCACACCAGCCC   400
T3-cBrMYB2-2    AAAAATTACTGGAACACCCATTTGAGTAAGAAACATGAACCGGGTTGTAAGACCCAGATGAAAAAGAGAAACATTCCTTGCTCTTATACCACACCAGCCC   400
T3-cBrMYB2-3    AAAAATTACTGGAACACCCATTTGAGTAAGAAACATGAACCGGGTTGTAAGACCCAGATGAAAAAGAGAAACATTCCTTGCTCTTATACCACACCAGCCC   400
T3-TgBrMYB2-1   AAAAATTACTGGAACACCCATTTGAGTAAGAAACATGAACCGGGTTGTAAGACCCAGATGAAAAAGAGAAACATTCCTTGCTCTTATACCACACCAGCCC   400
T3-TgBrMYB2-1   AAAAATTACTGGAACACCCATTTGAGTAAGAAACATGAACCGGGTTGTAAGACCCAGATGAAAAAGAGAAACATTCCTTGCTCTTATACCACACCAGCCC   400
T3-TgBrMYB2-1   AAAAATTACTGGAACACCCATTTGAGTAAGAAACATGAACCGGGTTGTAAGACCCAGATGAAAAAGAGAAACATTCCTTGCTCTTATACCACACCAGCCC   400
Consensus       aaaaattactggaacacccatttgagtaagaaacatgaaccgggttgtaagacccagatgaaaaagagaaacattccttgctcttataccacaccagccc

T3-cBrmyb2-1    AAAAAATCGACGTTTTCAAACCTCGACCTCGATCCTTCACCGTTAACAGCGGCTGCAGCCATAATAATGGCATGCCAGAAGCTGACATTGTTCCTCTATG   500
T3-cBrmyb2-2    AAAAAATCGACGTTTTCAAACCTCGACCTCGATCCTTCACCGTTAACAGCGGCTGCAGCCATAATAATGGCATGCCAGAAGCTGACATTGTTCCTCTATG   500
T3-cBrmyb2-3    AAAAAATCGACGTTTTCAAACCTCGACCTCGATCCTTCACCGTTAACAGCGGCTGCAGCCATAATAATGGCATGCCAGAAGCTGACATTGTTCCTCTATG   500
T3-cBrMYB2-1    AAAAAATCGACGTTTTCAAACCTCGACCTCGATCCTTCACCGTTAACAGCGGCTGCAGCCATAATAATGGCATGCCAGAAGCTGGCATTGTTCCTCTATG   500
T3-cBrMYB2-2    AAAAAATCGACGTTTTCAAACCTCGACCTCGATCCTTCACCGTTAACAGCGGCTGCAGCCATAATAATGGCATGCCAGAAGCTGGCATTGTTCCTCTATG   500
T3-cBrMYB2-3    AAAAAATCGACGTTTTCAAACCTCGACCTCGATCCTTCACCGTTAACAGCGGCTGCAGCCATAATAATGGCATGCCAGAAGCTGGCATTGTTCCTCTATG   500
T3-TgBrMYB2-1   AAAAAATCGACGTTTTCAAACCTCGACCTCGATCCTTCACCGTTAACAGCGGCTGCAGCCATAATAATGGCATGCCAGAAGCTGGCATTGTTCCTCTATG   500
T3-TgBrMYB2-1   AAAAAATCGACGTTTTCAAACCTCGACCTCGATCCTTCACCGTTAACAGCGGCTGCAGCCATAATAATGGCATGCCAGAAGCTGGCATTGTTCCTCTATG   500
T3-TgBrMYB2-1   AAAAAATCGACGTTTTCAAACCTCGACCTCGATCCTTCACCGTTAACAGCGGCTGCAGCCATAATAATGGCATGCCAGAAGCTGGCATTGTTCCTCTATG   500
Consensus       aaaaaatcgacgttttcaaacctcgacctcgatccttcaccgttaacagcggctgcagccataataatggcatgccagaagctg cattgttcctctatg

T3-cBrmyb2-1    CCTTGGACACAACGATACTAATAATGTTTCTGAAAATATAATCACATGTAACAAAGATGATGATAAATCTGAGCTTGTTAGTCATTTAATGGATGGTCAG   600
T3-cBrmyb2-2    CCTTGGACACAACGATACTAATAATGTTTCTGAAAATATAATCACATGTAACAAAGATGATGATAAATCTGAGCTTGTTAGTCATTTAATGGATGGTCAG   600
T3-cBrmyb2-3    CCTTGGACACAACGATACTAATAATGTTTCTGAAAATATAATCACATGTAACAAAGATGATGATAAATCTGAGCTTGTTAGTCATTTAATGGATGGTCAG   600
T3-cBrMYB2-1    CCTTGGACACAACGATACTAATAATGTTTCTGAAAATATAATCACATGTAACAAAGATGATGATAAATCTGAGCTTGTTAGTCATTTAATGGATGGTCAG   600
T3-cBrMYB2-2    CCTTGGACACAACGATACTAATAATGTTTCTGAAAATATAATCACATGTAACAAAGATGATGATAAATCTGAGCTTGTTAGTCATTTAATGGATGGTCAG   600
T3-cBrMYB2-3    CCTTGGACACAACGATACTAATAATGTTTCTGAAAATATAATCACATGTAACAAAGATGATGATAAATCTGAGCTTGTTAGTCATTTAATGGATGGTCAG   600
T3-TgBrMYB2-1   CCTTGGACACAACGATACTAATAATGTTTCTGAAAATATAATCACATGTAACAAAGATGATGATAAATCTGAGCTTGTTAGTCATTTAATGGATGGTCAG   600
T3-TgBrMYB2-1   CCTTGGACACAACGATACTAATAATGTTTCTGAAAATATAATCACATGTAACAAAGATGATGATAAATCTGAGCTTGTTAGTCATTTAATGGATGGTCAG   600
T3-TgBrMYB2-1   CCTTGGACACAACGATACTAATAATGTTTCTGAAAATATAATCACATGTAACAAAGATGATGATAAATCTGAGCTTGTTAGTCATTTAATGGATGGTCAG   600
Consensus       ccttggacacaacgatactaataatgtttctgaaaatataatcacatgtaacaaagatgatgataaatctgagcttgttagtcatttaatggatggtcag

T3-cBrmyb2-1    AATAGGTGGTGGGAAAGTTTGCTAGATGAGAGCCAAGATCCAGCTGCGCTCTTTCCAGAAACTACAGCAATAAAAAAGGGCGCAACCTCCGCGTTTGACG   700
T3-cBrmyb2-2    AATAGGTGGTGGGAAAGTTTGCTAGATGAGAGCCAAGATCCAGCTGCGCTCTTTCCAGAAACTACAGCAATAAAAAAGGGCGCAACCTCCGCGTTTGACG   700
T3-cBrmyb2-3    AATAGGTGGTGGGAAAGTTTGCTAGATGAGAGCCAAGATCCAGCTGCGCTCTTTCCAGAAACTACAGCAATAAAAAAGGGCGCAACCTCCGCGTTTGACG   700
T3-cBrMYB2-1    AATAGGTGGTGGGAAAGTTTGCTAGATGAGAGCCAAGATCCAGCTGCGCTCTTTCCAGAAACTACAGCAATAAAAAAGGGCGCAACCTCCGCGTTTGACG   700
T3-cBrMYB2-2    AATAGGTGGTGGGAAAGTTTGCTAGATGAGAGCCAAGATCCAGCTGCGCTCTTTCCAGAAACTACAGCAATAAAAAAGGGCGCAACCTCCGCGTTTGACG   700
T3-cBrMYB2-3    AATAGGTGGTGGGAAAGTTTGCTAGATGAGAGCCAAGATCCAGCTGCGCTCTTTCCAGAAACTACAGCAATAAAAAAGGGCGCAACCTCCGCGTTTGACG   700
T3-TgBrMYB2-1   AATAGGTGGTGGGAAAGTTTGCTAGATGAGAGCCAAGATCCAGCTGCGCTCTTTCCAGAAACTACAGCAATAAAAAAGGGCGCAACCTCCGCGTTTGACG   700
T3-TgBrMYB2-1   AATAGGTGGTGGGAAAGTTTGCTAGATGAGAGCCAAGATCCAGCTGCGCTCTTTCCAGAAACTACAGCAATAAAAAAGGGCGCAACCTCCGCGTTTGACG   700
T3-TgBrMYB2-1   AATAGGTGGTGGGAAAGTTTGCTAGATGAGAGCCAAGATCCAGCTGCGCTCTTTCCAGAAACTACAGCAATAAAAAAGGGCGCAACCTCCGCGTTTGACG   700
Consensus       aataggtggtgggaaagtttgctagatgagagccaagatccagctgcgctctttccagaaactacagcaataaaaaagggcgcaacctccgcgtttgacg

T3-cBrmyb2-1    TTGAGCAACTTTGGAGCCTGTTGGATGGAGAAACTGGAACTTG   743
T3-cBrmyb2-2    TTGAGCAACTTTGGAGCCTGTTGGATGGAGAAACTGGAACTTG   743
T3-cBrmyb2-3    TTGAGCAACTTTGGAGCCTGTTGGATGGAGAAACTGGAACTTG   743
T3-cBrMYB2-1    TTGAGCAACTTTGGAGCCTGTTGGATGGAGAAACTGGAACTTG   743
T3-cBrMYB2-2    TTGAGCAACTTTGGAGCCTGTTGGATGGAGAAACTGGAACTTG   743
T3-cBrMYB2-3    TTGAGCAACTTTGGAGCCTGTTGGATGGAGAAACTGGAACTTG   743
T3-TgBrMYB2-1   TTGAGCAACTTTGGAGCCTGTTGGATGGAGAAACTGGAACTTG   743
T3-TgBrMYB2-1   TTGAGCAACTTTGGAGCCTGTTGGATGGAGAAACTGGAACTTG   743
T3-TgBrMYB2-1   TTGAGCAACTTTGGAGCCTGTTGGATGGAGAAACTGGAACTTG   743
Consensus       ttgagcaactttggagcctgttggatggagaaactggaacttg
```

Table S1 Primers used in this work.

Gene /Primer name	Forward Primer (5' to 3')	Reverse Primer (5' to 3')	Product (bp)	Gene Number in BRAD	*Arabidopsis* BlastN in NCBI	Utilization Description
QJ-46	TGTCTATCTGACTTGTGGTGTATCC	ATACCTCATAACACTGCATCCATAG	178	——	——	Mapping
LY-2	TTTATTATTGTGGACACCAACCATC	CTTATACAAACTTCTAATGGCAAAGG	123	——	——	Mapping
LY-3	TAAACCTAAAAATACATCTGCTTCC	TTTAACGTGAGAGCTTGAATGC	128	——	——	Mapping
SSR14-36	TGTAATCATCCATAACCATATAAGCG	GGGGGTTGTTGGCATTTTAC	162	——	——	Mapping
BrMYB2	ATGGAGGGTTCGTCCCAAG	TCAAGTTCCAGTTTCTCCATCC	1665/5441	Bra004162	AT1G66390 (*AtPAP2*)	Gene cloning
BrMYB2	GCTCTAGAATGGAGGGTTCGTCCCAAG	GGGGTACCTCAAGTTCCAGTTTCTCCATCC	1681	Bra004162	AT1G66390 (*AtPAP*	Vector constuction (XbaI and

					2)	KpnI)
BrMYB2	CGGGATCCATGGAGGGTTCGTCCCAAG	GGGGTACCTCAAGTTCCAGTTTCTCCATCC	5457	Bra004162	AT1G66390 (*AtPAP2*)	Vector constuction（BamhⅠ and KpnⅠ)
BrP1	TGGTGTACTTTGATCCTTCGTG	ACTGCATTCGCGTCTCCTAC	4080/306	——	——	Co-separation analysis
BrP2	CTTTTCTGCACGAACCCG	ATTCGCGTCTCCTACTCCATAT	4122/348	——	——	Co-separation analysis
BrEF-1-α	ATACCAGGCTTGAGCATACCG	GCCAAAGAGGCCATCAGACAA	116	Bra031605	AT5G60390 (*AtEF-1-α*)	qRT-PCR
BrF3'H	CCATCCACCAACACCACTCT	AGCTTCTCCGGCGTAACTCCTCC	347	Bra009312	AT5G07990 (*AtF3'H*)	qRT-PCR
BrDFR	GACGGCGTTTTCCACATAG	TCCCCAACACTCCATTCAC	94	Bra027457	AT5G42800 (*AtDFR*)	qRT-PCR
BrANS	GAAGACGAAACCATCCCGTGAGA	TTAGCCAACTTACTTCCATACC	220	Bra013652	AT4G22880	qRT-PCR

					(*AtANS*)	
BrMYB2	AGGTGGTCTTTAATTGCT	TCCAAGGCATAGAGGAACAA	257	Bra004162	AT1G66390 (*AtPAP2*)	qRT-PCR
BrTT8	TAGATACACACATGGACATG	TCTTTGACATTCTCAACTCTCCACGA	168	Bra037887	AT4G09820 (*AtTT8*)	qRT-PCR
BrMYB2-Promoter	TGGAACAATGGAAAGATTGTCCAG	GGACCAGCTATAATTTTAGAAGTATCT	2368	——	——	Promoter cloning
BrMYB2-Pro1	CCAAGCTTGGTCTTCTGAGTATAAATGAGCATG	CGGGATCCGGACCAGCTATAATTTTAGAAGTATCT	2272	——	——	Promoter cloning and vector construction
BrMYB2-Pro2	CCAAGCTTGCATAAGATGAGATGGAGAAACTG	CGGGATCCGGACCAGCTATAATTTTAGAAGTATCT	2010	——	——	Promotor cloning and vector construction
BrMYB2-Pro3	CCAAGCTTCTGAACTCCCTAGCCAAAAGTAC	CGGGATCCGGACCAGCTATAATTTTAGAAGTATCT	793	——	——	Promoter cloning and vector construction
BrMYB2-Pro	CCAAGCTTAGTTGT	CGGGATCCGGACC	642	——	——	Promoter

4	GTACTCATACATCAACCCT	AGCTATAATTTTAGAAGTATCT				cloning and vector construction
BrMYB2-Pro5	CCAAGCTTTATTCTGTTTTTAACTCTGGTGCATG	CGGGATCCGGACCAGCTATAATTTTAGAAGTATCT	498	——	——	Promoter cloning and vector construction
AtACT2	CACTTGCACCAAGCAGCATG	GATTCCTGGACCTGCCTCATC	158	——	AT3G18780	qRT-PCR
AtCHS	CGCATCACCAACAGTGAACAC	TCCTCCGTCAGATGCATGTG	101	——	AT5G13930	qRT-PCR
AtCHI	CCGGTTCATCGATCCTCTTC	ATCCCGGTTTCAGGGATACTATC	88	——	AT3G55120	qRT-PCR
AtF3H	CAGATCGTTGAGGCTTGTGAGA	ACGAGTCATATCCGCCACTAAGT	87	——	AT3G51240	qRT-PCR
AtF3'H	GCTCTCGCCGGAGTATTCAA	CCAGCGACGCCTTGTAAATC	74	——	AT5G07990	qRT-PCR
AtDFR	AACGGATGTGACGGTGTTTT	TCCATTCACTGTCGGCTTTA	93	——	AT5G42800	qRT-PCR
AtANS	CGATGAAAAGATCCGTGAGA	GCCAATTTACTTCCATAGCCT	215	——	AT4G22880	qRT-PCR
AtUGT79B1（*UF3GT2*）	CAACTGGTTTTCCGTTTCTGGTT	GCTTCCTCGACGGTTGATACAC	64	——	AT5G54060	qRT-PCR

AtUGT75C1（*5GT*）	CGAAGGCATTACCG TCAGC	GCATCGTGTTCCAA AGCAG	112	——	AT4G1 4090	qRT-PCR
AtUGT78D2（*UF3GT1*）	CACCGCACAATCCA ACTCT	GCATTTATCTCCGT CGCCAT	273	——	AT5G1 7050	qRT-PCR
At5MAT	AGCCACGCTCCTCC ACTATC	ACGGCATCTTTGTC GTCAGG	102	——	AT3G2 9590	qRT-PCR
AtGST	TGGTCGAGGATCTC AAAGTG	TGAATTCTTCACCA GCCAAA	93	——	AT5G1 7220	qRT-PCR
AtMYB11	AAGTGGAGCAGAG GAACCCG	TTGTGCCCAACTGA CATCCC	219	——	AT3G6 2610	qRT-PCR
AtMYB12	TGATGGGGAGTTGC ATAACATA	AACGACTCCACCGA TGGAC	114	——	AT2G4 7460	qRT-PCR
AtMYB111	AATAACAAGACCA AGAAGAAGAAGAA	AGAAACATTGTGA GGCCGTC	92	——	AT5G4 9330	qRT-PCR
AtTT8	TGAATCAACCCATA CGTTAGACA	GGGGTGTGACATG AGAAGTGT	102	——	AT4G0 9820	qRT-PCR
AtGL3	AGTGTTTAGCCGTT CTCTTCTAGC	TGTCTTCCGTAATA TGTTCTGTGG	113	——	AT5G4 1315	qRT-PCR
AtEGL3	TTGGCACGACCGAA CATA	TTGATAGTCTGATC TTGTCGATATTGT	100	——	AT1G6 3650	qRT-PCR
AtTTG1	TCCTCGAAGATTAC AACAACCG	CGGGAGAGGCTTA ACGGTCAT	72	——	AT5G2 4520	qRT-PCR

AtTT2	GCGAAGGCAAATG GAGCACT	CCAAGAAGATTATG GAGACGGA	160	——	AT5G3 5550	qRT-PCR

Br: *B. rapa*; *At*: *Arabidopsis*.

Table S2 MYBs involved in anthocyanin biosynthesis in different species.

Species	Gene name	GenBank/Tair/BRAD ID
Arabidopsis	*ATMYB113*	AT1G66370
Arabidopsis	*ATMYB114*	AT1G66380
Arabidopsis	*AtMYB75* (*PAP1*)	AT1G56650
Arabidopsis	*AtMYB90* (*PAP2*)	AT1G66390
Arabidopsis	*AtTT2*	AT5G35550
Arabidopsis	*AtMYBL2*	AT1G71030
Arabidopsis	*AtMYB11*	AT3G62610
Arabidopsis	*AtMYB12*	AT2G47460
Arabidopsis	*AtMYB111*	AT5G49330
Brassica rapa ‘Chifu’（heading Chinese cabbage‘Chifu’）	*BrMYB2*	Bra004162
Brassica rapa ‘Chifu’（heading Chinese cabbage‘Chifu’）	*BrPAP1*	Bra039763
Brassica rapa ‘Chifu’（heading Chinese cabbage‘Chifu’）	*BrMYB1*	Bra001917
Head Chinese cabbage Line 94S17	*BrMYB2*	Bra004162
purple Chinese cabbage Line 11S/95T	*BrMYB2*	Bra004162
Cauliflower	*BoMYB1*	GU219985
Cauliflower	*BoMYB2*	GU219987
Cauliflower	*BoMYB3*	GU219988
Cauliflower	*BoMYB4*	GU219989
Snapdragon	*AmROSEA1*	ABB83826
Snapdragon	*AmROSEA2*	ABB83827
Snapdragon	*AmVENOSA*	ABB83828
Sweet potato	*IbMYB1*	BAG68211
Sweet potato	*IbMYB2-1*	BAF45116
Sweet potato	*IbMYB2-2*	BAF45117
Sweet potato	*IbMYB2-3*	BAF45118
Sweet potato	*IbMYB2-4*	BAF45119
Tomato	*LeANT1*	AAQ55181
Morning glory	*InMYB1*	BAE94389

Morning glory	*InMYB2*	BAE94709
Morning glory	*InMYB3*	BAE94710
Pepper	*CaA*	CAE75745
Petunia	*PhAN2*	AAF66727
Grape	*VvMYBA1*	BAD18977
Grape	*VvMYBA2*	BAD18978
Grape	*VvMYBA3*	BAD18979
Grape	*VlMYBA1-1*	BAC07537
Grape	*VlMYBA2*	BAC07540
Gerbera	*GMYB10*	CAD87010
Apple	*MdMYB1-1*	ABK58136
Apple	*MdMYB10*	ACQ45201
Apple	*MdMYBA*	BAF80582
Maize	*ZmP1*	AAA19819
Maize	*ZmC1*	AAA33482
Rice	*OsC1*	BAD04024
Black Spruce	*PmMYBF1*	AAA82943
Strawberry	*FaMYB1*	AAK84064
Oncidium Gower Ramsey	*OgMYB1*	ABS58501
Phalaenopsis hybrid cultivar	*PhMyb9*	ACH95795
Diplacus aurantiacus	*MaMYB*	ACA04006
Solanum melongena	*SmMYB*	AGK37072.1
Prunus cerasifera	*PcMYB10*	ABX71495
Prunus cerasifera	*PcMYBA*	ABX71495
Prunus persica	*PpMYB10*	EU155160
Nicotiana tabacum	*NtAN2*	ACO52470
Raphanus sativus	*RsMYB1*	AKM95888

Table S3 Expression patterns of ABGs in Chinese cabbage at head formation period. Seedlings of '94S17' stayed at about 10 DAS were treated as the control in qRT-PCR data analysis. The means are values ±SD. The different letters in each line are significantly different at $p < 0.05$ by Duncan's test.

Gene	**BRAD Gene ID**	94S17-S1	94S17-S2	94S17-S3	94S17-S4	11S91-S1	11S91-S2	11S91-S3	11S91-S4	95T2-5-S1	95T2-5-S2	95T2-5-S3	95T2-5-S4
**Structural genes**													
**Biosynthetic genes in phenylpropanoid pathway**													
BrPAL1.1	Bra005221	0.015±0.003g	0.222±0.092bc	0.304±0.070ab	0.264±0.061ab	0.118±0.006def	0.300±0.019ab	0.138±0.032cde	0.158±0.027cd	0.064±0.017efg	0.042±0.001fg	0.039±0.009fg	0.350±0.000a
BrPAL1.2	Bra017210	0.030±0.006e	0.203±0.035ab	0.098±0.024cde	0.231±0.039a	0.112±0.018cd	0.257±0.035a	0.149±0.021bc	0.209±0.077ab	0.090±0.027cde	0.094±0.005cde	0.064±0.011de	0.221±0.049ab
BrPAL2.1	Bra006985	0.069±0.022e	0.388±0.082e	0.309±0.093e	0.370±0.072e	57.923±5.754cd	200.563±56.764a	120.524±36.399b	100.073±36.074bc	56.185±10.926cd	125.179±0.000b	14.858±0.647de	95.264±22.127bc
BrPAL2.2	Bra039777	0.017±0.002g	0.044±0.010efg	0.116±0.025cd	0.293±0.022a	0.061±0.002e	0.034±0.003efg	0.098±0.015d	0.058±0.022ef	0.219±0.002b	0.028±0.009fg	0.029±0.006fg	0.139±0.015c
BrPAL2.3	Bra003126	0.021±0.002f	0.013±0.002f	0.020±0.006f	0.310±0.070ef	1.097±0.131c	0.318±0.050ef	0.631±0.145de	0.386±0.066ef	7.061±0.505a	1.188±0.168c	0.832±0.208cd	4.009±0.214b
BrPAL3.1	Bra028793	2.294±0.6	0.125±0.087	0.259±0.0	0.067±0.005	5.231±0.686b	1.728±0.223	0.388±0.005	0.093±0.030	19.360±9.35	2.192±0.342	0.762±0.106	0.321±0.094

		59b	b	48b	b		b	b	b	9a	b	b	b
BrPAL3.2	Bra030322	0.038±0.000b	0.014±0.001d	0.000±0.000f	0.022±0.011c	0.017±0.003cd	0.011±0.002d	0.041±0.000b	0.075±0.000a	0.000±0.000f	0.014±0.002d	0.003±0.000f	0.000±0.000f
BrPAL4.0	Bra029831	0.267±0.014c	0.079±0.000c	0.147±0.000c	0.071±0.009c	1.623±0.493a	0.235±0.021c	0.205±0.066c	0.669±0.015b	0.183±0.002c	0.144±0.044c	0.051±0.004c	0.097±0.005c
BrC4H1	Bra018311	0.366±0.022c	0.109±0.001d	0.298±0.023c	0.268±0.061c	0.119±0.029d	1.255±0.215a	0.544±0.036b	0.513±0.000b	0.019±0.007d	0.123±0.006d	0.013±0.002d	0.037±0.004d
BrC4H2	Bra021636	0.016±0.003e	0.119±0.022de	0.802±0.060bc	2.510±0.692a	0.154±0.045de	0.399±0.087cde	0.582±0.161bcd	0.958±0.036b	0.093±0.000de	0.058±0.012e	0.066±0.014e	0.460±0.142cde
BrC4H3	Bra021637	0.032±0.001de	0.023±0.004ef	0.024±0.001ef	0.087±0.007b	0.107±0.023a	0.026±0.001def	0.085±0.017b	0.047±0.013cd	0.060±0.006c	0.007±0.002f	0.026±0.004def	0.026±0.009def
BrC4H4	Bra022802	0.867±0.042d	0.174±0.012ef	1.205±0.000c	1.183±0.237c	1.125±0.125c	2.268±0.005b	3.118±0.000a	0.995±0.203cd	0.382±0.153e	0.039±0.000f	0.202±0.011ef	0.242±0.128ef
BrC4H5	Bra022803	0.019±0.003bc	0.041±0.008abc	0.031±0.008bc	0.061±0.011abc	0.072±0.018ab	0.025±0.008bc	0.029±0.011bc	0.089±0.083a	0.027±0.003bc	0.007±0.001c	0.009±0.005c	0.014±0.006bc
Br4CL1	Bra030429	0.055±0.002b	1.032±0.590a	0.372±0.095b	0.319±0.077b	0.175±0.037b	0.297±0.029b	0.958±0.006a	0.451±0.237b	0.224±0.048b	0.153±0.051b	0.119±0.075b	0.419±0.025b
Br4CL2.1	Bra031262	0.326±0.008e	0.000±0.000e	0.111±0.063e	0.000±0.000e	3.177±1.252de	6.246±0.360c	74.468±4.430a	31.887±0.739b	4.152±0.733cd	0.693±0.113e	0.000±0.000e	0.000±0.000e
Br4CL2.2	Bra031263	0.246±0.003cd	0.428±0.076cd	2.228±0.569a	1.053±0.219b	0.139±0.012d	0.711±0.186bc	0.997±0.061b	1.201±0.475b	0.227±0.066cd	0.035±0.008d	0.053±0.002d	0.234±0.024cd
Br4CL2	Bra	0.0	0.00	0.0	0.01	0.09	0.01	0.00	0.01	0.03	0.00	0.00	0.00

.3	031 265	44±0.005b	2±0.000d	09±0.001cd	0±0.003cd	1±0.035a	6±0.004cd	3±0.001d	6±0.004cd	2±0.005bc	6±0.002d	5±0.000d	6±0.002d
Br4CL2.4	Bra031266	0.348±0.014b	0.366±0.303b	0.308±0.160b	0.436±0.062b	0.123±0.026b	0.366±0.106b	0.830±0.258a	0.734±0.083a	0.210±0.052b	0.143±0.010b	0.303±0.003b	0.232±0.059b
Br4CL3	Bra004109	0.542±0.170b	0.035±0.002b	0.133±0.021b	0.069±0.013b	4.194±1.065a	0.064±0.003b	0.068±0.013b	0.061±0.008b	0.295±0.052b	0.121±0.046b	0.005±0.000b	0.031±0.006b
Br4CL4.1	Bra001819	0.017±0.005e	0.070±0.016bcde	0.007±0.001e	0.101±0.005b	0.096±0.038bc	0.090±0.022bcd	0.217±0.083a	0.028±0.001cde	0.036±0.010bcde	0.020±0.001de	0.008±0.006e	0.101±0.045b
Br4CL4.2	Bra001820	0.095±0.020bc	0.025±0.000bc	0.004±0.001c	0.036±0.005bc	0.107±0.000bc	0.131±0.014b	0.047±0.009bc	0.017±0.004c	0.263±0.154a	0.091±0.006bc	0.064±0.041bc	0.012±0.000c
**Early biosynthetic genes (EBGs)**													
BrCHS1	Bra008792	3.654±0.519c	0.997±0.781c	1.306±0.285c	0.320±0.041c	39.025±16.085a	0.170±0.015c	0.031±0.007c	0.994±0.022c	17.735±6.504b	1.283±0.012c	0.209±0.041c	0.133±0.000c
BrCHS2	Bra006224	4.046±0.565c	1.166±0.299c	4.515±0.097c	0.452±0.138c	93.322±26.301b	0.582±0.060c	0.184±0.093c	6.476±1.354c	122.949±27.149a	14.890±4.813c	5.839±2.141c	2.094±0.000c
BrCHS3	Bra023441	3.598±0.313c	0.177±0.044g	0.371±0.046efg	0.253±0.042fg	29.945±0.000a	1.619±0.014de	0.414±0.018efg	1.561±0.765def	8.179±1.301b	4.267±1.136c	2.328±0.531d	0.338±0.010efg
BrCHS4	Bra036307	0.699±0.012c	0.223±0.055c	0.237±0.015c	0.051±0.019c	35.208±14.213a	0.981±0.332c	0.072±0.008c	0.235±0.038c	14.527±3.267b	4.684±0.784c	0.741±0.049c	0.273±0.205c
BrCHS5	Bra020688	0.175±0.000c	0.000±0.000c	0.000±0.000c	0.000±0.000c	0.127±0.000c	0.240±0.031c	4.653±0.568a	0.746±0.000b	0.106±0.013c	0.000±0.000c	0.000±0.000c	0.000±0.000c

BrCHI1	Bra007142	0.345±0.060d	0.135±0.024ef	0.105±0.019f	0.141±0.020def	1.439±0.194a	0.266±0.036def	0.540±0.138c	0.188±0.008def	1.154±0.181b	0.318±0.021de	0.089±0.014f	0.118±0.019ef
BrCHI2	Bra003209	0.407±0.059c	0.337±0.050c	0.752±0.198bc	0.389±0.047c	1.111±0.066b	0.327±0.018c	1.061±0.762b	0.894±0.105bc	1.910±0.160a	0.622±0.087bc	0.729±0.260bc	0.811±0.133bc
BrCHI3	Bra017728	0.279±0.079b	0.099±0.005b	0.122±0.011b	0.341±0.101b	16.434±7.085a	0.219±0.067b	0.292±0.003b	0.907±0.191b	3.050±0.953b	0.166±0.082b	0.035±0.011b	0.077±0.012b
BrF3H1	Bra036828	0.374±0.029f	0.609±0.054ef	1.285±0.080def	1.062±0.345def	4.739±1.952bc	3.251±0.117cd	20.998±1.786a	0.714±0.025ef	5.933±2.174b	2.922±0.062cde	1.653±0.329def	0.491±0.155f
BrF3H2	Bra029996	0.002±0.000e	0.039±0.000b	0.000±0.000e	0.000±0.000e	0.019±0.001d	0.023±0.003d	0.000±0.000e	0.032±0.007c	0.000±0.000e	0.106±0.000a	0.000±0.000e	0.000±0.000e
BrF3H3	Bra012862	5.653±0.135b	2.326±0.149b	3.570±0.237b	1.196±0.057b	51.027±4.690b	7.307±2.137b	2.563±0.845b	7.672±1.193b	190.193±80.306a	14.245±3.246b	4.179±0.649b	2.742±0.536b
BrF3'H	Bra009312	8.241±0.000c	0.972±0.253c	3.374±0.468c	0.455±0.175c	108.815±48.826b	2.865±1.266c	0.424±0.016c	3.252±1.618c	273.828±63.758a	36.195±4.701c	36.335±21.972c	61.497±57.956bc
BrFLS1	Bra009358	0.793±0.018cd	0.658±0.165cd	2.966±0.274b	2.451±0.338b	0.501±0.123cd	0.314±0.003cd	0.109±0.011d	6.199±1.146a	0.698±0.101cd	0.989±0.200c	0.095±0.017d	0.266±0.004cd
BrFLS2	Bra038647	0.024±0.008d	0.206±0.050b	0.193±0.053b	0.535±0.036a	0.023±0.002d	0.054±0.001d	0.003±0.001d	0.136±0.006c	0.007±0.001d	0.017±0.005d	0.000±0.000d	0.199±0.007b
BrFLS3.1	Bra038648	0.015±0.001c	0.032±0.003b	0.037±0.000b	0.091±0.019a	0.014±0.000cd	0.004±0.001cd	0.006±0.001cd	0.031±0.004b	0.005±0.001cd	0.009±0.002cd	0.001±0.000d	0.009±0.002cd
BrFLS3	Bra	0.0	0.10	0.0	0.03	0.00	0.00	0.00	0.00	0.01	0.00	0.00	0.01

.2	029211	12±0.006bc	9±0.032a	06±0.000c	1±0.004b	4±0.000c	1±0.000c	1±0.000c	1±0.000c	2±0.003bc	1±0.000c	2±0.000c	4±0.000bc
BrFLS3.3	Bra029212	0.012±0.003c	0.179±0.031a	0.007±0.001c	0.059±0.006b	0.004±0.000c	0.002±0.001c	0.000±0.000c	0.003±0.003c	0.014±0.003c	0.003±0.002c	0.006±0.004c	0.073±0.007b
BrFLS4	Bra037747	0.000±0.000f	0.016±0.005cd	0.000±0.000f	0.010±0.009de	0.003±0.000f	0.000±0.000f	0.005±0.000ef	0.000±0.000f	0.001±0.000f	0.018±0.003c	0.048±0.004b	0.327±0.000a
**Late biosynthetic genes (LBGs)**													
BrDFR1	Bra027457	3.961±0.000c	13.035±2.143c	9.530±1.199c	2.172±0.075c	2021.244±266.152a	21.716±0.000c	6.598±0.807c	39.830±1.209c	944.330±97.640b	106.889±12.138c	124.764±19.294c	67.912±15.947c
BrDFR2	Bra019062	0.011±0.000c	0.034±0.000b	0.000±0.000d	0.000±0.000d	0.000±0.000d	0.000±0.000d	0.000±0.000d	0.000±0.000d	0.049±0.009a	0.000±0.000d	0.000±0.000d	0.000±0.000d
BrDFR3	Bra010535	0.510±0.000c	0.000±0.000c	0.143±0.020c	0.716±0.000c	41.015±8.077b	1.714±0.000c	1.311±0.000c	0.775±0.000c	257.521±26.682a	0.016±0.000c	0.000±0.000c	6.368±0.000c
BrANS1	Bra013652	1.293±0.043d	1.285±0.049d	0.818±0.001d	3.114±0.357d	1085.657±332.504b	369.451±36.281c	478.581±7.752c	111.382±52.942d	1554.466±43.861a	571.825±23.648c	107.177±15.729d	85.584±8.248d
BrANS2	Bra019350	0.169±0.033e	0.025±0.009e	0.240±0.029de	1.026±0.063c	2.477±0.098b	0.062±0.023e	0.016±0.002e	0.084±0.004e	7.664±0.685a	0.686±0.174cd	0.010±0.000e	0.404±0.000de
BrANS3	Bra017132	0.000±0.000c	0.000±0.000c	0.000±0.000c	0.000±0.000c	0.410±0.483b	0.000±0.000c	3.809±0.000a	0.088±0.006c	0.027±0.000c	0.000±0.000c	0.000±0.000c	0.000±0.000c
BrANS4	Bra	0.0	0.00	0.4	4.65	0.06	0.00	0.00	1.75	1.13	0.08	3.37	0.00

	000045	00±0.000f	0±0.000f	55±0.009e	4±0.000a	5±0.000f	0±0.000f	0±0.000f	3±0.000c	2±0.009d	9±0.000f	3±0.174b	0±0.000f
BrUF3GT1(BrUGT78D2()	Bra023594	0.768±0.083bcd	0.371±0.021cd	0.373±0.014cd	0.496±0.123cd	1.078±0.182b	1.264±0.359b	2.603±0.507a	0.829±0.073bc	2.477±0.372a	0.315±0.043cd	0.267±0.086d	0.278±0.026d
BrUF3GT2(BrUGT79B1)	Bra003021	0.170±0.041e	0.096±0.005e	0.186±0.073e	0.613±0.209e	51.496±4.893c	1.295±0.103e	0.900±0.145e	9.141±0.714de	324.920±1.323a	92.931±15.194b	9.269±0.559de	12.667±1.273d
BrUF5GT(BrUGT75C1()	Bra038445	0.258±0.133d	0.016±0.005d	0.034±0.010d	0.134±0.038d	5.346±0.529a	0.286±0.012d	0.432±0.066d	0.291±0.070d	5.405±0.896a	2.819±0.815b	1.736±0.074c	0.033±0.004d
Br5MAT	Bra036208	0.123±0.052c	1.187±0.280c	0.202±0.010c	0.263±0.082c	143.840±36.711b	5.324±0.2100.000	4.781±1.061c	1.229±0.227c	364.403±122.972a	31.250±5.647c	8.200±1.045c	2.599±0.536c
Brp-CouT	Bra030550	0.194±0.009d	5.051±0.819d	0.714±0.044d	0.523±0.123d	162.091±13.643b	4.373±0.555d	1.533±0.492d	0.873±0.042d	219.722±16.682a	89.786±38.038c	7.886±1.751d	1.944±0.252d
BrSAT	Bra012153	33.554±2.764c	56.654±2.323b	5.367±0.351e	1.669±0.439e	24.507±2.529d	38.001±10.268c	74.721±8.570a	4.164±0.994e	5.955±0.380e	4.185±0.051e	0.651±0.318e	0.317±0.021e
BrUGT84A1.1	Bra039547	0.023±0.004d	0.165±0.036c	0.073±0.006cd	0.015±0.001d	0.084±0.034cd	0.046±0.004d	0.300±0.047b	0.000±0.000d	0.746±0.110a	0.363±0.081b	0.000±0.000d	0.000±0.000d
BrUGT84A1.2	Bra012784	0.178±0.021g	0.070±0.005g	21.587±1.288a	9.487±0.338c	0.192±0.061g	0.172±0.000g	0.828±0.347fg	4.525±0.085d	3.332±0.859e	11.511±1.054b	4.821±0.189d	1.498±0.078f
BrUGT84A2.1	Bra031290	0.488±0.049d	0.203±0.022e	0.919±0.051c	0.156±0.003e	1.048±0.380bc	0.240±0.075de	2.458±0.000a	1.227±0.130b	0.257±0.038de	0.266±0.007de	0.000±0.000e	0.000±0.000e
BrUGT	Bra	0.3	0.42	8.4	1.12	8.82	0.72	1.58	13.6	2.05	2.08	0.46	0.35

84A2.2	023872	57±0.111c	4±0.077c	54±0.376b	2±0.378c	2±6.983b	5±0.128c	5±0.043c	68±2.317a	7±0.150c	1±0.426c	1±0.031c	3±0.121c
BrUGT84A3.1	Bra039545	0.023±0.003e	0.229±0.043e	2.337±0.229cd	0.614±0.104e	0.152±0.010e	1.330±0.146de	0.788±0.072e	21.671±1.616a	0.222±0.052e	2.875±0.490c	3.294±0.623c	8.088±1.001b
BrUGT84A3.2	Bra039544	0.000±0.000d	0.083±0.000bcd	0.096±0.012bc	0.011±0.001cd	0.001±0.000d	0.025±0.000bcd	0.025±0.000bcd	0.237±0.044a	0.113±0.016b	0.012±0.000cd	0.113±0.016b	0.197±0.122a
**Positive regulators**													
**Independent R2R3-MYB regulatory genes**													
BrMYB12.1	Bra004456	3.910±0.992bc	5.292±0.000b	0.134±0.000e	2.294±3.074cde	5.501±1.714b	8.303±0.710a	1.381±0.076de	0.294±0.136de	2.693±0.038cd	2.164±0.230cde	1.048±0.000de	0.600±0.052de
BrMYB12.2	Bra000453	1.104±0.488b	1.937±0.046b	1.203±0.213b	0.775±0.044b	0.712±0.097b	3.002±0.867b	1.880±1.258b	15.517±5.064a	1.511±0.142b	0.267±0.003b	0.105±0.008b	0.749±0.077b
BrMYB111.1	Bra037419	1.141±0.467bc	0.906±0.092bcd	0.148±0.032d	0.135±0.099d	0.519±0.032bcd	1.303±0.227b	6.985±1.117d	0.338±0.132cd	0.820±0.324bcd	0.107±0.014d	0.038±0.007d	0.059±0.010d
BrMYB111.2	Bra036145	0.120±0.058d	0.158±0.000d	1.381±1.001c	0.945±0.036cd	0.786±0.627cd	0.322±0.171cd	3.903±0.000b	0.119±0.016d	5.434±1.279a	0.731±0.094cd	0.000±0.000d	0.000±0.000d
**R2R3-MYB regulatory genes oartucupate in MBW complex formation**													
BrMYB1	Bra001917	0.817±0.025ef	5.309±1.229a	0.444±0.018f	1.983±0.364cd	0.673±0.030ef	2.930±0.553c	4.216±0.971b	2.734±0.338c	0.298±0.047f	0.877±0.106def	0.755±0.017ef	1.833±0.374cde

BrMYB2	Bra004162	0.693±0.458c	2.885±1.102c	0.515±0.128c	0.314±0.057c	24.164±1.469b	1.908±0.182c	1.205±0.103c	1.599±0.326c	37.749±4.356a	0.886±0.220c	0.920±0.414c	1.841±0.619c
BrPAP1	Bra039763	1.758±0.311bcd	1.064±0.000bcde	0.707±0.215cde	1.062±0.633bcde	0.675±0.000cde	1.904±0.542bc	4.350±1.604a	2.205±0.494b	0.026±0.006e	0.607±0.090cde	0.531±0.337de	1.260±0.006bcde
BrTT2	Bra035532	0.054±0.013d	1.744±0.000a	0.212±0.077c	0.336±0.005b	0.079±0.008d	0.203±0.058c	0.000±0.000d	0.337±0.142b	0.078±0.010d	0.060±0.000d	0.000±0.000d	0.000±0.000d
**bHLH regulatory genes**													
BrTT8	Bra037887	1.717±0.401c	2.584±0.745c	0.511±0.036c	0.221±0.086c	14.122±1.835a	1.877±1.028c	1.406±0.632c	1.442±0.112c	10.580±3.204b	2.331±0.472c	0.345±0.086c	0.697±0.009c
BrEGL3-1	Bra027653	1.321±0.138a	0.037±0.004d	0.003±0.000d	0.000±0.000d	0.300±0.085c	0.029±0.002d	0.000±0.000d	0.000±0.000d	0.863±0.228b	0.101±0.020d	0.000±0.000d	0.000±0.000d
BrEGL3-2	Bra027796	0.975±0.487c	0.710±0.222c	0.942±0.073c	1.206±0.275c	0.409±0.035c	0.849±0.105c	0.712±0.067c	5.418±1.096a	0.622±0.059c	1.226±0.178c	0.333±0.267c	2.355±0.009b
BrGL3	Bra025508	1.575±0.366a	0.103±0.016cd	0.349±0.006cd	0.205±0.003cd	0.091±0.015cd	0.074±0.019d	0.244±0.000cd	0.124±0.044cd	0.737±0.174b	0.374±0.015cd	0.039±0.005d	0.458±0.377bc
**WD40 regulatory genes**													
BrTTG1	Bra009770	0.419±0.038b	0.442±0.101b	0.386±0.056b	0.951±0.195b	0.105±0.014b	0.403±0.066b	2.825±2.582a	0.542±0.000b	0.116±0.034b	0.340±0.021b	0.114±0.012b	1.313±0.000ab
**Negative regulators**													
**Single-Repeat R3 MYB**													

**regulatory genes**													
BrCPC1	Bra004539	0.413±0.015bc	0.491±0.155b	0.276±0.000cdef	0.357±0.024cde	0.205±0.026def	0.172±0.029def	0.114±0.024ef	0.858±0.367a	0.184±0.032def	0.098±0.033ef	0.087±0.041ef	0.044±0.009ef
BrCPC2	Bra039283	1.146±0.005c	1.670±0.000b	0.458±0.082d	1.417±0.252bc	0.542±0.147d	0.475±0.076d	0.358±0.130d	2.081±0.536a	0.387±0.087d	0.160±0.106d	0.135±0.023d	0.206±0.011d
BrMYBL2.1	Bra016164	0.023±0.001c	0.376±0.040a	0.186±0.011b	0.327±0.032a	0.019±0.003c	0.016±0.003c	0.033±0.000c	0.300±0.112a	0.042±0.020c	0.010±0.002c	0.001±0.000c	0.008±0.000c
BrMYBL2.2	Bra007957	0.320±0.128e	5.678±0.375d	25.476±4.231b	10.960±2.654c	0.008±0.000e	1.259±0.193e	3.731±1.113de	12.994±0.184c	0.080±0.016e	0.679±0.000e	1.532±0.410e	31.875±3.734a
**LATERAL ORGAN BOUNDARY DOMAIN (LBD) genes**													
BrLBD37.1	Bra012164	0.455±0.090cde	4.129±1.448a	1.424±0.257bcd	4.422±0.824a	0.521±0.144cde	1.046±0.000bcde	1.644±0.075bc	5.118±1.133a	0.035±0.003e	0.124±0.029de	0.200±0.075de	2.003±0.124b
BrLBD37.2	Bra031833	0.670±0.144bc	0.746±0.154bc	4.543±2.877a	4.976±0.355a	0.065±0.023c	0.208±0.093c	0.136±0.021c	2.280±0.671b	0.025±0.005c	0.820±0.202bc	0.142±0.023c	0.924±0.180bc
BrLBD37.3	Bra037847	2.049±0.419c	7.297±2.113a	5.108±1.307b	6.911±0.140a	0.062±0.003d	0.248±0.012cd	0.660±0.005cd	6.135±1.087ab	0.068±0.012d	0.340±0.129cd	0.264±0.109cd	0.829±0.080cd
BrLBD38.1	Bra036040	0.311±0.115d	3.077±0.388a	0.503±0.032d	0.914±0.159c	0.452±0.075d	0.389±0.068d	0.356±0.096d	1.174±0.081c	0.143±0.028d	0.250±0.078d	0.324±0.045d	1.926±0.452b
BrLBD38.2	Bra012913	2.275±1.2	2.694±0.010	0.184±0.0	0.293±0.017	0.406±0.019b	0.810±0.264	0.745±0.103	0.299±0.074	0.372±0.021	0.253±0.023	0.116±0.002	0.050±0.013

		59a	a	05b	b		b	b	b	b	b	b	b
BrLBD39.1	Bra011772	5.121±1.251d	112.654±14.678ab	50.916±2.621c	94.572±12.949b	21.109±0.683cd	8.501±2.475cd	132.983±37.224ab	147.287±47.729a	1.354±0.193d	11.542±3.125cd	4.935±0.271d	50.500±17.371c
BrLBD39.2	Bra017831	0.432±0.065d	4.622±0.420a	1.433±0.087c	0.244±0.059de	1.165±0.086c	0.241±0.107de	2.000±0.037b	0.246±0.054de	0.106±0.030e	0.179±0.022de	0.063±0.013e	0.047±0.009e
**Transport genes**													
BrGST1	Bra008570	0.483±0.058c	1.645±0.020c	0.874±0.775c	1.517±0.458c	193.324±59.890b	3.739±0.671c	11.578±0.133c	4.583±1.806c	659.127±111.830a	68.972±16.487c	78.809±5.632c	35.528±3.708c
BrGST2	Bra023602	0.116±0.002c	0.155±0.035c	0.014±0.007c	0.052±0.016c	12.780±2.323b	0.082±0.020c	0.041±0.006c	0.124±0.028c	17.036±1.586a	1.291±0.404c	1.021±0.169c	0.314±0.069c
